# The Involvement of Secondary Neuronal Damage in the Development of Neuropsychiatric Disorders Following Brain Insults

**DOI:** 10.3389/fneur.2014.00022

**Published:** 2014-03-11

**Authors:** Yun Chen, Gregory E. Garcia, Wei Huang, Shlomi Constantini

**Affiliations:** ^1^BrightstarTech Inc., Clarksburg, MD, USA; ^2^US Army Medical Research Institute of Chemical Defense, Aberdeen Proving Ground, Aberdeen, MD, USA; ^3^Uniformed Services University of the Health Sciences, Bethesda, MD, USA; ^4^Department of Pediatric Neurosurgery, Dana Children’s Hospital, Tel Aviv Medical Center, Tel Aviv University, Tel Aviv, Israel

**Keywords:** neuropsychiatric disorders, secondary neuronal damage, brain insults, traumatic brain injury, neurodegenerative diseases, clinical manifestations, histomorphology, pathophysiology

## Abstract

Neuropsychiatric disorders are one of the leading causes of disability worldwide and affect the health of billions of people. Previous publications have demonstrated that neuropsychiatric disorders can cause histomorphological damage in particular regions of the brain. By using a clinical symptom-comparing approach, 55 neuropsychiatric signs or symptoms related usually to 14 types of acute and chronic brain insults were identified and categorized in the present study. Forty percent of the 55 neuropsychiatric signs and symptoms have been found to be commonly shared by the 14 brain insults. A meta-analysis supports existence of the same neuropsychiatric signs or symptoms in all brain insults. The results suggest that neuronal damage might be occurring in the same or similar regions or structures of the brain. Neuronal cell death, neural loss, and axonal degeneration in some parts of the brain (the limbic system, basal ganglia system, brainstem, cerebellum, and cerebral cortex) might be the histomorphological basis that is responsible for the neuropsychiatric symptom clusters. These morphological alterations may be the result of secondary neuronal damage (a cascade of progressive neural injury and neuronal cell death that is triggered by the initial insult). Secondary neuronal damage causes neuronal cell death and neural injury in not only the initial injured site but also remote brain regions. It may be a major contributor to subsequent neuropsychiatric disorders following brain insults.

Neuropsychiatric disorders, also called mental disorders or emotional disorders, are conditions that affect cognition, emotion, behavior, and substantially interfere with the ability to function (1). Clinical manifestations of neuropsychiatric disorders include memory and cognitive deficits, consciousness and sleep disturbances, mental and emotional symptoms, somatoform symptoms, and psychomotor performance deficits. Neuropsychiatric disorders are 4 of the 10 leading causes of disability worldwide (2). About 26.2% of the U.S. adult population and 27% of the European Union adult population aged 18 and older, meet the diagnostic criteria for neuropsychiatric disorders at some point in their lives (3, 4). Four hundred fifty million people worldwide were diagnosed with some form of neuropsychiatric disorder in 2001 (5).

The mechanism underlying neuropsychiatric disorders has not been fully elucidated and still remains controversial. Several different factors may be involved in the occurrence and development of neuropsychiatric disorders. Psychological (violent attacks, being abused or neglected, marital separation, divorce, the sudden death of a loved one, being unable to relate to others, etc.), biological (genetics, prenatal damage, infections, exposure to toxins, brain defects or injuries, chemical imbalances, and substance abuse), and social/environmental (such as poor social and work relationships, family argument, dispute at work, loss of job, retirement, unemployment or underemployment, serious financial problems, poverty, socioeconomic deprivation, and substance abuse) factors are usually considered to be the triggers that cause most neuropsychiatric disorders (6–11). Research thus far does not clearly demonstrate how these triggers can cause neuropsychiatric disorders in the brain, nor what the cellular and molecular changes associated with the mental diseases are. The histomorphological basis of a neuropsychiatric disorder has been generally ignored.

Every disease has its own histomorphological features. A disease will not occur without morphological alterations associated with the disease in cells, tissues, and organs. When the cause and pathomorphology of a disease are poorly understood, people usually mythologize the disease and attribute the symptoms to social and cultural problems such as a sedentary lifestyle, social isolation, depressed mood, economic crisis, overindulgence in sex, obesity, heredity, etc. For example, tuberculosis had been considered as a fatal wasting disease (consumption) caused by vampires before *Mycobacterium tuberculosis* was discovered in 1882 (12). Therefore, definition of morphological alterations at the cellular and molecular levels is a very important step in the diagnosis and treatment of the disorder in general. A recent study has suggested that neuropsychiatric disorders triggered by psychological and social/environmental factors may involve a volumetric blood surge created by a sudden rise in cardiovascular pressure after exposure to an emotionally and psychologically traumatic event. The volumetric blood surge will move quickly through blood vessels to the brain, dramatically increasing cerebral perfusion pressure and causing damage to both very small cerebral blood vessels in the brain and to the blood brain barrier (BBB) (13). This indicates that brain microstructural damage could be the histomorphological basis of neuropsychiatric disorders triggered by psychological and social/environmental factors.

In the present study, a clinical symptom-comparing approach was employed to identify the similarities and differences among the neuropsychiatric manifestations related to 14 different types of brain insult. To explore the potential mechanisms that cause neuropsychiatric disorders, the correlation between neuropsychiatric manifestations and pathomorphology of the brain was analyzed.

## Brain Insults Selected for Study

Neuropsychiatric disorders frequently occur after various brain insults such as closed head injury (CHI) (caused by external physical forces), blast-induced traumatic brain injury (TBI) (caused by blast shock waves), stroke (including ischemic and hemorrhagic stroke), poisoning with hazardous chemicals [caused by organophosphate (OP) pesticides, chemical nerve agents, chemical neurotoxic substances, alcohol, drugs, etc.), infection by pathogenic microbes (such as HIV/AIDS and PANDAS), brain tumors (including gliomas, meningiomas, pituitary adenomas, and nerve sheath tumors), and degenerative diseases [such as Parkinson’s, Alzheimer’s, amyotrophic lateral sclerosis (ALS), and Huntington’s]. Although these brain insults can induce many of the same neuropsychiatric disorders, their initial causes of injury are totally different (Table 1).

**Table 1:** **The initial causes of brain insults**.

Brain insult	Cause of injury
Stroke	Progressive neuron death caused by disturbance in the blood supply to the brain (ischemia or hemorrhage)
Brain tumor	A tumor (an abnormal growth of cells) within the brain

**TRAUMATIC BRAIN INJURY (TBI)**	
Blast-induced TBI	Progressive neuron death caused by blast shock waves
Closed head injury	Progressive neuron death caused by external physical force(s)

**NEURODEGENERATIVE DISEASES**	
Parkinson’s	Greatly decreased activity of dopamine-secreting cells caused by progressive neuron death in the basal ganglia
Alzheimer’s	Progressive neuron death and synapses loss in the cerebral cortex, limbic system, and the brainstem
Amyotrophic lateral sclerosis	Progressive death of motor neurons in the cortex and the brainstem, characterized by proteinaceous inclusions in neuron bodies and axons
Huntington’s (Chorea)	An autosomal dominant mutation in Huntington gene on chromosome 4 causes cellular accumulation of protein clumps, inducing cellular toxicity, and progressive neuron death primarily in the basal ganglia and the cortex

**POISONING WITH HAZARDOUS CHEMICALS**	
Organophosphate pesticides/chemical nerve agents	Cholinergic neuronal deficits induced by irreversible AChE inhibition
Chemical neurotoxic substances	Progressive neuron death caused by neurotoxicity-induced ionic imbalance in neurons
Alcohol abuse	Progressive neuronal damage resulting from the combination of prolonged ingestion of alcohol, which may involve abnormal release of neurotransmitters and prolonged stimulation of reward circuitry
Drug abuse	The release and prolonged action of dopamine and 5-HT within the reward circuit of the brain activate the reward system and lead to drug addiction, which cause chronic neuropathy in the brain

**INFECTIONS BY PATHOGENIC MICROBES**	
HIV/AIDS	Progressive neuron death caused by the harmful inflammatory cascades including activation of macrophages and microglia, release of inflammatory mediators, and induction of uncontrolled inflammation
PANDAS	An autoimmune-mediated response to a streptococcal infection, which produces antineuronal antibodies to erroneously destroy normal neurons in the basal ganglia

### Acute brain insults

#### Closed head injury

Closed head injury is a type of TBI caused primarily by motor vehicle accidents, sporting activities, accidental falls, assaults, etc., where the skull and dura mater remain intact. CHI can range from mild to severe. CHI causes initial damage only to specific brain parts, the impact site or opposite side of the skull. Post-injury pathophysiological changes are usually observed in areas bordering the injured sites, and not in the entire brain (14, 15).

#### Blast-induced TBI

Blast-induced TBI is a unique type of brain injury that is caused by blast shock waves in the victims who are exposed to a blast but do not sustain penetrating and blunt impact injuries. The vast majority (~90%) of blast-induced TBI cases are mild. The major mechanism of blast TBI may involve large-scale BBB damage and tiny cerebrovascular insults in the entire brain, which are caused by the blood surge moving quickly through large blood vessels to the brain from the torso (16).

#### Stroke

Stroke is a brain damage characterized by the sudden disruption of blood supply to an area of the brain and the rapid loss of brain function. It is the second leading cause of death worldwide. Stroke can be caused by an obstruction in the blood flow (ischemic stroke) or the rupture of a blood vessel that supplies the brain (hemorrhagic stroke). A loss of blood supply to part of the brain will initiate a pathophysiological cascade that leads to irreversible death of neuronal cells and permanent neurological dysfunction. The symptoms depend on the area of the brain affected, but generally symptoms include sudden loss of the ability to speak, memory and cognitive deficits, visual field defect, and hemiplegia (17, 18).

#### Organophosphate pesticides/chemical nerve agents

Organophosphate compounds are a group of highly toxic chemicals that have been used widely as pesticides and developed as chemical warfare nerve agents such as soman (GD), sarin (GB), tabun (GA), cyclosarin (GF) and VX. OP pesticides/chemical nerve agents disrupt the functioning of cholinergic nervous system by irreversibly inhibiting acetylcholinesterase (AChE). The inhibition of AChE by OPs results in both the accumulation of ACh at synapses of the central and peripheral nervous systems and overstimulation of cholinergic receptors that exceeds normal physiological limits. Acute, excessive stimulation of cholinergic receptors (mAChR, in the brain) causes cholinergic neuronal excitotoxicity and dysfunction, which are largely responsible for the cholinergic crisis in the acute phase of the OP exposures, and could subsequently cause brain damage and chronic neuropsychiatric consequences (19).

#### Chemical neurotoxic substances

Chemical poisoning is a condition in which a harmful chemical causes damage to human cells, tissues, or organs, usually by chemical reaction or other activity at the molecular level after a sufficient amount is absorbed by the human body. Chemical poisoning is a major public health concern and the second leading cause of injury and death worldwide (20, 21). Chemical poisoning can be caused by approximately 3,000 hazardous chemicals.

Chemical neurotoxic substances (such as acetone, benzene, lead, mercury, and strychnine) cause damage to the brain by disrupting ionic balance in neuronal cells, interrupting neural signal transmission between neurons, altering normal brain function, and inducing progressive neuronal cell death and neurodegeneration. Clinical manifestations may vary among the victims who are exposed to chemical neurotoxic substances, depending on the specific compound, the amount, the route, and the length of time of exposure, as well as on the age and health status of the person exposed (22, 23).

#### HIV/AIDS

The brain can be infected by pathogenic microbes such as bacteria and viruses. Infections by pathogenic microbes can cause harmful inflammatory cascades in the brain, because cerebral cellular infiltration in response to microbial invasion is weaker, and delayed and inflammatory response in the brain is much stronger and spreads more rapidly than in other organs or tissues (24). Therefore, an overactive immune response to microbial invasion in the brain should be the major contributor to infection-related brain damage.

HIV/AIDS is a disease of the human immune system caused by infection with HIV. Although the immune system is the principal target of HIV, the brain can be also affected by HIV. HIV-associated psychiatric disorders have been observed in more than 50% of patients with HIV/AIDS (25). These neuropsychiatric disorders may be caused by progressive neuronal cell death resulting from the harmful inflammatory cascades, including activation of macrophages and microglia to release inflammatory mediators such as cytokines, eicosanoids, and complements, and overstimulation of the immune system to lead to uncontrolled inflammation in the brain (26, 27).

#### Pediatric autoimmune neuropsychiatric disorders associated with streptococcal infections

Pediatric autoimmune neuropsychiatric disorders associated with streptococcal infections (PANDAS) are neuropsychiatric disorders [usually obsessive–compulsive disorder (OCD) and/or tic disorders] following a Group A beta-hemolytic streptococcal infection (GABHS) in children (28, 29). The mechanism behind PANDAS may involve an autoimmune-mediated response to a streptococcal infection, which produces antineuronal antibodies to erroneously destroy normal neuronal cells in the basal ganglia (30).

### Chronic brain insults

#### Brain tumor

Brain tumor is an intracranial solid neoplasm that is formed by an accumulation of abnormal growth cells. The most common types are glioma and astrocytic tumors from abnormal growth glial and astrocytes cells. The second most common type of brain tumor is meningeal tumor that forms in the meninges. Pituitary adenomas are the third most common type of brain tumor; these are non-cancerous tumors of the pituitary gland. Nerve sheath tumors are made up primarily of the myelin surrounding nerves and account for approximately 8% of all brain tumors. The symptoms of brain tumors mainly depend on tumor size and tumor location, but common clinical consequences include symptoms of intracranial hypertension, neurological and neuropsychiatric disorders, and psychomotor performance deficits (31–34).

#### Alcohol abuse

Alcohol abuse is an addictive illness that is caused by uncontrolled consumption of alcoholic beverages. Prolonged ingestion of alcohol will affect brain function by altering levels of neurotransmitters (GABA, glutamate, and dopamine) and inducing prolonged stimulation of reward circuitry, thus causing progressive brain damage. The brain areas that are commonly affected by alcohol are the cerebral cortex, cerebellum, hypothalamus and pituitary, and medulla. In cerebral cortex, alcohol depresses the behavioral inhibitory centers, inhibits the thought processes, and slows down the processing of information from the eyes, ears, mouth, and other senses. This will cause the slowed reaction times, impaired memory, blurred vision, slurred speech, etc. If alcohol affects the cerebellum (the center of movement and balance), it leads to the “staggering gait of a drunk” or difficulty walking. Alcohol depresses the hypothalamus and pituitary to increase sexual urge, but decrease actual sexual performance. The hypothalamus and pituitary control sexual arousal and performance by coordinating automatic brain functions and hormone release. By acting on the medulla, alcohol induces sleepiness and deficits of consciousness, slows breathing, and decreases body temperature, which may cause death (35, 36).

#### Drug abuse

Drug abuse, also known as substance abuse, is a disorder that is caused by using an illicit drug to feel happy and excited, to be more productive or to help one stay awake, to improve athletic performance, or to ease another mental and emotional problem (such as stress, anxiety, or depression) (37, 38). Drug abuse has led to significant physical, psychological, and public health problems in almost every country. About 200 million people around the world use illegal drugs every year. More than 22 million Americans aged 12 and older (nearly 9% of the U.S. population) used illegal drugs in 2011 (39). The mechanism by which illicit drugs cause brain damage remains unknown. Some drugs may cause the neuronal cells to release abnormally large amounts of dopamine and 5-HT and produce prolonged enhancement of synaptic transmission within the reward circuit of the brain, thus causing progressive brain damage (40, 41).

MDMA (or called ecstasy) is an empathogenic drug of the phenethylamine and amphetamine classes of drugs. The effects of MDMA on human brain are complex. MDMA may cause the neuronal cells to release 5-HT, dopamine, and norepinephrine, and may act directly on a number of receptors, including α2-adrenergic and 5-HT2A receptors (42). MDMA can result in serotonergic neurotoxicity and psychiatric and behavioral problems such as memory loss, cognitive deficit, moodiness, aggression, anxiety, sleep disorder, hypersexuality, and increased sensitivity to pain (43). Cocaine is a crystalline tropane alkaloid obtained from the coca plant, and a CNS stimulant. It acts as both a serotonin–norepinephrine–dopamine reuptake inhibitor and a triple reuptake inhibitor, and affects the mesolimbic reward pathway to cause addiction (44). Cocaine blocks the reuptake of serotonin, norepinephrine, and dopamine by the neurons that release it, allowing higher concentrations of the neurotransmitters to remain in the synapse for an extended period of time. This abnormally long presence and high concentration of neurotransmitters in the synapse will lead to brain dysfunction and damage in the particular parts of the brain such as the basal ganglia and the limbic system, thus causing psychiatric and behavioral problems including memory and cognitive deficits, dysphoria, depression, insomnia or hypersomnia, psychomotor retardation or agitation, elevated mood, supremacy feeling, irritability, paranoia, restlessness, anxiety, dilated pupils, excited and exuberant speech, etc. (45–47). Methamphetamine is a neurotoxin and potent psychostimulant of the phenethylamine and amphetamine classes. It is directly neurotoxic to both dopamine and serotonin neurons and can cause brain damage from long-term use in humans (48). This brain damage leads to adverse changes in brain structure and function such as reductions in gray matter volume in several brain regions and adverse changes in markers of metabolic integrity. The neuropsychiatric effects of methamphetamine include euphoria, dysphoria, anxiety, depression, elevated mood, changes in libido, alertness, apprehension, impaired concentration, decreased sense of fatigue, insomnia, self-confidence, sociability, irritability, restlessness, psychosis, suicide, violent behaviors, and repetitive and obsessive behaviors (49). Marijuana, also known as Cannabis, is a psychoactive drug that is often consumed for heightened mood, euphoria, and relaxation. Marijuana has a role in the brain’s control of memory, cognition, and movement. It interacts with the brain’s endogenous opioid system and affects dopamine transmission (50). Marijuana use is associated with psychiatric and behavioral problems (such as memory and cognitive deficits, anxiety, depression, paranoia, auditory and visual hallucination, reddening of the eyes, impaired motor skills), and increases a risk for mental disorders (such as schizophrenia, depersonalization disorder, and bipolar disorders) (51).

#### Parkinson’s disease

Neurodegenerative diseases are characterized by the progressive neuron death/loss and synapses loss in some brain regions such as the cortex, brainstem, and basal ganglia, resulting in the progressive deterioration of neurological and neuropsychiatric symptoms, permanent disability, and death. Parkinson’s disease is the result of death and loss of dopamine-secreting cells in the basal ganglia. Progressive dopamine-secreting cell death causes a marked decrease in dopamine levels of the brain that induces obvious motor symptoms including shaking, rigidity, slowness of movement, and difficulty with walking and gait at the early stage of the disease. Later in the course of the disease, neuropsychiatric symptoms such as depression, memory and cognitive deficits, emotional symptoms, and other behavioral problems are commonly observed in most patients (52–55).

#### Alzheimer’s disease

Alzheimer’s disease is an irreversible, progressive neurologic disease of the brain that slowly causes memory loss, cognitive impairments, and other mental illnesses (53). It is characterized by progressive neuron death and loss of synapses in the cerebral cortex, limbic system, and the brainstem (56, 57). An abundance of amyloid plaques and neurofibrillary tangles in the areas of the brain affected is a typical feature of Alzheimer’s disease (58).

#### Amyotrophic lateral sclerosis

Amyotrophic lateral sclerosis is a degenerative motor neuron disease characterized by the gradual degeneration and death of motor neurons in the cortex and the brainstem. ALS causes rapidly progressive weakness, muscle atrophy, fasciculations and spasticity, dysarthria, dysphagia, and dyspnea. Some patients with ALS have cognitive and memory deficits, depression, and other mental and emotional disorders (59, 60).

#### Huntington’s disease

Huntington’s disease (HD), also called Huntington’s chorea, is an inherited neurodegenerative disorder that is caused by an autosomal dominant mutation in Huntington gene on chromosome 4. The Huntington gene mutation causes cellular accumulation of protein clumps, inducing cellular toxicity and progressive cell death primarily in the basal ganglia and the cortex (61–63). HD affects the individual’s muscle coordination, judgment, memory, and other cognitive function, thus leading to uncontrolled movements, cognitive and memory deficits, and other neuropsychiatric disorders (64–66).

## Comparison of Neuropsychiatric Manifestations of Brain Insults

Clinical manifestations of neuropsychiatric disorders include both signs and symptoms. The neuropsychiatric signs and symptoms indicate the presence of disease or abnormality in the brain. Some signs and symptoms (such as fatigue, nausea, vomiting, and hypersexuality) may be non-specific and can occur in many acute and chronic medical conditions, whereas most signs and symptoms are fairly specific for a brain disease or injury (such as memory loss, cognitive impairments, consciousness loss, anxiety, depression, and psychosis). The specific signs and symptoms should be meaningful and significant in assisting the diagnosis of brain insult(s).

The clinical symptom-comparing approach that was employed in the present study is a clinical research method to determine the same signs and symptoms that are commonly shared by two or more brain insults. Through literature search and review, the signs and symptoms relating to these brain insults are identified and categorized into different categories. All signs or symptoms identified are compared among different types of brain insults using Microsoft Excel. The same sign or symptom will be identified if this sign or symptom is commonly presented in all types of brain insults. A meta-analysis will be used to determinate whether the same signs or symptoms exist in these brain insults and what the effect size of the same signs or symptoms is.

### Literature search on neuropsychiatric sign/symptom clusters of brain insults

Articles relating to the neuropsychiatric signs and symptoms of the 14 types of acute and chronic brain insults were searched between the years 1970 and 2013 using NIH PubMed database for keywords such as “Stroke,” “Patient,” and “Memory loss.” A neuropsychiatric sign or symptom that was reported or described in at least three different articles was selected as a common sign or symptom for the brain insult. Fifty-five common signs or symptoms were identified for the 14 types of brain insult (Supplementary Table 1). They are categorized into five major categories: memory and cognitive deficits (memory loss, concentration difficulties, cognitive impairments, learning disabilities, etc.); consciousness and sleep disturbances (sleep disorder, insomnia, consciousness loss, etc.); mental and emotional symptoms (anxiety, depression, aggression, moodiness, etc.); somatoform symptoms (headache, reduced visuo-spatial abilities, blurred vision, fatigue, etc.); and impaired psychomotor and neuromotor functions (psychomotor retardation, lack of motor coordination, difficulty balancing, seizures/tremors, etc.) (Table 2).

**Table 2:** **The same neuropsychiatric signs and symptoms commonly shared by 14 types of brain insult**.

Brain insult		Category of neuropsychiatric signs and symptoms																					
		Memory and cognitive deficits	Consciousness and sleep disturbances	Mental and emotional symptoms	Somatoform symptoms	Impaired psychomotor and neuromotor functions
**Acute brain insult**	Closed head injury	Memory loss Concentration difficulties Cognitive impairments Learning disabilities Forgetfulness Impaired attention	Sleep disorder Insomnia	Anxiety Depression Aggression/Agitation Moodiness Psychosis Increased emotional sensitivity	Headache Reduced visuo-spatial abilities Blurred vision Fatigue	Psychomotor retardation Lack of motor coordination Difficulty balancing Seizures/tremors
	Blast-induced TBI					
	Stroke					
	OP/chemical nerve agents					
	Chemical neurotoxic substances					
	HIV/AIDS					
	PANDAS					

**Chronic brain insult**	Brain tumor					
	Alcohol abuse					
	Drug abuse					
	Parkinson’s					
	Alzheimer’s					
	Amyotrophic lateral sclerosis					
	Huntington’s (Chorea)					
**The brain regions/structures may be involved in**		The limbic system (the hippocampus, the amygdala, the cingulate gyrus, the thalamus, the hypothalamus, the epithalamus, the mammillary body), basal ganglia system (the striatum), the cerebellum, and the cerebral cortex	The brainstem reticular formation, basal forebrain, hypothalamus, thalamus, and the cerebral cortex	The limbic system (the hippocampus, the amygdala, the cingulate gyrus, the thalamus, the hypothalamus, the epithalamus, the mammillary body), basal ganglia system (ventral striatum), the cerebellum, and the cerebral cortex	The cerebral cortex (dorsolateral prefrontal, insular, rostral anterior cingulate, premotor, parietal cortices, and parahippocampal gyrus)	The cerebral cortex (the posterior parietal, the primary motor, the premotor, and the supplementary motor cortices), the basal ganglia system, and the cerebellum

### Identification of neuropsychiatric signs/symptoms commonly shared by all of the 14 brain insults

**Figure 1:**
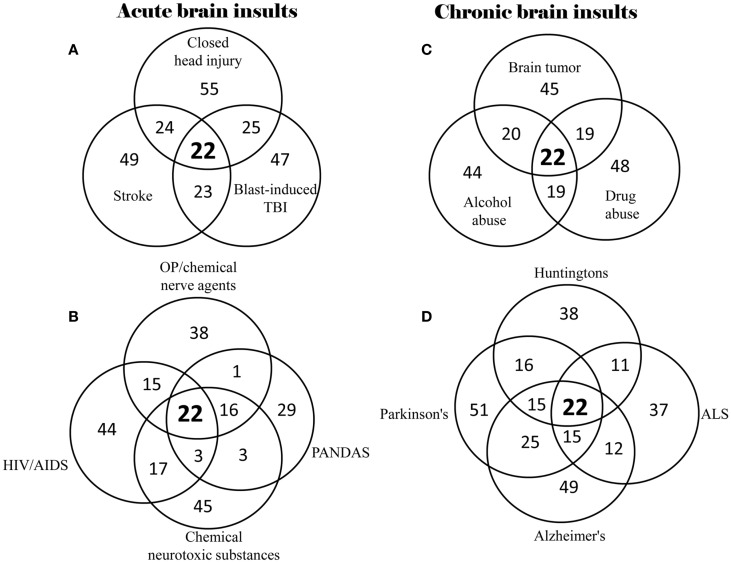
**Overlap of neuropsychiatric symptom clusters among different types of brain insults**. **(A)** Overlap of symptom clusters among closed head injury, blast-induced TBI, and stroke; **(B)** Overlap of symptom clusters among OP/chemical nerve agents, chemical neurotoxic substances, HIV/AIDS, and PANDAS; **(C)** Overlap of symptom clusters among brain tumor, alcohol abuse, and drug abuse; and **(D)** Overlap of symptom clusters among Parkinson’s disease, Alzheimer’s disease, ALS and Huntington’s disease.

Based on the 528 supplementary references in the Supplementary Material, 40% (22) of the 55 neuropsychiatric signs and symptoms were found to be commonly shared by all of the 14 brain insults, whether traumatic, infectious, toxic, oncogenic, or degenerative (Table 2). In the category “memory and cognitive deficits,” six symptoms (memory loss, concentration difficulties, cognitive impairments, learning disabilities, forgetfulness, and impaired attention) are shared; in the category “consciousness and sleep disturbances,” two symptoms (sleep disorder and insomnia) are shared; in the category “mental and emotional symptoms,” six symptoms (anxiety, depression, aggression/agitation, psychosis, moodiness, and increased emotional sensitivity) are shared; in the category “somatoform symptoms,” four symptoms (headache, reduced visuo-spatial abilities, blurred vision/visual field impairment, and fatigue) are shared; and in the category “impaired psychomotor and neuromotor functions,” four symptoms (psychomotor retardation, lack of motor coordination, difficulty balancing, and seizures/tremors) are shared. Overlap of neuropsychiatric symptom clusters among different brain insults is shown in Figure 1. The 22 of the same neuropsychiatric manifestations were simultaneously identified in either acute (Figures 1A,B) or chronic (Figures 1C,D) brain insults. Many overlapping neuropsychiatric signs or symptoms were also found between each of the brain insults. This suggests that neuronal damage may be caused by these brain insults in the same or similar brain regions/structures or that the same or similar brain regions/structures can be possibly affected by different brain insults.

**Figure 2:**
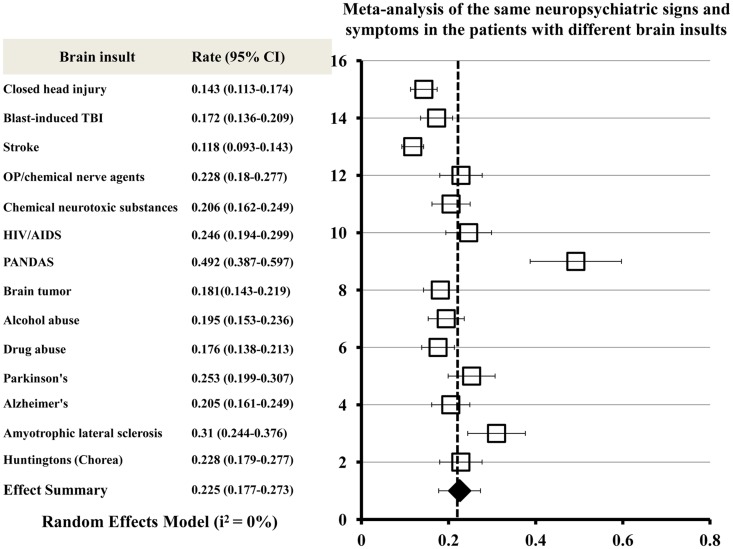
**Meta-analysis of the same neuropsychiatric signs and symptoms shared by all acute and chronic brain insults**. Forest plots of the meta-analysis show effect size (the rate) and 95% confidence intervals for randomized. The overall combined effect size is indicated by a diamond. Heterogeneity *I*^2^ = 0% for all brain insults combined.

A meta-analysis was conducted to combine clinical manifestations of different brain insults and to determinate the neuropsychiatric signs or symptoms commonly shared by all acute and chronic brain insults. The meta-analytic procedure adopted for this systematic study was based upon the methods described by Neyeloff et al. (67). The total numbers of patients presenting with all of the same neuropsychiatric signs or symptoms were used as the sample size for each brain insult. A matrix of sample-size weighted similarity coefficients between each of the 14 types of brain insults was computed in the meta-analysis. Forest plots of the meta-analysis give the effect estimate (the rate) and its 95% confidence interval for each insult. The overall combined effect size is indicated by a diamond. The meta-analysis of neuropsychiatric manifestations of the patients with brain insults shows that the same neuropsychiatric signs or symptoms exist in the 14 different types of brain insults (Figure 2).

### Identification of neuropsychiatric signs/symptoms commonly shared by more than 10 different types of brain insult

Other neuropsychiatric signs or symptoms were found to be commonly shared by more than 10 different types of brain insult: 6 neuropsychiatric signs or symptoms (impaired judgment, confusion, disorientation, fear/frightened, behavior stress, and altered hearing and ringing in the ears) are shared by 13 brain insults; 2 neuropsychiatric signs or symptoms (dementia, and unusual temper tantrums/irritability) are shared by 12 brain insults; 4 neuropsychiatric signs or symptoms (mania, suicidal thoughts/attempts, nausea, and vomiting) are shared by 11 brain insults; and 5 neuropsychiatric signs or symptoms (consciousness loss, restlessness, hallucinations, dizziness, and aphasia) are shared by 10 brain insults (Supplementary Table 1). Interestingly, the symptom of suicidal thoughts or attempts was commonly found in the cases of 11 different types of brain insult (CHI, blast-induced TBI, stroke, poisoning due to chemical neurotoxic substances, HIV/AIDS, alcohol abuse, drug abuse, Parkinson’s disease, Alzheimer’s disease, ALS, and HD). This suggests that suicidal thoughts or attempts may be a result of brain damage. A patient who has attempted suicide may be suffering from degeneration or death of neuronal cells in the brain, and need medical interventions for potential brain damage.

Post-traumatic stress disorder (PTSD) was found to be commonly shared by nine different brain insults (CHI, blast-induced TBI, stroke, poisoning with OP pesticides/chemical nerve agents, poisoning due to chemical neurotoxic substances, HIV/AIDS, brain tumor, alcohol abuse, and drug abuse). PTSD is characterized by persistent re-experiencing symptoms, avoidance symptoms, and hyperarousal symptoms. It is closely associated with some brain insults such as TBI, stroke, brain tumor, and OP poisoning. PTSD symptoms developed in 50–80% of mild TBI patients (16), 25–50% of stroke survivors (68), 35% of childhood brain tumor survivors (69), and 8–23% of the victims of OP poisoning (70). PTSD is most likely to be a special delayed-onset neuropsychiatric disorder caused directly by brain injury or disease, not caused by exposure to traumatic stress.

## Correlation between Clinical Manifestations and Histomorphological Alterations of the Brain

A neuropsychiatric disorder should have its morphological alterations in the brain. Any brain insult can certainly cause histomorphological, biochemical, or molecular biological alterations in some parts of the brain, such as the limbic system, basal ganglia system, the brainstem, the basal forebrain, the cerebellum, and the cerebral cortex. Unfortunately, these alterations at the cellular and molecular level are usually not easy to detect by using traditional diagnostic imaging techniques (e.g., ultrasound, X-rays, CT, and MRI), because tiny morphological changes (such as neuronal cell death or loss) in deep brain structures (such as the limbic system and basal ganglia system) can be often covered by other normal neural tissue layers of the brain. Much of the neuronal damage in deep brain structures can be seen only under a microscope after death (13).

### Morphological alterations of neuropsychiatric disorders

Recently, morphological alterations of some neuropsychiatric disorders (such as schizophrenia, depression, bipolar disorder, and drug abuse) have been verified in patients and animal models by using structural and functional brain imaging technologies and histopathological examinations. Reductions in brain volume have been reported in areas of the frontal cortex, temporal lobes, and brain stem in patients with Schizophrenia (71–74). Morphological abnormalities in frontal–striatal–temporal areas (75), hippocampus (76), and the brain stem (72) have been observed in patients with depression and in a female monkey model of depression. Meta-analyses of structural MRI studies in bipolar disorder show that there is an increase in both the volume of the lateral ventricles and globus pallidus, and the rates of deep white matter hyperintensities (77, 78). Functional MRI findings suggest that abnormal modulation between ventral prefrontal and limbic regions, especially the amygdala, likely contribute to poor emotional regulation and mood symptoms in the bipolar disorder patients (79). Morphological abnormalities in the brain stem have been founded in post-mortem tissue of bipolar disorder patients (72). In drug abusers, neuronal loss, neurodegenerative alterations, a reduction of glial fibrillary acidic protein-immunopositive astrocytes, and widespread axonal damage with concomitant microglial activation as well as reactive and degenerative changes of the cerebral microvasculature have been observed (80, 81). These evidences demonstrate that neuropsychiatric disorders can cause histomorphological damage in particular regions of the brain. Histomorphological damage in the brain will induce individual symptoms or clusters of neuropsychiatric disorders. The location, extent, and degree of brain tissue damage may correlate closely with the type and severity of neuropsychiatric symptoms.

### Brain regions/structures involved in both the category “memory and cognitive deficits” and the category “mental and emotional symptoms”

Both “memory and cognitive deficits” and “mental and emotional symptoms” may involve neuronal damage in several parts of the brain including the limbic system (the hippocampus, the amygdala, the cingulate gyrus, the thalamus, the hypothalamus, the epithalamus, the mammillary body), basal ganglia system (the striatum), the cerebellum, and the cerebral cortex (frontal lobe and temporal lobe) (82, 83). The limbic system is a complex set of brain structures that lie on both sides of the thalamus, right under the cerebrum. It plays a key role in learning, cognition, long-term memory, emotion, motivation, social processing, and behavior. It also influences the endocrine system and the autonomic nervous system. Damage to the limbic system causes many neuropsychiatric disorders including anxiety disorder, bipolar (affective) disorder, psychopathic disorders, amnestic disorders, schizophrenia, and dementias (84, 85). The basal ganglia system is a group of nuclei located near the thalamus and hypothalamus. It consists of the striatum, the globus pallidus, the substantia nigra, the nucleus accumbens, and the subthalamic nucleus (86). It is strongly connected with the cerebral cortex, thalamus, and other brain areas to act as a cohesive functional unit by receiving input from the cerebral cortex and by sending outputs to the motor centers in the brain stem. The basal ganglia system is associated with a variety of functions, including reward learning involving dopamine release and transmission, cognitive and emotional functions, posture and movement, eye movements, and executive functions. Basal ganglia dysfunction has been implicated in a number of neuropsychiatric disorders such as Parkinson’s disease, HD, schizophrenia, Tourette syndrome, hemiballismus, and obsessive–compulsive disorder (87, 88). The cerebellum is a region that is located at the bottom of the brain underneath the cerebral hemispheres. It plays an important role in motor coordination, balance, posture, sensory perception, cognitive functions such as attention and language, and regulation of fear and pleasure responses (89). Cerebellar damage produces disorders related to fine movement and motor coordination (90). The cerebral cortex is the outermost layered structure covering the brain. It is often referred to as gray matter because it consists of cell bodies and capillaries. The cerebral cortex is the largest part of the human brain, associated with higher cortical functions such as cognition, memory, learning, attention, thought, language, information processing, abstraction, creativity, judgment, emotion, consciousness, voluntary muscle activity, somatic sensation, visual stimuli, and movement planning. Almost all neuropsychiatric disorders involve different degrees of damage that occur in the cerebral cortex (91, 92).

### Brain regions/structures involved in the category “consciousness and sleep disturbances”

Consciousness and sleep disturbances may be the result of neuronal damage in the brainstem reticular formation, basal forebrain, the limbic system (hypothalamus and thalamus), and the cerebral cortex (frontal lobe). The reticular formation is a region in the brainstem that has projections to the thalamus and cerebral cortex. It plays a central role in states of consciousness like alertness and sleep by regulating the sleep–wake cycle and filtering incoming stimuli to discriminate irrelevant background stimuli (93, 94). Injury to the brainstem reticular formation can result in coma, consciousness loss, and insomnia (95, 96). The basal forebrain is a group of structures located rostrally and ventrally to the striatum. It is considered to be the major cholinergic output of the brain, and can regulate wakefulness and REM sleep by the induction of ACh release. Damage to the basal forebrain can reduce the amount of acetylcholine in the brain and lead to impaired learning, amnesia, sleep disorder, and lethargy (97, 98). Other brain structures such as the hypothalamus and thalamus of the limbic system and frontal lobe of the cerebral cortex may also involve coordinated regulation of sleep–wake function and conscious states with the reticular formation and basal forebrain (99, 100).

### Brain regions/structures involved in the category “somatoform disorders”

Somatoform disorders are a group of neuropsychiatric disorders characterized by physical symptoms that suggest physical illness or injury. However, the cause for the symptoms cannot be fully explained by a general physical illness because physical examinations and clinical lab tests do not indicate the presence of a physical illness (101). This has led to the hypothesis that the physical symptoms of these patients’ experience may be from a neuropsychiatric source associated with brain damage (102). Somatoform symptoms should not be the result of conscious malingering or factitious behavior. They may be relevant to the damage to some parts of the cerebral cortex (such as dorsolateral prefrontal, insular, rostral anterior cingulate, premotor, parietal cortices, and parahippocampal gyrus) (103, 104). Because the cerebral cortex controls sensory–motor coupling, sensory feedback system, and somatic sensation, cortical damage may cause increased sensitivity to internal physical sensations and pain. The increased sensitivity to somatic feelings may predispose patients to produce somatoform symptoms (105, 106).

### Brain regions/structures involved in the category “impaired psychomotor and neuromotor functions”

Impaired psychomotor and neuromotor functions are defined as abnormalities of motor function that are associated with neuropsychiatric and neurological disorders. Psychomotor impairment (also known as psychomotor retardation) involves a visible slowing of movement (difficulty completing simple tasks such as showering, getting out of bed, or lifting relatively lightweight objects), a general reduction in the speed of thought (such as unable to perform basic math calculation, to find directions on a map, or to plan daily schedule), and difficulty in speaking (such as aphasia) (107, 108). Psychomotor impairment is most-commonly observed in patients with depression and bipolar disorder (109). Neuromotor impairment is a disorder caused by histomorphological, biochemical, or molecular biological abnormalities in the brain. Brain damage is the major contributor to the abnormalities, which can result in a range of neuromotor dysfunction symptoms including poor motor coordination, involuntary movements, difficulty with balance, gait, and mobility control problems, seizures, tremors, spasms, muscle weakness and atrophy, reduced muscle tone, and paralysis (110, 111). Impaired psychomotor and neuromotor functions may result from neuronal damage in the cerebral cortex (the posterior parietal, the primary motor, the premotor, and the supplementary motor cortices), the cerebellum, and the basal ganglia system (112–114), because the three regions of the brain are involved in motor coordination, balance, voluntary muscle activity, movement planning, posture and movement, and sensory perception.

## Potential Mechanisms Involved in Neuropsychiatric Disorders Following Brain Insults

### Secondary neuronal damage

Unlike other tissues or organs, the brain is a very soft, tofu-like tissue. Although it is protected by the skull and cerebrospinal fluid and isolated from large or hydrophilic molecules (bacteria and high-molecular-weight toxins) in the blood stream by the BBB, the brain is extremely susceptible to injury and disease (115). An insult (injury or disease) to the brain may cause more serious consequences than to other tissues. Much of the damage done to the brain does not typically occur at the time of initial insult and does not result directly from the initial insult itself. The progressive deterioration in the brain functions following the initial brain insult results mainly from secondary neuronal damage, a cascade of progressive neural injury and neuronal cell death that is triggered by the initial insult. Secondary neuronal damage usually occurs in both the initial injured site and other brain regions remote from the initial injured site (such as the hippocampus, amygdale, thalamus, the brainstem, basal ganglia system, the cerebellum, and piriform cortex), and possibly continues in the hours, days, weeks, or months following the initial insult. It is largely responsible for the ultimate neuronal cell death and neural loss in the injured brain, and is a major contributor to subsequent neuropsychiatric impairments (19).

**Figure 3:**
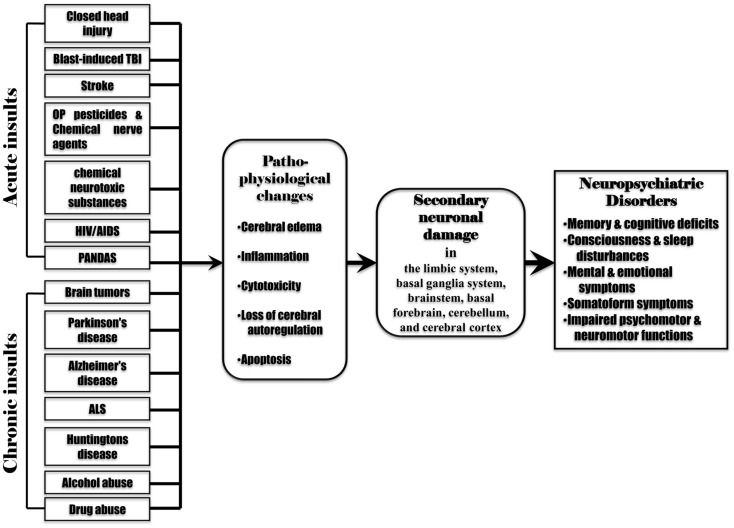
**Various acute and chronic brain insults cause post-injury pathophysiological changes including cerebral edema, inflammation, cytotoxicity, loss of cerebral blood flow autoregulation, and apoptosis in the brain**. These pathophysiological changes induce secondary neuronal damage in the brain regions remote from the initial injured site (the limbic system, basal ganglia system, brainstem, basal forebrain, cerebellum, and cerebral cortex), leading to subsequent neuropsychiatric disorders.

Secondary neuronal damage can be induced by the pathophysiological changes associated with the initial insults such as cerebral edema, inflammation, cytotoxicity, cerebral ischemia, reduced cerebral blood flow, cerebral hypoxia, hypercapnia, acidosis, altered neurotransmitter release, and raised intracranial pressure. Acute brain insults (such as TBI, stroke, poisoning with OP pesticides, chemical nerve agents or chemical neurotoxic substances, and infections by pathogenic microbes) can cause almost all pathophysiological changes as mentioned above. However, chronic brain insults (such as brain tumors, alcohol abuse, drug abuse, and neurodegenerative diseases) may induce only some of these pathophysiological changes. For example, a brain tumor leads to raised intracranial pressure, cerebral edema, inflammation, cerebral hypoxia, cytotoxicity, and altered neurotransmitter release. Neurodegenerative diseases, alcohol abuse, and drug abuse induce altered neurotransmitter release, cytotoxicity, cerebral edema, and inflammation. This may suggest that secondary neuronal damage can occur from the pathophysiological changes following various acute or chronic brain insults. The involvement of secondary neuronal damage will exacerbate neuronal injury and neurologic deficits, ultimately causing irreversible neuropsychiatric and neurological impairments (Figure 3).

### Pathophysiological changes associated with secondary neuronal damage

Cerebral edema, inflammation, cytotoxicity, loss of cerebral blood flow autoregulation, and the apoptosis program are thought to be the major contributors to secondary neuronal damage.

#### Cerebral edema

Cerebral edema is a condition characterized by an excess accumulation of water in the intracellular or extracellular spaces of the brain, causing raised intracranial pressure (116). It can be induced by all acute or chronic brain insults. Cerebral edema is closely associated with the development of secondary neuronal damage, and may be an early marker for progressive neural injury and neuronal cell death. The BBB disruption is the main cause of vasogenic cerebral edema, which is often caused by arterial hypertension, trauma, and tumor-induced release of vasoactive and endothelial substances. The BBB disruption allows intravascular proteins and water to enter and accumulate in the parenchymal extracellular space. Water in white matter can spread rapidly along fiber tracts to the gray matter and other remote regions of the brain, causing widespread cerebral swelling (117). Vasogenic cerebral edema is generally observed in acute brain insults (such as TBI, stroke, chemical poisoning, and infections by pathogenic microbes). Cytotoxic cerebral edema involves the intracellular accumulation of excess water, usually because of dysfunctioning sodium and potassium pumps in the neuronal cell membrane (118). It is often caused by chemical toxins, immune response to microbial infection, alcohol, illegal drugs, and damaged cells from trauma or neurodegenerative diseases. Cerebral edema causes many symptoms including headache, faintness, nausea, vomiting, and blurred vision. In some severe cases, it can lead to seizures and coma.

#### Inflammation

Inflammation is an immune response to harmful stimuli such as injury, microbial invasion, damaged cells, or foreign materials. Inflammation is a defense mechanism that protects the human body from infection and injury, and initiates the tissue-healing process. It leads to increased permeability of the vessels and an increased number of plasma proteins and leukocytes immigrating through vessel walls to the injured tissues (119). Activation of leukocytes results in the expression and release of classical inflammatory mediators including cytokines, eicosanoids, and complement, thus stimulating the signaling networks that regulate inflammatory responses to harmful stimuli (120). During the inflammatory process, destruction and healing of the tissue can happen simultaneously. If the inflammatory response is excessive in the injured tissue, tissue destruction induced by leukocytes will compromise the survival of the injured tissues or organs, causing more severe insults than the initial harmful stimuli. Inflammatory responses exist in almost all acute and chronic brain insults. In contrast to other tissues, neuroinflammatory response in the brain is induced and spread more rapidly because cellular infiltration in the brain in response to inflammation is weaker and delayed (121). When the brain is injured by the initial insult, it will activate microglial cells (the resident immune cells of the brain) to produce pro-inflammatory cytokines (such as interleukins, interferons, and growth factors), leading to a prolonged or exaggerated neuroinflammatory response in the injured areas of the brain (122). The exaggerated neuroinflammatory response is critical in the induction of progressive neural injury and neuronal cell death, which causes more severe tissue destruction and neuronal cell injury in the brain. Neuroinflammation has been recognized to be involved in the development of subsequent neuropsychiatric disorders following brain insults (123).

#### Cytotoxicity induced by neurotransmitter imbalance

Imbalances in some neurotransmitters (such as dopamine, 5-HT, ACh, and glutamate) in the brain can cause stimulation or inhibition of cell membrane receptors, leading to cytotoxicity. Cytotoxicity induces a variety of harmful effects in neurons, including altered calcium influx, free radical damage, oxidative stress, inflammatory responses, and apoptosis, potentially leading to neuronal cell death and neurodegeneration (124). Cytotoxicity involves the development of secondary neuronal damage triggered by many types of brain insult such as TBI, stroke, brain tumors, neurodegenerative diseases (Parkinson’s disease, Alzheimer’s disease, ALS, HD), and poisoning with hazardous chemicals (OP pesticides/chemical nerve agents, chemical neurotoxic substances, alcohol abuse, and drug abuse). Poisoning with hazardous chemicals induces over-release of some neurotransmitters (ACh, dopamine, 5-HT, and/or glutamate) from neurons to produce prolonged stimulation of post-synaptic neuron receptors, triggering excessive calcium release and increased calcium influx in neuronal cells (125). Increased calcium influx activates lipases, proteases, kinases, phosphatases, and endonucleases in potentially harmful metabolic cascades, thus arresting protein synthesis and depriving cells of enzymes or trophic factors essential to their survival (126). In addition, intracellular calcium overload can result in free radical-related damage and apoptosis by inducing excessive oxidative stress and inflammatory responses. Altered calcium influx can cause excitotoxic lesions in the affected neurons. Decreased release of neurotransmitters contributes to some neurodegenerative diseases such as Parkinson’s disease and Alzheimer’s disease. Greatly decreased activity of dopamine-secreting cells caused by progressive neuron death in the basal ganglia is the major cause of Parkinson’s disease (127). Reduced synthesis and activity of ACh in the cholinergic neurons may involve the onset of Alzheimer’s disease (128). Abnormal and decreased levels of glutamate transporters have been observed in ALS and Alzheimer’s disease. Inhibition of glutamate transporter causes elevated glutamate levels and alterations in glutamate receptors, ultimately resulting in excitotoxic lesion in neuronal cells (129).

#### Loss of cerebral blood flow autoregulation

Cerebral blood flow autoregulation is a process that maintains adequate and stable cerebral blood flow and blood pressure to the brain, and avoids underperfusion or overperfusion of the brain. It plays an important role in both delivering sufficient blood-containing oxygen and nutrients to the brain tissue for cerebral metabolic need, and removing CO_2_ and other waste products from the brain. The autoregulation of cerebral blood flow is achieved primarily by dilation or contraction of cerebral arterioles under the influence of multiple complex physiological control systems (including carbon dioxide levels, cerebral metabolic rate, neural activation, activity of the sympathetic nervous system, posture, and other physiological variables) (130). Loss of the ability to autoregulate and control cerebral blood flow in cerebral arterioles occurs often in acute brain insults (especially in TBI and stroke), which results in massive cerebral edema, ischemia, and hypoxia. Impairment of cerebral blood flow autoregulation has been implicated in the development of secondary neuronal damage (131).

#### Apoptosis

In normal conditions, apoptosis generally confers benefits for the human body during the life cycle. Apoptosis can help eliminate old or dead cells by producing cell fragments (called apoptotic bodies). Apoptotic bodies are easily engulfed and quickly removed by phagocytic cells before they spill out onto surrounding cells and cause damage (132). Under the condition that a brain insult occurs, apoptosis becomes a programed cell death process that is fundamental to immune responses and is detrimental to tissue, which exacerbates brain damage and neurologic deficits (133). The apoptosis program can be triggered by excessive free radical formation, death-receptor (such as TNF and Fas receptors) ligation, DNA damage, loss of mitochondrial function, and lysosomal protease activation. Excessive apoptosis will result in uncontrolled cell proliferation, differentiation, and transformation, which plays a crucial role in triggering neuronal cell death (134, 135). Apoptosis, characterized by blebbing, cell shrinkage, nuclear fragmentation, chromatin condensation, and chromosomal DNA fragmentation, is a prominent feature in the brain after an insult (136). Apoptotic processes have been implicated in almost all acute and chronic brain insults, and may be the major contributor to ultimate neuronal cell death/loss in many brain regions remote from the initial injured site (e.g., the hippocampus, amygdale, thalamus, the brainstem, basal ganglia system, the cerebellum, and piriform cortex) (137, 138).

The pathophysiological changes associated with the initial brain insults trigger secondary neuronal damage in the brain. Secondary neuronal damage is the delayed and progressive neuronal cell death, neural loss, and axonal degeneration processes, which occur in not only the initial injured site but also other brain regions remote from the initial injured site. The mechanism by which secondary neuronal damage can happen in other brain regions remote from the initial injured site may involve multiple pathophysiological reactions that occur globally throughout the brain, including widespread cerebral edema and neuroinflammative responses, loss of cerebral blood flow autoregulation, imbalances in neurotransmitters, defective neuronal signal transduction, altered ionic balance, and apoptosis. Secondary neuronal damage is the fundamental cause of subsequent neuropsychiatric and neurological disorders following brain insult. Neuropsychiatric disorders could be the long-term prominent clinical manifestations of the patients with brain insults, because chronic disability following brain damage is mainly expressed as mental sequelae rather than neurological deficits in humans (139). These chronic neuropsychiatric disorders are serious public health problems that result in the loss of many years of productive life and incur large healthcare costs.

## Conclusion

Neuropsychiatric disorders are commonly induced by both acute (TBI, stroke, chemical poisoning, and infections by pathogenic microbes) and chronic (brain tumors, alcohol abuse, drug abuse, and neurodegenerative diseases) brain insults. Clinical manifestations of neuropsychiatric disorders may vary among the patients with different types of brain insult, depending on the cause, type, and severity of injury, acute or chronic condition, and the brain regions affected, as well as on the age and health status of the patient. However, 40% of neuropsychiatric signs and symptoms were found to be commonly shared by traumatic, infectious, toxic, oncogenic, and degenerative brain insults, suggesting that the same or similar brain regions/structures can be possibly affected by different acute and chronic brain insults.

The histomorphological basis for neuropsychiatric disorders might be the progressive neuronal cell death and neural injury in the brain regions that control memory, consciousness, sleep, and cognitive, emotional, somatic, psychomotor, and neuromotor functions, such as the limbic system, basal ganglia system, brainstem, basal forebrain, cerebellum, and cerebral cortex. Because most of the brain regions affected are far away from the initial injured site, the histomorphological alterations in the remote brain regions may mainly result from secondary neuronal damage triggered by the initial insult. Secondary neuronal damage may be largely responsible for subsequent neuropsychiatric disorders following various brain insults. However, it should be noted that clinical manifestations of neurological disorders may tend to be similar even when the histomorphological alterations in the brain region affected are dissimilar, because the brain has only a few common pathophysiological responses (such as cerebral edema, inflammation, cytotoxicity, loss of cerebral blood flow autoregulation, and the apoptos) to injuries.

If a patient has neuropsychiatric signs and symptoms of unknown origin, physicians and psychiatrists should be aware that potential neuronal damage can possibly exist in the patient’s brain and not simply conclude that the patient has a psychological problem associated with social/environmental factors. Necessary treatment aimed at slowing down the process of neuronal damage and attenuating individual symptoms or clusters of neuropsychiatric and neurological disorders may help improve recovery of neurobehavioral functions. The clinical symptom-comparing approach used in the present study to identify the same clinical manifestations may be a useful tool for investigating the causes of neuropsychiatric disorders and other complex diseases of unknown origin.

## Conflict of Interest Statement

The authors declare that the research was conducted in the absence of any commercial or financial relationships that could be construed as a potential conflict of interest.

## Supplementary Material

The Supplementary Material for this article can be found online at http://www.frontiersin.org/Journal/10.3389/fneur.2014.00022/abstract

## References

[1] Hyman SE Chisholm D Kessler R Patel V Whiteford H Mental disorders. 2nd ed In: Jamison DT Breman JG Measham AR Alleyne G Claeson M Evans DB Jha P Mills A Musgrove P, editors. Disease Control Priorities in Developing Countries. Washington, DC: World Bank (2006). p. 605–26

[2] The World Health Organization Cross-national comparisons of the prevalences and correlates of mental disorders. WHO International Consortium in Psychiatric Epidemiology. Bull World Health Organ (2000) 78:413–26 10885160 PMC2560724

[3] Sherer R Mental health care in the developing world. Psychiatric Times (2002) 19:1–6

[4] Kessler RC Berglund P Demler O Jin R Merikangas KR Walters EE Lifetime prevalence and age-of-onset distributions of DSM-IV disorders in the National Comorbidity Survey Replication. Arch Gen Psychiatry (2005) 62:593–602 10.1001/archpsyc.62.6.617 15939837

[5] The World Health Organization Mental Health New Understanding, New Hope. The World Health Report 2001. (2001). Available from: http://www.who.int/whr/2001/en/

[6] Ostman M Kjellin L Stigma by association: psychological factors in relatives of people with mental illness. Br J Psychiatry (2002) 181:494–8 10.1192/bjp.181.6.494 12456519

[7] Kinderman P A psychological model of mental disorder. Harv Rev Psychiatry (2005) 13:206–17 10.1080/10673220500243349 16126607

[8] Rutter M How the environment affects mental health. Br J Psychiatry (2005) 186:4–6 10.1192/bjp.186.1.4 15630116

[9] Schmidt CW Environmental connections: a deeper look into mental illness. Environ Health Perspect (2007) 115:A404–10 10.1289/ehp.115-a404 17687431 PMC1940091

[10] Ahn WK Proctor CC Flanagan EH Mental health clinicians’ beliefs about the biological, psychological, and environmental bases of mental disorders. Cogn Sci (2009) 33:147–82 10.1111/j.1551-6709.2009.01008.x 20411158 PMC2857376

[11] Blair RJ The neurobiology of psychopathic traits in youths. Nat Rev Neurosci (2013) 14:786–99 10.1038/nrn3577 24105343 PMC4418507

[12] Sledzik PS Bellantoni N Brief communication: bioarcheological and biocultural evidence for the New England vampire folk belief. Am J Phys Anthropol (1994) 94:269–74 10.1002/ajpa.1330940210 8085617

[13] Chen Y Huang W Constantini S Concepts and strategies for clinical management of blast-induced traumatic brain injury and posttraumatic stress disorder. J Neuropsychiatry Clin Neurosci (2013) 25:103–10 10.1176/appi.neuropsych.12030058 23686026

[14] Chen Y Constantini S Trembovler V Weinstock M Shohami E An experimental model of closed head injury in mice: pathophysiology, histopathology, and cognitive deficits. J Neurotrauma (1996) 13:557–68 8915907 10.1089/neu.1996.13.557

[15] Chen Y Huang W Constantini S The differences between blast-induced and sports-related brain injuries. Front Neurol (2013) 4:119 10.3389/fneur.2013.00119 23966976 PMC3743039

[16] Chen Y Huang W Non-impact, blast-induced mild TBI and PTSD: concepts and caveats. Brain Inj (2011) 25:641–50 10.3109/02699052.2011.580313 21604927

[17] Donnan GA Fisher M MacLeod M Davis SM Stroke. Lancet (2008) 371:1612–23 10.1016/S0140-6736(08)60694-7 18468545

[18] Sims NR Muyderman H Mitochondria, oxidative metabolism and cell death in stroke. Biochim Biophys Acta (2010) 1802:80–91 10.1016/j.bbadis.2009.09.003 19751827

[19] Chen Y Organophosphate-induced brain damage: mechanisms, neuropsychiatric and neurological consequences, and potential therapeutic strategies. Neurotoxicology (2012) 33:391–400 10.1016/j.neuro.2012.03.011 22498093

[20] Eckert WG Mass deaths by gas or chemical poisoning. A historical perspective. Am J Forensic Med Pathol (1991) 12:119–25 10.1097/00000433-199106000-00007 1882776

[21] Cullen J Avoiding chemical poisoning. Nurs N Z (1996) 2:20–1 8945289

[22] Schaumburg HH Spencer PS Recognizing neurotoxic disease. Neurology (1987) 37:276–8 10.1212/WNL.37.2.276 3808308

[23] Tilson HA Kodavanti PR Mundy WR Bushnell PJ Neurotoxicity of environmental chemicals and their mechanism of action. Toxicol Lett (1998) 10(2-103):631–5 10.1016/S0378-4274(98)00271-9 10022326

[24] Morimoto K Hooper DC Bornhorst A Corisdeo S Bette M Fu ZF Intrinsic responses to Borna disease virus infection of the central nervous system. Proc Natl Acad Sci U S A (1996) 93:13345–50 10.1073/pnas.93.23.13345 8917593 PMC24095

[25] Reinvang I Frøland SS Skripeland V Prevalence of neuropsychological deficit in HIV infection. Incipient signs of AIDS dementia complex in patients with AIDS. Acta Neurol Scand (1991) 83:289–93 10.1111/j.1600-0404.1991.tb04703.x 2063651

[26] Merrill JE Chen IS HIV-1, macrophages, glial cells, and cytokines in AIDS nervous system disease. FASEB J (1991) 5:2391–7 2065887 10.1096/fasebj.5.10.2065887

[27] Pulliam L Clarke JA McGuire D McGrath MS Investigation of HIV-infected macrophage neurotoxin production from patients with AIDS dementia. Adv Neuroimmunol (1994) 4:195–8 10.1016/S0960-5428(06)80257-3 7874387

[28] Moretti G Pasquini M Mandarelli G Tarsitani L Biondi M What every psychiatrist should know about PANDAS: a review. Clin Pract Epidemiol Ment Health (2008) 4:13 10.1186/1745-0179-4-13 18495013 PMC2413218

[29] Martino D Defazio G Giovannoni G The PANDAS subgroup of tic disorders and childhood-onset obsessive-compulsive disorder. J Psychosom Res (2009) 67:547–57 10.1016/j.jpsychores.2009.07.004 19913659

[30] de Oliveira SK Pelajo CF Pediatric autoimmune neuropsychiatric disorders associated with streptococcal infection (PANDAS): a controversial diagnosis. Curr Infect Dis Rep (2010) 12:103–9 10.1007/s11908-010-0082-7 21308506

[31] Snyder H Robinson K Shah D Brennan R Handrigan M Signs and symptoms of patients with brain tumors presenting to the emergency department. J Emerg Med (1993) 11:253–8 10.1016/0736-4679(93)90042-6 8340578

[32] Constantini S Tamir J Gomori MJ Shohami E Tumor prostaglandin levels correlate with edema around meningiomas. Neurosurgery (1993) 33:204–11 10.1097/00006123-199308000-00004 8367041

[33] Jarquin-Valdivia AA Psychiatric symptoms and brain tumors: a brief historical overview. Arch Neurol (2004) 61:1800–4 10.1001/archneur.61.11.1800 15534193

[34] Roth J Keating RF Myseros JS Yaun AL Magge SN Constantini S Pediatric incidental brain tumors: a growing treatment dilemma. J Neurosurg Pediatr (2012) 10:168–74 10.3171/2012.6.PEDS11451 22816603

[35] Bellows JG Alcohol abuse and brain damage. Compr Ther (1979) 5:3–5 487730

[36] Ward RJ Lallemand F de Witte P Biochemical and neurotransmitter changes implicated in alcohol-induced brain damage in chronic or ‘binge drinking’ alcohol abuse. Alcohol Alcohol (2009) 44:128–35 10.1093/alcalc/agn100 19155229

[37] Nail RL Dean LM Drug abuse: a manifestation of the cyclic nature of human behavior. Drug Alcohol Depend (1976) 1:429–34 10.1016/0376-8716(76)90007-7 1017386

[38] Schulden JD Lopez MF Compton WM Clinical implications of drug abuse epidemiology. Psychiatr Clin North Am (2012) 35:411–23 10.1016/j.psc.2012.03.007 22640763 PMC3383008

[39] Substance Abuse and Mental Health Services Administration Results from the 2011 National Survey on Drug Use and Health: Summary of National Findings. NSDUH Series H-44, HHS Publication No. (SMA) 12-4713. Rockville, MD (2012). Available from: http://www.samhsa.gov/data/nsduh/2k11results/nsduhresults2011.htm#Ch2

[40] Gordon HW Early environmental stress and biological vulnerability to drug abuse. Psychoneuroendocrinology (2002) 27:115–26 10.1016/S0306-4530(01)00039-7 11750773

[41] Miczek KA Yap JJ Covington HE III Social stress, therapeutics and drug abuse: preclinical models of escalated and depressed intake. Pharmacol Ther (2006) 120:102–28 10.1016/j.pharmthera.2008.07.006 18789966 PMC2713609

[42] De La Torre R Farré M Roset PN Pizarro N Abanades S Segura M Human pharmacology of MDMA: pharmacokinetics, metabolism, and disposition. Ther Drug Monit (2004) 26:137–44 10.1097/00007691-200404000-00009 15228154

[43] Bolla KI McCann UD Ricaurte GA Memory impairment in abstinent MDMA (“Ecstasy”) users. Neurology (1998) 51:1532–7 10.1212/WNL.51.6.1532 9855498

[44] Fattore L Piras G Corda MG Giorgi O The Roman high- and low-avoidance rat lines differ in the acquisition, maintenance, extinction, and reinstatement of intravenous cocaine self-administration. Neuropsychopharmacology (2009) 34:1091–101 10.1038/npp.2008.43 18418365

[45] Bolla KI Rothman R Cadet JL Dose-related neurobehavioral effects of chronic cocaine use. J Neuropsychiatry Clin Neurosci (1999) 11:361–9 10440013 10.1176/jnp.11.3.361

[46] Bolla KI Funderburk FR Cadet JL Differential effects of cocaine and cocaine alcohol on neurocognitive performance. Neurology (2000) 54:2285–92 10.1212/WNL.54.12.2285 10881254

[47] Planeta CS Lepsch LB Alves R Scavone C Influence of the dopaminergic system, CREB, and transcription factor-κB on cocaine neurotoxicity. Braz J Med Biol Res (2013) 46:909–15 10.1590/1414-431X20133379 24141554 PMC3854330

[48] Malenka RC Nestler EJ Hyman SE Unlike cocaine and amphetamine, methamphetamine is directly toxic to midbrain dopamine neurons. 2nd ed In: Sydor A Brown RY editors. Molecular Neuropharmacology: A Foundation for Clinical Neuroscience. New York: McGraw-Hill (2009). 370 p.

[49] Darke S Kaye S McKetin R Duflou J Major physical and psychological harms of methamphetamine use. Drug Alcohol Rev (2008) 27:253–62 10.1080/09595230801923702 18368606

[50] Bolla KI Eldreth DA Matochik JA Cadet JL Neural substrates of faulty decision-making in abstinent marijuana users. Neuroimage (2005) 26:480–92 10.1016/j.neuroimage.2005.02.012 15907305

[51] Bolla KI Brown K Eldreth D Tate K Cadet JL Dose-related neurocognitive effects of marijuana use. Neurology (2002) 59:1337–43 10.1212/01.WNL.0000031422.66442.49 12427880

[52] Richards M Stern Y Marder K Cote L Mayeux R Relationships between extrapyramidal signs and cognitive function in a community-dwelling cohort of patients with Parkinson’s disease and normal elderly individuals. Ann Neurol (1993) 33:267–74 10.1002/ana.410330307 8498810

[53] Stern Y Richards M Sano M Mayeux R Comparison of cognitive changes in patients with Alzheimer’s and Parkinson’s disease. Arch Neurol (1993) 50:1040–5 10.1001/archneur.1993.00540100035011 8215961

[54] Samii A Nutt JG Ransom BR Parkinson’s disease. Lancet (2004) 363:1783–93 10.1016/S0140-6736(04)16305-8 15172778

[55] Jankovic J Parkinson’s disease: clinical features and diagnosis. J Neurol Neurosurg Psychiatry (2008) 79:368–76 10.1136/jnnp.2007.131045 18344392

[56] Busser J Geldmacher DS Herrup K Ectopic cell cycle proteins predict the sites of neuronal cell death in Alzheimer’s disease brain. J Neurosci (1998) 18:2801–7 9525997 10.1523/JNEUROSCI.18-08-02801.1998 PMC6792587

[57] Gold CA Budson AE Memory loss in Alzheimer’s disease: implications for development of therapeutics. Expert Rev Neurother (2008) 8:1879–91 10.1586/14737175.8.12.1879 19086882 PMC2655107

[58] Tiraboschi P Hansen LA Thal LJ Corey-Bloom J The importance of neuritic plaques and tangles to the development and evolution of AD. Neurology (2004) 62:1984–9 10.1212/01.WNL.0000129697.01779.0A 15184601

[59] Phukan J Pender NP Hardiman O Cognitive impairment in amyotrophic lateral sclerosis. Lancet Neurol (2007) 6:994–1003 10.1016/S1474-4422(07)70265-X 17945153

[60] Chio A Calvo A Moglia C Mazzini L Mora G Parals Study Group Phenotypic heterogeneity of amyotrophic lateral sclerosis: a population based study. J Neurol Neurosurg Psychiatry (2011) 82:740–6 10.1136/jnnp.2010.235952 21402743

[61] Young AB Role of excitotoxins in heredito-degenerative neurologic diseases. Res Publ Assoc Res Nerv Ment Dis (1993) 71:175–89 8093331

[62] Young AB Impairment of energy metabolism and excitotoxic cell death in Huntington disease. Rev Neurol (Paris) (1997) 153:496–8 9683998

[63] Walker FO Huntington’s disease. Lancet (2007) 369:218–28 10.1016/S0140-6736(07)60111-1 17240289

[64] Berent S Giordani B Lehtinen S Markel D Penney JB Buchtel HA Positron emission tomographic scan investigations of Huntington’s disease: cerebral metabolic correlates of cognitive function. Ann Neurol (1988) 23:541–6 10.1002/ana.410230603 2970247

[65] Montoya A Price BH Menear M Lepage M Brain imaging and cognitive dysfunctions in Huntington’s disease. J Psychiatry Neurosci (2006) 31:21–9 16496032 PMC1325063

[66] Paulsen JS Smith MM Long JD PREDICT HD investigators, Coordinators of the Huntington Study Group Cognitive decline in prodromal Huntington disease: implications for clinical trials. J Neurol Neurosurg Psychiatry (2013) 84:1233–9 10.1136/jnnp-2013-305114 23911948 PMC3795884

[67] Neyeloff JL Fuchs SC Moreira LB Meta-analyses and Forest plots using a Microsoft excel spreadsheet: step-by-step guide focusing on descriptive data analysis. BMC Res Notes (2012) 5:52 10.1186/1756-0500-5-52 22264277 PMC3296675

[68] Kronish IM Edmondson D Goldfinger JZ Fei K Horowitz CR Posttraumatic stress disorder and adherence to medications in survivors of strokes and transient ischemic attacks. Stroke (2012) 43:2192–7 10.1161/STROKEAHA.112.655209 22618380 PMC3404197

[69] Bruce M Gumley D Isham L Fearon P Phipps K Post-traumatic stress symptoms in childhood brain tumour survivors and their parents. Child Care Health Dev (2011) 37:244–51 10.1111/j.1365-2214.2010.01164.x 21083688

[70] Kawana N Ishimatsu S Kanda K Psycho-physiological effects of the terrorist sarin attack on the Tokyo subway system. Mil Med (2001) 166:23–6 11778423

[71] Yoshihara Y Sugihara G Matsumoto H Suckling J Nishimura K Toyoda T Voxel-based structural magnetic resonance imaging (MRI) study of patients with early onset schizophrenia. Ann Gen Psychiatry (2008) 7:25 10.1186/1744-859X-7-25 19102744 PMC2628340

[72] Matthews PR Harrison PJ A morphometric, immunohistochemical, and in situ hybridization study of the dorsal raphe nucleus in major depression, bipolar disorder, schizophrenia, and suicide. J Affect Disord (2012) 137:125–34 10.1016/j.jad.2011.10.043 22129767 PMC3314923

[73] Holleran L Ahmed M Anderson-Schmidt H McFarland J Emsell L Leemans A Altered interhemispheric and temporal lobe white matter microstructural organization in severe chronic schizophrenia. Neuropsychopharmacology (2013) 39:944–54 10.1038/npp.2013.294 24150571 PMC3924528

[74] Takei Y Suda M Aoyama Y Yamaguchi M Sakurai N Narita K Temporal lobe and inferior frontal gyrus dysfunction in patients with schizophrenia during face-to-face conversation: a near-infrared spectroscopy study. J Psychiatr Res (2013) 47:1581–9 10.1016/j.jpsychires.2013.07.029 23978395

[75] Lim HK Jung WS Aizenstein HJ Aberrant topographical organization in gray matter structural network in late life depression: a graph theoretical analysis. Int Psychogeriatr (2013) 25:1929–40 10.1017/S104161021300149X 24093725

[76] Willard SL Riddle DR Forbes ME Shively CA Cell number and neuropil alterations in subregions of the anterior hippocampus in a female monkey model of depression. Biol Psychiatry (2013) 74:890–7 10.1016/j.biopsych.2013.03.013 23607969 PMC3732810

[77] Kempton MJ Geddes JR Ettinger U Williams SC Grasby PM Meta-analysis, database, and meta-regression of 98 structural imaging studies in bipolar disorder. Arch Gen Psychiatry (2008) 65:1017–32 10.1001/archpsyc.65.9.1017 18762588

[78] Arnone D Cavanagh J Gerber D Lawrie SM Ebmeier KP McIntosh AM Magnetic resonance imaging studies in bipolar disorder and schizophrenia: meta-analysis. Br J Psychiatry. (2009) 195:194–201 10.1192/bjp.bp.108.059717 19721106

[79] Strakowski SM Adler CM Almeida J Altshuler LL Blumberg HP Chang KD The functional neuroanatomy of bipolar disorder: a consensus model. Bipolar Disord (2012) 14:313–25 10.1111/j.1399-5618.2012.01022.x 22631617 PMC3874804

[80] Büttner A Kroehling C Mall G Penning R Weis S Alterations of the vascular basal lamina in the cerebral cortex in drug abuse: a combined morphometric and immunohistochemical investigation. Drug Alcohol Depend (2005) 79:63–70 10.1016/j.drugalcdep.2004.12.010 15943945

[81] Büttner A Review: the neuropathology of drug abuse. Neuropathol Appl Neurobiol (2011) 37:118–34 10.1111/j.1365-2990.2010.01131.x 20946118

[82] Vertes RP Interactions among the medial prefrontal cortex, hippocampus and midline thalamus in emotional and cognitive processing in the rat. Neuroscience (2006) 142:1–20 10.1016/j.neuroscience.2006.06.027 16887277

[83] Grossberg S Adaptive Resonance Theory: how a brain learns to consciously attend, learn, and recognize a changing world. Neural Netw (2013) 37:1–47 10.1016/j.neunet.2012.09.017 23149242

[84] Satterthwaite TD Wolf DH Loughead J Ruparel K Valdez JN Siegel SJ Association of enhanced limbic response to threat with decreased cortical facial recognition memory response in schizophrenia. Am J Psychiatry (2010) 167:418–26 10.1176/appi.ajp.2009.09060808 20194482 PMC4243460

[85] Passarotti AM Sweeney JA Pavuluri MN Fronto-limbic dysfunction in mania pre-treatment and persistent amygdala over-activity post-treatment in pediatric bipolar disorder. Psychopharmacology (Berl) (2011) 216:485–99 10.1007/s00213-011-2243-2 21390505 PMC3174733

[86] Fix JD Basal ganglia and the striatal motor system. In: Wilkins WKLW, editor. Neuroanatomy (Board Review Series). 4th ed Baltimore: Wolters Kluwer & Lippincott Williams & Wilkins (2008). p. 274–81

[87] van der Stelt M Di Marzo V The endocannabinoid system in the basal ganglia and in the mesolimbic reward system: implications for neurological and psychiatric disorders. Eur J Pharmacol (2003) 480:133–50 10.1016/j.ejphar.2003.08.101 14623357

[88] Rothwell JC The motor functions of the basal ganglia. J Integr Neurosci (2011) 10:303–15 10.1142/S0219635211002798 21960305

[89] Wolf U Rapoport MJ Schweizer TA Evaluating the affective component of the cerebellar cognitive affective syndrome. J Neuropsychiatry Clin Neurosci (2009) 21:245–53 10.1176/appi.neuropsych.21.3.245 19776302

[90] Fine EJ Ionita CC Lohr L The history of the development of the cerebellar examination. Semin Neurol (2002) 22:375–84 10.1055/s-2002-36759 12539058

[91] Bowen DM Najlerahim A Procter AW Francis PT Murphy E Circumscribed changes of the cerebral cortex in neuropsychiatric disorders of later life. Proc Natl Acad Sci U S A (1989) 86:9504–8 10.1073/pnas.86.23.9504 2574463 PMC298525

[92] de Courten-Myers GM The human cerebral cortex: gender differences in structure and function. J Neuropathol Exp Neurol (1999) 58:217–26 10.1097/00005072-199903000-00001 10197813

[93] Sumitomo I Hayashi Y Electrical responses of cat superior colliculus in reticular formation activation and during natural sleep-wakefulness cycle. Tohoku J Exp Med (1967) 91:13–30 10.1620/tjem.91.13 6050296

[94] Ishizawa Y Ma HC Dohi S Shimonaka H Effects of cholinomimetic injection into the brain stem reticular formation on halothane anesthesia and antinociception in rats. J Pharmacol Exp Ther (2000) 293:845–51 10869384

[95] Hill DL Almli CR Midbrain reticular formation damage and the ontogeny of ingestive and sensorimotor behaviors. Physiol Behav (1981) 26:269–75 10.1016/0031-9384(81)90022-6 7232532

[96] Jordan WP Leaton RN Habituation of the acoustic startle response in rats after lesions in the mesencephalic reticular formation or in the inferior colliculus. Behav Neurosci (1983) 97:710–24 10.1037/0735-7044.97.5.710 6639744

[97] Szymusiak R McGinty D Sleep suppression following kainic acid-induced lesions of the basal forebrain. Exp Neurol (1986) 94:598–614 10.1016/0014-4886(86)90240-2 3780909

[98] Sharma R Engemann SC Sahota P Thakkar MM Effects of ethanol on extracellular levels of adenosine in the basal forebrain: an in vivo microdialysis study in freely behaving rats. Alcohol Clin Exp Res (2010) 34:813–8 10.1111/j.1530-0277.2010.01153.x 20184564 PMC2884072

[99] Neumann MA Eger EI II Weiskopf RB Solubility of volatile anesthetics in bovine white matter, cortical gray matter, thalamus, hippocampus, and hypothalamic area. Anesth Analg (2005) 100:1003–6 10.1213/01.ANE.0000146516.35921.1A 15781514

[100] Jellinger KA Functional pathophysiology of consciousness. Neuropsychiatr (2009) 23:115–33 19573504

[101] Oyama O Paltoo C Greengold J Somatoform disorders. Am Fam Physician (2007) 76:1333–8 18019877

[102] Kreipe RE The biopsychosocial approach to adolescents with somatoform disorders. Adolesc Med Clin (2006) 17:1–24 16473291 10.1016/j.admecli.2005.11.003

[103] Stein DJ Muller J Cognitive-affective neuroscience of somatization disorder and functional somatic syndromes: reconceptualizing the triad of depression anxiety-somatic symptoms. CNS Spectr (2008) 13:379–84 18496475 10.1017/s1092852900016540

[104] García-Campayo J Fayed N Serrano-Blanco A Roca M Brain dysfunction behind functional symptoms: neuroimaging and somatoform, conversive, and dissociative disorders. Curr Opin Psychiatry (2009) 22:224–31 10.1097/YCO.0b013e3283252d43 19553880

[105] Farrugia D Fetter H Chronic pain: biological understanding and treatment suggestions for mental health counselors. J Ment Health Couns (2009) 31:189–200

[106] Katzer A Oberfeld D Hiller W Gerlach AL Witthöft M Tactile perceptual processes and their relationship to somatoform disorders. J Abnorm Psychol (2012) 121:530–43 10.1037/a0026536 22149912

[107] Widlöcher DJ Psychomotor retardation: clinical, theoretical, and psychometric aspects. Psychiatr Clin North Am (1983) 6:27–40 6889173

[108] King DJ Psychomotor impairment and cognitive disturbances induced by neuroleptics. Acta Psychiatr Scand Suppl (1994) 380:53–8 10.1111/j.1600-0447.1994.tb05833.x 7914050

[109] Pier MP Hulstijn W Sabbe BG Psychomotor retardation in elderly depressed patients. J Affect Disord (2004) 81:73–7 10.1016/j.jad.2003.08.002 15183603

[110] Zohar O Rubovitch V Milman A Schreiber S Pick CG Behavioral consequences of minimal traumatic brain injury in mice. Acta Neurobiol Exp (Wars) (2011) 71:36–45 21499325 10.55782/ane-2011-1821

[111] Stevens MC Lovejoy D Kim J Oakes H Kureshi I Witt ST Multiple resting state network functional connectivity abnormalities in mild traumatic brain injury. Brain Imaging Behav (2012) 6:293–318 10.1007/s11682-012-9157-4 22555821

[112] Frackowiak RS Weiller C Chollet F The functional anatomy of recovery from brain injury. Ciba Found Symp (1991) 163:235–44 1815894 10.1002/9780470514184.ch14

[113] Abu-Judeh HH Parker R Singh M el-Zeftawy H Atay S Kumar M SPET brain perfusion imaging in mild traumatic brain injury without loss of consciousness and normal computed tomography. Nucl Med Commun (1999) 20:505–10 10.1097/00006231-199906000-00003 10451861

[114] Slobounov S Sebastianelli W Moss R Alteration of posture-related cortical potentials in mild traumatic brain injury. Neurosci Lett (2005) 383:251–5 10.1016/j.neulet.2005.04.039 15876490

[115] Parent A Carpenter MB Carpenter’s Human Neuroanatomy. 9th ed Philadelphia: Lippincott Williams & Wilkins (1995). p. 186–92

[116] Unterberg AW Stover J Kress B Kiening KL Edema and brain trauma. Neuroscience (2004) 129:1021–9 10.1016/j.neuroscience.2004.06.046 15561417

[117] Bothe HW Bodsch W Hossmann KA Relationship between specific gravity, water content, and serum protein extravasation in various types of vasogenic brain edema. Acta Neuropathol (1984) 64:37–42 10.1007/BF00695604 6475495

[118] Agrawal SK Fehlings MG Mechanisms of secondary injury to spinal cord axons in vitro: role of Na+, Na(+)-K(+)-ATPase, the Na(+)-H+ exchanger, and the Na(+)-Ca2+ exchanger. J Neurosci (1996) 16:545–52 8551338 10.1523/JNEUROSCI.16-02-00545.1996 PMC6578655

[119] Morganti-Kossmann MC Rancan M Stahel PF Kossmann T Inflammatory response in acute traumatic brain injury: a double-edged sword. Curr Opin Crit Care (2002) 8:101–5 10.1097/00075198-200204000-00002 12386508

[120] Allan SM Rothwell NJ Cytokines and acute neurodegeneration. Nat Rev Neurosci (2001) 2:734–44 10.1038/35094583 11584311

[121] Lo EH Dalkara T Moskowitz MA Mechanisms, challenges and opportunities in stroke. Nat Rev Neurosci (2003) 4:399–415 10.1038/nrn1106 12728267

[122] Frank MG Barrientos RM Thompson BM Weber MD Watkins LR Maier SF IL-1RA injected intra-cisterna magna confers extended prophylaxis against lipopolysaccharide-induced neuroinflammatory and sickness responses. J Neuroimmunol (2012) 252:33–9 10.1016/j.jneuroim.2012.07.010 22871632 PMC5652306

[123] Pucak ML Carroll KA Kerr DA Kaplin AI Neuropsychiatric manifestations of depression in multiple sclerosis: neuroinflammatory, neuroendocrine, and neurotrophic mechanisms in the pathogenesis of immune-mediated depression. Dialogues Clin Neurosci (2007) 9:125–39 17726912 10.31887/DCNS.2007.9.2/mpucak PMC3181849

[124] Chen J Wersinger C Sidhu A Chronic stimulation of D1 dopamine receptors in human SK-N-MC neuroblastoma cells induces nitric-oxide synthase activation and cytotoxicity. J Biol Chem (2003) 278:28089–100 10.1074/jbc.M303094200 12738794

[125] Clement JG Broxup B Efficacy of diazepam and avizafone against soman-induced neuropathology in brain of rats. Neurotoxicology (1993) 14:485–504 8164892

[126] Siesjo BK Siesjo P Mechanisms of secondary brain injury. Eur J Anaesthesiol (1996) 13:247–68 10.1097/00003643-199605000-00004 8737117

[127] Nagatsu T Sawada M Biochemistry of postmortem brains in Parkinson’s disease: historical overview and future prospects. J Neural Transm Suppl (2007) 72:113–20 10.1007/978-3-211-73574-9_14 17982884

[128] Gil-Bea FJ García-Alloza M Domínguez J Marcos B Ramírez MJ Evaluation of cholinergic markers in Alzheimer’s disease and in a model of cholinergic deficit. Neurosci Lett (2005) 375:37–41 10.1016/j.neulet.2004.10.062 15664119

[129] Wilson JM Khabazian I Pow DV Craig UK Shaw CA Decrease in glial glutamate transporter variants and excitatory amino acid receptor down-regulation in a murine model of ALS-PDC. Neuromolecular Med (2003) 3:105–18 10.1385/NMM:3:2:105 12728193

[130] Seifert T Secher NH Sympathetic influence on cerebral blood flow and metabolism during exercise in humans. Prog Neurobiol (2011) 95:406–26 10.1016/j.pneurobio.2011.09.008 21963551

[131] Strebel S Lam AM Matta BF Newell DW Impaired cerebral autoregulation after mild brain injury. Surg Neurol (1997) 47:128–31 10.1016/S0090-3019(96)00459-4 9040813

[132] Khan T Waring P Macrophage adherence prevents apoptosis induced by ricin. Eur J Cell Biol (1993) 62:406–14 7925496

[133] Tang DG Porter AT Apoptosis: a current molecular analysis. Pathol Oncol Res (1996) 2:117–31 10.1007/BF02903515 11173595

[134] Takada Y Gillenwater A Ichikawa H Aggarwal BB Suberoylanilide hydroxamic acid potentiates apoptosis, inhibits invasion, and abolishes osteoclastogenesis by suppressing nuclear factor-kappaB activation. J Biol Chem (2006) 281:5612–22 10.1074/jbc.M507213200 16377638

[135] Franzen CA Chen CC Todorovic V Juric V Monzon RI Lau LF Matrix protein CCN1 is critical for prostate carcinoma cell proliferation and TRAIL-induced apoptosis. Mol Cancer Res (2009) 7:1045–55 10.1158/1541-7786.MCR-09-0017 19584265 PMC2712585

[136] Conti AC Raghupathi R Trojanowski JQ McIntosh TK Experimental brain injury induces regionally distinct apoptosis during the acute and delayed post-traumatic period. J Neurosci (1998) 18:5663–72 9671657 10.1523/JNEUROSCI.18-15-05663.1998 PMC6793063

[137] Ng I Yeo TT Tang WY Soong R Ng PY Smith DR Apoptosis occurs after cerebral contusions in humans. Neurosurgery (2000) 46:949–56 10.1227/00006123-200004000-00034 10764270

[138] Lopez-Neblina F Toledo AH Toledo-Pereyra LH Molecular biology of apoptosis in ischemia and reperfusion. J Invest Surg (2005) 18:335–50 10.1080/08941930500328862 16319055

[139] Jennett B Snoek J Bond MR Brooks N Disability after severe head injury: observations in the use of the Glasgow outcome scale. J Neurol Neurosurg Psychiatry (1981) 44:285–93 10.1136/jnnp.44.4.285 6453957 PMC490949

