# Supplementary material for: The Involvement of Secondary Neuronal Damage in the Development of Neuropsychiatric Disorders Following Brain Insults
Source: Front Neurol. 2014 Mar 11;5:22. doi: 10.3389/fneur.2014.00022 (PMC3949352; doi:10.3389/fneur.2014.00022)
Supplement: Supplementary file 1 [file Data_Sheet1.PDF]

Supplementary Table 1. 55 neuropsychiatric signs or symptoms identified for 14 types of acute and chronic brain insults based on the 528 supplementary references

Brain insult			Acute brain insults							Chronic brain insults						
			CHI	Blast-induced TBI	Stroke	OP/chemical nerve agents	Chemical neurotoxic substances	HIV/AIDS	PANDAS	Brain tumor	Alcohol abuse	Drug abuse	PD	AD	ALS	HD (Chorea)
Neuropsychiatric symptoms	Memory & cognitive deficits	Memory loss	[235]	[425]	[119]*	[416]	[243]	[335]	[318]	[244]	[111]	[247]	[197]	[71]	[466]	[445]
		Concentration difficulties	[230]	[69]	[242]	[105]	[384]	[329]	[464]	[331]	[343]	[495]	[47]	[375]	[72]	[151]
		Cognitive impairments	[95]	[44]	[467]	[205]	[508]	[380]	[457]	[217]	[104]	[247]	[454]	[374]	[298]	[159]
		Learning disabilities	[447]	[465]	[326]	[105]	[385]	[26]	[320]	[110]	[46]	[29]	[17]	[229]	[414]	[382]
		Forgetfulness	[459]	[44]	[162]	[105]	[508]	[18]	[256]	[3]	[463]	[332]	[451]	[446]	[471]	[226]
		Impaired attention	[183]	[132]	[2]	[70]	[508]	[337]	[410]	[244]	[275]	[11]	[9]	[328]	[492]	[152]
		Impaired judgment	[292]	[293]	[145]	[70]	[243]	[188]		[244]	[176]	[82]	[323]	[1]	[169]	[84]
		Confusion	[325]	[373]	[460]	[352]	[385]	[380]		[196]	[50]	[131]	[101]	[276]	[6]	[159]
		Disorientation	[433]	[395]	[428]	[352]	[385]	[33]		[331]	[343]	[77]	[192]	[208]	[6]	[84]
		Dementia	[418]	[42]	[437]			[502]	[423]	[331]	[50]	[350]	[191]	[106]	[257]	[486]
	Consciousness & sleep disturbances	Sleep disorder	[324]	[80]	[87]	[472]	[150]	[213]	[92]	[148]	[361]	[455]	[357]	[399]	[261]	[491]
		Insomnia	[524]	[365]	[358]	[472]	[171]	[401]	[283]	[271]	[67]	[75]	[154]	[288]	[21]	[15]
		Consciousness loss	[42]	[52]	[144]	[523]	[417]	[199]		[241]	[16]	[32]		[153]		
		Coma	[237]	[516]	[228]	[166]	[368]	[502]		[388]				[31]		
		Lethargy	[103]	[395]	[421]	[528]	[316]	[109]		[300]						
	Mental & emotional symptoms	Anxiety	[211]	[202]	[497]	[338]	[48]	[213]	[65]	[270]	[8]	[138]	[519]	[35]	[239]	[84]
		Depression	[125]	[125]	[497]	[523]	[336]	[380]	[147]	[503]	[62]	[232]	[66]	[452]	[239]	[234]
		Aggression/Agitation	[426]	[299]	[411]	[431]	[430]	[317]	[406]	[493]	[511]	[313]	[55]	[19]	[334]	[57]
		Moodiness	[209]	[299]	[510]	[416]	[161]	[18]	[92]	[43]	[39]	[138]	[137]	[58]	[304]	[203]
		Psychosis	[287]	[170]	[379]	[301]	[336]	[294]	[64]	[527]	[124]	[155]	[250]	[251]	[257]	[389]
		Increased emotional sensitivity	[285]	[139]	[259]	[212]	[150]	[54]	[464]	[56]	[391]	[475]	[41]	[415]	[264]	[479]
		Fear/Frightened	[158]	[49]	[224]	[442]	[48]	[193]		[156]	[364]	[165]	[362]	[185]	[330]	[377]
		Behavior stress	[24]	[396]	[346]	[521]	[174]	[439]		[219]	[76]	[94]	[181]	[102]	[195]	[509]
		Unusual temper tantrums/Irritability	[522]	[299]	[85]	[431]	[289]			[40]	[204]	[371]	[307]	[438]	[366]	[57]
		Mania	[210]	[478]	[413]		[172]	[128]		[268]	[96]	[458]	[517]	[265]		[339]
		Suicidal thoughts/attempts	[441]	[53]	[412]		[336]	[378]			[488]	[349]	[327]	[394]	[355]	[506]
		Restlessness	[512]	[27]	[59]		[117]		[484]	[268]	[120]		[462]	[108]		[203]
		Hallucinations	[173]	[170]	[310]		[258]			[268]	[505]	[449]	[344]	[443]		[84]
		Apathy	[5]	[485]	[60]			[380]				[20]	[284]	[245]	[168]	[57]
		PTSD	[252]	[28]	[127]	[70]	[133]	[340]		[215]	[291]	[90]				
		Social phobias	[286]	[299]	[290]				[34]	[270]	[282]	[469]	[236]	[351]		
		Paranoia	[231]	[507]				[429]		[274]		[295]	[248]	[135]		[226]
		Panic disorder	[420]				[174]		[277]	[268]	[83]	[281]	[372]			
		Impulsivity	[164]	[500]				[23]	[34]			[404]	[444]	[392]		
		Obsessive-compulsive disorder	[186]						[225]			[123]	[269]		[260]	[360]
		Delusions	[142]		[296]							[25]	[187]	[38]		[226]
	Somatoform symptoms	Headache	[263]	[129]	[489]	[91]	[63]	[329]	[118]	[157]	[450]	[178]	[262]	[182]	[74]	[226]
		Reduced visuo-spatial abilities	[149]	[81]	[279]	[93]	[473]	[514]	[256]	[422]	[115]	[297]	[221]	[201]	[280]	[159]
		Blurred vision	[79]	[254]	[400]	[376]	[477]	[222]	[363]	[116]	[383]	[7]	[367]	[353]	[314]	[246]
		Fatigue	[30]	[220]	[305]	[223]	[381]	[496]	[436]	[14]	[515]	[233]	[122]	[476]	[261]	[226]
		Altered hearing and ringing in the ears	[255]	[126]	[134]	[249]	[387]	[482]	[345]	[354]	[73]	[468]	[494]	[113]	[112]	
		Nausea	[100]	[220]	[12]	[390]	[347]	[501]		[348]	[120]	[160]	[177]	[206]		
		Vomiting	[99]	[220]	[435]	[184]	[88]	[474]		[189]	[432]	[160]	[177]	[206]		
		Dizziness	[68]	[419]	[207]	[342]	[308]	[179]		[272]	[490]		[525]	[10]		
		Tired eyes	[79]	[254]	[504]	[303]	[477]	[222]					[386]		[314]	
		Impaired sense of smell	[136]				[218]			[434]	[403]	[449]	[369]	[499]		
		Pupil abnormalities	[180]		[78]	[45]	[417]	[408]					[302]		[314]	
		Altered sense of taste	[143]		[114]		[393]					[214]	[216]	[453]		
		Hypersexuality	[37]		[311]							[89]	[97]	[470]		
	Impaired psychomotor & neuromotor functions	Psychomotor retardation	[306]	[121]	[98]	[105]	[267]	[337]	[278]	[4]	[483]	[190]	[130]	[227]	[370]	[440]
		Lack of motor coordination	[61]	[498]	[240]	[416]	[238]	[502]	[518]	[354]	[273]	[424]	[481]	[520]	[333]	[309]
		Difficulty balancing	[141]	[498]	[526]	[416]	[461]	[22]	[319]	[354]	[398]	[297]	[359]	[200]	[13]	[405]
		Seizures/tremors	[194]	[407]	[322]	[253]	[321]	[36]	[266]	[480]	[163]	[51]	[312]	[140]	[356]	[402]
		Aphasia (difficulty speaking)	[167]	[407]	[175]			[109]		[513]	[146]	[315]	[409]	[487]	[86]	
		Abnormal facial expression	[107]								[456]		[448]	[427]	[341]	[198]

[#]*: Reference number of the Supplementary References

References for Supplementary Table 1

1. Abe N, Fujii T, Nishio Y, Iizuka O, Kanno S, Kikuchi H, Takagi M, Hiraoka K, Yamasaki H, Choi H, Hirayama K, Shinohara M, Mori E. (2011). False item recognition in patients with Alzheimer's disease. *Neuropsychologia*. 49,1897-1902.
2. Adamaszek M, Olbrich S, Kirkby KC, Woldag H, Willert C, Heinrich A. (2013). Event-related potentials indicating impaired emotional attention in cerebellar stroke--a case study. *Neurosci Lett*. 548, 206-211.
3. Agarwal S, Suri V, Rishi A, Shukla B, Garg A, Sharma MC, Sinha S, Sarkar C. (2009). Glioneuronal tumor with neuropil-like islands: a new entity. *Neuropathology*. 29, 96-100.
4. Aghili M, Zahedi F, Rafiee E. (2009). Hydroxyglutaric aciduria and malignant brain tumor: a case report and literature review. *J Neurooncol*. 91, 233-236.
5. Al-Adawi S, Dorvlo AS, Burke DT, Huynh CC, Jacob L, Knight R, Shah MK, Al-Hussaini A. (2004). Apathy and depression in cross-cultural survivors of traumatic brain injury. *J Neuropsychiatry Clin Neurosci*. 16, 435-442.
6. Al-Chalabi A, Leigh PN. (2000). Recent advances in amyotrophic lateral sclerosis. *Curr Opin Neurol*. 13, 397-405.
7. Alipour F, Hashemi H, Piri N, Asghari H. (2010). Ocular manifestations of transconjunctival heroin abuse: a case report of an unusual route of substance abuse. *Cornea*. 29, 110-112.
8. Allan CA. (1995). Alcohol problems and anxiety disorders--a critical review. *Alcohol Alcohol*. 30, 145-151.
9. Allcock LM, Rowan EN, Steen IN, Wesnes K, Kenny RA, Burn DJ. (2009). Impaired attention predicts falling in Parkinson's disease. *Parkinsonism Relat Disord*. 15, 110-115.
10. Alva G, Cummings JL. (2008). Relative tolerability of Alzheimer's disease treatments. *Psychiatry (Edgmont)*. 5, 27-36.
11. Al-Zahrani MA, Elsayed YA. (2009). The impacts of substance abuse and dependence on neuropsychological functions in a sample of patients from Saudi Arabia. *Behav Brain Funct*. 5, 48.
12. Arboix A, García-Eroles L, Massons J, Oliveres M, Targa C. (2000). Hemorrhagic lacunar stroke. *Cerebrovasc Dis*. 10, 229-234.
13. Argyriou AA, Polychronopoulos P, Talelli P, Chroni E. (2006) F wave study in amyotrophic lateral sclerosis: assessment of balance between upper and lower motor neuron involvement. *Clin Neurophysiol*. 117, 1260-1265.
14. Armstrong TS, Cron SG, Bolanos EV, Gilbert MR, Kang DH. (2010). Risk factors for fatigue severity in primary brain tumor patients. *Cancer*. 116, 2707-2715.
15. Arnulf I, Nielsen J, Lohmann E, Schiefer J, Wild E, Jennum P, Konofal E, Walker M, Oudiette D, Tabrizi S, Durr A. (2008). Rapid eye movement sleep disturbances in Huntington disease. *Arch Neurol*. 65, 482-488.
16. Arria AM, Dohey MA, Mezzich AC, Bukstein OG, Van Thiel DH. (1995). Self-reported health problems and physical symptomatology in adolescent alcohol abusers. *J Adolesc Health*. 16, 226-231.
17. Ashby FG, Noble S, Filoteo JV, Waldron EM, Ell SW. (2003). Category learning deficits in Parkinson's disease. *Neuropsychology*. 17, 115-124.

18. Au A, Cheng C, Chan I, Leung P, Li P, Heaton RK. (2008). Subjective memory complaints, mood, and memory deficits among HIV/AIDS patients in Hong Kong. *J Clin Exp Neuropsychol*. 30, 338-348.
19. Ballard C, Corbett A. (2013). Agitation and aggression in people with Alzheimer's disease. *Curr Opin Psychiatry*. 26, 252-259.
20. Banihashemian K, Pour RE, Moazzen M. (2011). Frontal acquired brain injury, substance abuse and their common psychological symptoms in the Iranian population. *Brain Inj*. 25, 1249-1255.
21. Barthlen GM, Lange DJ. (2000). Unexpectedly severe sleep and respiratory pathology in patients with amyotrophic lateral sclerosis. *Eur J Neurol.* 7, 299-302.
22. Bauer LO, Ceballos NA, Shanley JD, Wolfson LI. (2005). Sensorimotor dysfunction in HIV/AIDS: effects of antiretroviral treatment and comorbid psychiatric disorders. *AIDS.* 19, 495-502.
23. Bauer LO. (2008). Psychiatric and neurophysiological predictors of obesity in HIV/AIDS. *Psychophysiology*. 45, 1055-1063.
24. Bay E, de-Leon MB. (2011). Chronic stress and fatigue-related quality of life after mild to moderate traumatic brain injury. *J Head Trauma Rehabil*. 26, 355-363.
25. Beck JC. (2004). Delusions, substance abuse, and serious violence. *J Am Acad Psychiatry Law*. 32, 169-172.
26. Becker JT, Caldararo R, Lopez OL, Dew MA, Dorst SK, Banks G. (1995). Qualitative features of the memory deficit associated with HIV infection and AIDS: cross-validation of a discriminant function classification scheme. *J Clin Exp Neuropsychol*. 17, 134-142.
27. Belanger HG, King-Kallimanis B, Nelson AL, Schonfeld L, Scott SG, Vanderploeg RD. (2008). Characterizing wandering behaviors in persons with traumatic brain injury residing in veterans health administration nursing homes. *Arch Phys Med Rehabil*. 89, 244-250.
28. Belanger HG, Kretzmer T, Vanderploeg RD, French LM. (2010). Symptom complaints following combat-related traumatic brain injury: relationship to traumatic brain injury severity and posttraumatic stress disorder. *J Int Neuropsychol Soc*. 16, 194-199.
29. Belcher HM, Shinitzky HE. (1998). Substance abuse in children: prediction, protection, and prevention. *Arch Pediatr Adolesc Med*. 152, 952-960.
30. Belmont A, Agar N, Hugeron C, Gallais B, Azouvi P. (2006). Fatigue and traumatic brain injury. *Ann Readapt Med Phys*. 49, 283-8, 370-4.
31. Benesch CG, McDaniel KD, Cox C, Hamill RW. (1993). End-stage Alzheimer's disease. Glasgow Coma Scale and the neurologic examination. *Arch Neurol*. 50, 1309-1315.
32. Benishek D, Wichowski HC. (2003). Dissociation in adults with a diagnosis of substance abuse. *Nurs Times*. 99, 34-36.
33. Bennett C, Baker K. (2001). HIV and AIDS: an overview. *Nurs Stand*. 15, 45-52
34. Bernstein GA, Victor AM, Pipal AJ, Williams KA. (2010). Comparison of clinical characteristics of pediatric autoimmune neuropsychiatric disorders associated with streptococcal infections and childhood obsessive-compulsive disorder. *J Child Adolesc Psychopharmacol.* 20, 333-340.
35. Bhalla RK, Papandonatos GD, Stern RA, Ott BR. (2007). Anxiety of Alzheimer's disease patients before and after a standardized on-road driving test. *Alzheimers Dement*. 3, 33-39.

36. Bhigjee AI. (2005). Seizures in HIV/AIDS: a southern African perspective. *Acta Neurol Scand Suppl*. 181, 4-7.
37. Bianchi-Demicheli F, Rollini C, Lovblad K, Ortigue S. (2010). Sleeping Beauty paraphilia: deviant desire in the context of bodily self-image disturbance in a patient with a fronto-parietal traumatic brain injury. *Med Sci Monit*. 16, CS15-17.
38. Binetti G, Bianchetti A, Padovani A, Lenzi G, De Leo D, Trabucchi M. (1993). Delusions in Alzheimer's disease and multi-infarct dementia. *Acta Neurol Scand*. 88, 5-9.
39. Birath CS, DeMarinis V, af Klinteberg B. (2010). Moods and expectancies of female alcohol drinking--an exploratory study. *Scand J Caring Sci*. 24, 472-481
40. Blaney SM, Boyett J, Friedman H, Gajjar A, Geyer R, Horowtiz M, Hunt D, Kieran M, Kun L, Packer R, Phillips P, Pollack IF, Prados M, Heideman R. (2005). Phase I clinical trial of mafosfamide in infants and children aged 3 years or younger with newly diagnosed embryonal tumors: a pediatric brain tumor consortium study (PBTC-001). *J Clin Oncol*. 23, 525-531.
41. Blonder LX, Slevin JT. (2011). Emotional dysfunction in Parkinson's disease. *Behav Neurol*. 24, 201-217.
42. Blyth BJ, Bazarian JJ. (2010). Traumatic alterations in consciousness: traumatic brain injury. *Emerg Med Clin North Am*. 28, 571-594.
43. Boele FW, Douw L, de Groot M, van Thuijl HF, Cleijne W, Heimans JJ, Taphoorn MJ, Reijneveld JC, Klein M. (2013). The effect of modafinil on fatigue, cognitive functioning, and mood in primary brain tumor patients: a multicenter randomized controlled trial. *Neuro Oncol*. 15, 1420-1428.
44. Bogdanova Y, Verfaellie M. (2012). Cognitive sequelae of blast-induced traumatic brain injury: recovery and rehabilitation. *Neuropsychol Rev*. 22, 4-20.
45. Bond GR, Pièche S, Sonicki Z, Gamaluddin H, El Guindi M, Sakr M, El Seddawy A, Abouzaid M, Youssef A, WHO EMRO Pediatric Insecticide Study Group. (2008). A clinical decision aid for triage of children younger than 5 years and with organophosphate or carbamate insecticide exposure in developing countries. *Ann Emerg Med*. 52, 617-622.
46. Bondi MW, Drake AI, Grant I. (1998). Verbal learning and memory in alcohol abusers and polysubstance abusers with concurrent alcohol abuse. *J Int Neuropsychol Soc*. 4, 319-328.
47. Bonnet AM, Czernecki V. (2013). Non-motor symptoms in Parkinson's disease: cognition and behavior. *Geriatr Psychol Neuropsychiatr Vieil*. 11, 295-304.
48. Bornschein S, Hausteiner C, Konrad F, Förstl H, Zilker T. (2006). Psychiatric morbidity and toxic burden in patients with environmental illness: a controlled study. *Psychosom Med*. 68, 104-109.
49. Broomhall LG, Clark CR, McFarlane AC, O'Donnell M, Bryant R, Creamer M, Silove D. (2009). Early stage assessment and course of acute stress disorder after mild traumatic brain injury. *J Nerv Ment Dis*. 197, 178-181.
50. Brust JC. (1998). Acute neurologic complications of drug and alcohol abuse. *Neurol Clin*. 16, 503-519.
51. Brust JC. (2006). Seizures and substance abuse: treatment considerations. *Neurology*. 67, S45-48.

52. Bryan CJ, Clemans TA, Hernandez AM, Rudd MD. (2013). Loss of consciousness, depression, posttraumatic stress disorder, and suicide risk among deployed military personnel with mild traumatic brain injury. *J Head Trauma Rehabil*. 28, 13-20.
53. Bryan CJ, Clemans TA. (2013). Repetitive traumatic brain injury, psychological symptoms, and suicide risk in a clinical sample of deployed military personnel. *JAMA Psychiatry*. 70, 686-691.
54. Budhachandra Y, Ramesh K, Sumitra G. (2007). Personality profile among Human Immunodeficiency Virus (HIV) positives and Acquired Immunodeficiency Syndrome (AIDS) patients of injecting drug users. *Kathmandu Univ Med J (KUMJ)*. 5, 38-41.
55. Bugalho P, Paiva T. (2011). Dream features in the early stages of Parkinson's disease. *J Neural Transm*. 118, 1613-1619.
56. Bunevicius A, Deltuva V, Tamasauskas S, Tamasauskas A, Laws ER Jr, Bunevicius R. (2013). Low triiodothyronine syndrome as a predictor of poor outcomes in patients undergoing brain tumor surgery: a pilot study: clinical article. *J Neurosurg*. 118, 1279-1287.
57. Burns A, Folstein S, Brandt J, Folstein M. (1990). Clinical assessment of irritability, aggression, and apathy in Huntington and Alzheimer disease. *J Nerv Ment Dis*. 178,20-26.
58. Burns A, Jacoby R, Levy R. (1990). Psychiatric phenomena in Alzheimer's disease. III: Disorders of mood. *Br J Psychiatry*. 157, 81-6, 92-4.
59. Byers V, Arrington ME, Finstuen K. (1990). Predictive risk factors associated with stroke patient falls in acute care settings. *J Neurosci Nurs*. 22, 147-154.
60. Caeiro L, Ferro JM, Pinho E Melo T, Canhão P, Figueira ML. (2013). Post-stroke apathy: an exploratory longitudinal study. *Cerebrovasc Dis*. 35, 507-513.
61. Caeyenberghs K, Leemans A, Geurts M, Taymans T, Vander Linden C, Smits-Engelsman BC, Sunaert S, Swinnen SP. (2010). Brain-behavior relationships in young traumatic brain injury patients: fractional anisotropy measures are highly correlated with dynamic visuomotor tracking performance. *Neuropsychologia*. 48,1472-1482.
62. Cardoso G, Alexandre J, Rosa A. (2010). Depression, anxiety and alcohol abuse in a gastroenterology intensive care unit: prevalence and detection. C*lin Pract Epidemiol Ment Health*. 6, 47-52.
63. Carman KB, Tutkun E, Yilmaz H, Dilber C, Dalkiran T, Cakir B, Arslantas D, Cesaretli Y, Aykanat SA. (2013). Acute mercury poisoning among children in two provinces of Turkey. *Eur J Pediatr*. 172, 821-827.
64. Cavanna AE, Martino D, Orth M, Giovannoni G, Stern JS, Robertson MM, Critchley HD. (2009). Neuropsychiatric-developmental model for the expression of tics, pervasive developmental disorder, and schizophreniform symptomatology associated with PANDAS. *World J Biol Psychiatry*. 10, 1037-1038.
65. Cengel-Kültür SE, Cöp E, Kara A, Cengiz AB, Uludağ AK, Unal F. (2009). The relationship between group A beta hemolytic streptococcal infection and psychiatric symptoms: a pilot study. *Turk J Pediatr*. 51, 317-324.
66. Chagas MH, Linares IM, Garcia GJ, Hallak JE, Tumas V, Crippa JA. (2013). Neuroimaging of depression in Parkinson's disease: a review. *Int Psychogeriatr*. 2, 1-9.

67. Chakravorty S, Grandner MA, Kranzler HR, Mavandadi S, Kling MA, Perlis ML, Oslin DW. (2013). Insomnia in alcohol dependence: predictors of symptoms in a sample of veterans referred from primary care. *Am J Addict*. 22, 266-270.
68. Chamelian L, Feinstein A. (2004). Outcome after mild to moderate traumatic brain injury: the role of dizziness. *Arch Phys Med Rehabil*. 85, 1662-1666.
69. Chen Y, Huang W. (2011). Non-impact, blast-induced mild TBI and PTSD: concepts and caveats. *Brain Inj*. 25, 641-650.
70. Chen Y. (2012). Organophosphate-induced brain damage: mechanisms, neuropsychiatric and neurological consequences, and potential therapeutic strategies. *Neurotoxicology*. 33, 391-400.
71. Chertkow H, Bub D, Seidenberg M. (1989). Priming and semantic memory loss in Alzheimer's disease. *Brain Lang*. 36, 420-446.
72. Christidi F, Zalonis I, Smyrnis N, Evdokimidis I. (2012). Selective attention and the three-process memory model for the interpretation of verbal free recall in amyotrophic lateral sclerosis. *J Int Neuropsychol Soc*. 18, 809-818.
73. Church MW, Abel EL. (1998). Fetal alcohol syndrome. Hearing, speech, language, and vestibular disorders. *Obstet Gynecol Clin North Am*. 25, 85-97.
74. Ciccarelli O, Behrens TE, Altmann DR, Orrell RW, Howard RS, Johansen-Berg H, Miller DH, Matthews PM, Thompson AJ. (2006). Probabilistic diffusion tractography: a potential tool to assess the rate of disease progression in amyotrophic lateral sclerosis. *Brain*. 129, 1859-71.
75. Ciraulo DA, Nace EP. (2000). Benzodiazepine treatment of anxiety or insomnia in substance abuse patients. *Am J Addict*. 9, 276-279.
76. Clark DB, Bukstein OG, Smith MG, Kaczynski NA, Mezzich AC, Donovan JE. (1995). Identifying anxiety disorders in adolescents hospitalized for alcohol abuse or dependence. *Psychiatr Serv*. 46, 618-620.
77. Clement JA, Williams EB, Waters C. (1993). The client with substance abuse/mental illness: mandate for collaboration. *Arch Psychiatr Nurs*. 7, 189-196.
78. Clusmann H, Schaller C, Schramm J. (2001). Fixed and dilated pupils after trauma, stroke, and previous intracranial surgery: management and outcome. *J Neurol Neurosurg Psychiatry*. 71, 175-181.
79. Cockerham GC, Goodrich GL, Weichel ED, Orcutt JC, Rizzo JF, Bower KS, Schuchard RA. (2009). Eye and visual function in traumatic brain injury. *J Rehabil Res Dev*. 46, 811-818.
80. Collen J, Orr N, Lettieri CJ, Carter K, Holley AB. (2012). Sleep disturbances among soldiers with combat-related traumatic brain injury. *Chest*. 142, 622-630.
81. Cooper DB, Mercado-Couch JM, Critchfield E, Kennedy J, Vanderploeg RD, DeVillibis C, Gaylord KM. (2010). Factors influencing cognitive functioning following mild traumatic brain injury in OIF/OEF burn patients. *NeuroRehabilitation*. 26, 233-238.
82. Coulehan JL, Zettler-Segal M, Block M, McClelland M, Schulberg HC. (1987). Recognition of alcoholism and substance abuse in primary care patients. *Arch Intern Med*. 147, 349-352.
83. Cowley DS. (1992). Alcohol abuse, substance abuse, and panic disorder. *Am J Med*. 92, 41S-48S.

84. Craufurd D, Thompson JC, Snowden JS. (2001). Behavioral changes in Huntington Disease. *Neuropsychiatry Neuropsychol Behav Neurol*. 14, 219-226.
85. Cumming TB, Collier J, Thrift AG, Bernhardt J. (2008). The effect of very early mobilisation after stroke on psychological well-being. *J Rehabil Med.* 40, 609-614.
86. Czell D, Andersen PM, Neuwirth C, Morita M, Weber M. (2013). Progressive aphasia as the presenting symptom in a patient with amyotrophic lateral sclerosis with a novel mutation in the OPTN gene. *Amyotroph Lateral Scler Frontotemporal Degener*. 14, 138-140.
87. Da Rocha PC, Barroso MT, Dantas AA, Melo LP, Campos TF. (2013). Predictive factors of subjective sleep quality and insomnia complaint in patients with stroke: implications for clinical practice. *An Acad Bras Cienc*. 85, 1197-1206.
88. Dalus D, Mathew AJ, Somarajan Pillai S. (2013). Formic acid poisoning in a tertiary care center in South India: A 2-year retrospective analysis of clinical profile and predictors of mortality. *J Emerg Med*. 44, 373-380.
89. D'Amore MM, Cheng DM, Allensworth-Davies D, Samet JH, Saitz R. (2012). Disparities in safe sex counseling & behavior among individuals with substance dependence: a cross-sectional study. *Reprod Health.* 9, 35.
90. Dansky BS, Roitzsch JC, Brady KT, Saladin ME. (1997). Posttraumatic stress disorder and substance abuse: use of research in a clinical setting. *J Trauma Stress*. 10, 141-148.
91. Darchini-Maragheh E, Nemati-Karimooy H, Hasanabadi H, Balali-Mood M. (2012). Delayed neurological complications of sulphur mustard and tabun poisoning in 43 Iranian veterans. *Basic Clin Pharmacol Toxicol*. 111, 426-432.
92. Das A, Radhakrishnan A. (2012). A case of PANDAS with Kleine-Levin type periodic hypersomnia. *Sleep Med*. 13, 319-320.
93. Dassanayake T, Weerasinghe V, Dangahadeniya U, Kularatne K, Dawson A, Karalliedde L, Senanayake N. (2007). Cognitive processing of visual stimuli in patients with organophosphate insecticide poisoning. *Neurology*. 68, 2027-2030.
94. Daughters SB, Richards JM, Gorka SM, Sinha R. (2009). HPA axis response to psychological stress and treatment retention in residential substance abuse treatment: a prospective study. *Drug Alcohol Depend*. 105, 202-208.
95. Davis AE. (2000). Cognitive impairments following traumatic brain injury. Etiologies and interventions. *Crit Care Nurs Clin North Am*. 12, 447-456.
96. De Bernardo GL, Newcomb M, Toth A, Richey G, Mendoza R. (2002). Comorbid psychiatric and alcohol abuse/dependence disorders: psychosocial stress, abuse, and personal history factors of those in treatment. *J Addict Dis*. 21, 43-59.
97. de Chazeron I, Llorca PM, Chéreau-Boudet I, Blanc O, Perriot J, Ouchchane L, Ulla M, Debilly B, Derost P, Durif F. (2011). Hypersexuality and pathological gambling in Parkinson's disease: A cross-sectional case-control study. *Mov Disord*. 26, 2127-2130.
98. de Coster L, Leentjens AF, Lodder J, Verhey FR. (2005). The sensitivity of somatic symptoms in post-stroke depression: a discriminant analytic approach. *Int J Geriatr Psychiatry*. 20, 358-362.
99. De Kruijk JR, Leffers P, Menheere PP, Meerhoff S, Rutten J, Twijnstra A. (2002). Prediction of post-traumatic complaints after mild traumatic brain injury: early symptoms and biochemical markers. *J Neurol Neurosurg Psychiatry*. 73, 727-732.

100. De Kruijk JR, Twijnstra A, Leffers P. (2001). Diagnostic criteria and differential diagnosis of mild traumatic brain injury. *Brain Inj*. 15, 99-106.
101. de Smet Y, Ruberg M, Serdaru M, Dubois B, Lhermitte F, Agid Y. (1982). Confusion, dementia and anticholinergics in Parkinson's disease. *J Neurol Neurosurg Psychiatry*. 45, 1161-1164.
102. de Souza-Talarico JN, Chaves EC, Nitrini R, Caramelli P. (2009). Stress and coping in older people with Alzheimer's disease. *J Clin Nurs.* 18, 457-465
103. Deb S, Burns J. (2007). Neuropsychiatric consequences of traumatic brain injury: a comparison between two age groups. *Brain Inj*. 21, 301-307.
104. DeFranco C, Tarbox AR, McLaughlin EJ. (1985). Cognitive deficits as a function of years of alcohol abuse. *Am J Drug Alcohol Abuse*. 11, 279-293.
105. Delgado E, McConnell R, Miranda J, Keifer M, Lundberg I, Partanen T, Wesseling C. (2004). Central nervous system effects of acute organophosphate poisoning in a two-year follow-up. *Scand J Work Environ Health*. 30, 362-370.
106. Demetriades AK. (2002). Functional neuroimaging in Alzheimer's type dementia. *J Neurol Sci.* 203-204, 247-251.
107. Dethier M, Blairy S, Rosenberg H, McDonald S. (2012). Spontaneous and posed emotional facial expressions following severe traumatic brain injury. *J Clin Exp Neuropsychol*. 34, 936-947.
108. Deutsch LH, Rovner BW. (1991). Agitation and other noncognitive abnormalities in Alzheimer's disease. *Psychiatr Clin North Am*. 14, 341-351
109. Diederich N, Karenberg A, Peters UH. (1988). Psychopathologic pictures in HIV infection: AIDS lethargy and AIDS dementia. *Fortschr Neurol Psychiatr*. 56, 173-185.
110. Dilley KJ, Lockart B. (2009). The pediatric brain tumor late effects clinic. *Cancer Treat Res.* 150, 97-109.
111. Donat DC. (1986). Semantic and visual memory after alcohol abuse. *J Clin Psychol.* 42, 537-539.
112. Driver-Dunckley ED, Hoxworth JM, Patel NP, Bosch EP, Goodman BP. (2010). Superficial siderosis mimicking amyotrophic lateral sclerosis. *J Clin Neuromuscul Dis*. 11, 137-144.
113. Durrant JD, Palmer CV, Lunner T. (2005). Analysis of counted behaviors in a single-subject design: modeling of hearing-aid intervention in hearing-impaired patients with Alzheimer's disease. *Int J Audiol*. 44, 31-38.
114. Dutta TM, Josiah AF, Cronin CA, Wittenberg GF, Cole JW. (2013). Altered taste and stroke: a case report and literature review. *Top Stroke Rehabil*. 20, 78-86.
115. Easton CJ, Sacco KA, Neavins TM, Wupperman P, George TP. (2008). Neurocognitive performance among alcohol dependent men with and without physical violence toward their partners: a preliminary report. *Am J Drug Alcohol Abuse*. 34, 29-37.
116. Echevarría ME, Weinstein JL. (2009). Ocular consequences and late effects of brain tumor treatments. *Cancer Treat Res*. 150, 183-194.
117. Eife R, Weiss M, Barros V, Sigmund B, Goriup U, Komb D, Wolf W, Kittel J, Schramel P, Reiter K. (1999). Chronic poisoning by copper in tap water: I. Copper intoxications with predominantly gastointestinal symptoms. *Eur J Med Res*. 4, 219-223.

118. Elia J, Dell ML, Friedman DF, Zimmerman RA, Balamuth N, Ahmed AA, Pati S. (2005). PANDAS with catatonia: a case report. Therapeutic response to lorazepam and plasmapheresis. *J Am Acad Child Adolesc Psychiatry*. 44, 1145-1150.
119. Ellis C, Focht KL, Grubaugh AL. (2013). Perceptions of stroke recovery: An exclusion of communication and cognition. *NeuroRehabilitation*. 33, 233-239.
120. Erstad BL, Cotugno CL. (1995). Management of alcohol withdrawal. *Am J Health Syst Pharm*. 52, 697-709.
121. Ettenhofer ML, Melrose RJ, Delawalla Z, Castellon SA, Okonek A. (2012). Correlates of functional status among OEF/OIF veterans with a history of traumatic brain injury. *Mil Med.* 177, 1272-1278.
122. Fabbrini G, Latorre A, Suppa A, Bloise M, Frontoni M, Berardelli A. (2013). Fatigue in Parkinson's disease: motor or non-motor symptom? *Parkinsonism Relat Disord*. 19, 148-152.
123. Fals-Stewart W, Angarano K. (1994). Obsessive-compulsive disorder among patients entering substance abuse treatment. Prevalence and accuracy of diagnosis. *J Nerv Ment Dis.* 182, 715-719.
124. Famularo R, Stone K, Popper C. (1985). Preadolescent alcohol abuse and dependence. *Am J Psychiatry*. 142, 1187-1189.
125. Fann J, Hart T; University of Washington Model Systems Knowledge Translation Center. (2013). Depression after traumatic brain injury. *Arch Phys Med Rehabil*. 94, 801-802.
126. Fausti SA, Wilmington DJ, Gallun FJ, Myers PJ, Henry JA. (2009). Auditory and vestibular dysfunction associated with blast-related traumatic brain injury. *J Rehabil Res Dev*. 46, 797-810.
127. Favrole P, Jehel L, Levy P, Descombes S, Muresan IP, Manifacier MJ, Alamowitch S. (2013). Frequency and predictors of post-traumatic stress disorder after stroke: a pilot study. *J Neurol Sci*. 327, 35-40.
128. Ferrando SJ, Wapenyi K. (2002). Psychopharmacological treatment of patients with HIV and AIDS. *Psychiatr Q*. 73, 33-49.
129. Finkel AG, Yerry J, Scher A, Choi YS. (1992). Headaches in soldiers with mild traumatic brain injury: findings and phenomenologic descriptions. *Headache*. 52, 957-965.
130. Fleminger S. (1992). Control of simultaneous movements distinguishes depressive motor retardation from Parkinson's disease and neuroleptic parkinsonism. *Brain*. 115, 1459-1480.
131. Flood M, Buckwalter KC. (2009). Recommendations for mental health care of older adults: Part 2--an overview of dementia, delirium, and substance abuse. *J Gerontol Nurs.* 35, 35-47
132. Flynn FG. (2010). Memory impairment after mild traumatic brain injury. Continuum *(Minneap Minn)*. 16, 79-109.
133. Ford JD, Schnurr PP, Friedman MJ, Green BL, Adams G, Jex S. (2004). Posttraumatic stress disorder symptoms, physical health, and health care utilization 50 years after repeated exposure to a toxic gas. *J Trauma Stress*. 17, 185-194.
134. Formby C, Phillips DE, Thomas RG. (1987). Hearing loss among stroke patients. *Ear Hear*. 8, 326-332.

135. Förstl H, Dalgalarrondo P, Riecher-Rössler A, Lotz M, Geiger-Kabisch C, Hentschel F. (1994). Organic factors and the clinical features of late paranoid psychosis: a comparison with Alzheimer's disease and normal ageing. *Acta Psychiatr Scand*. 89, 335-340.
136. Fortin A, Lefebvre MB, Ptito M. (2010). Traumatic brain injury and olfactory deficits: the tale of two smell tests! *Brain Inj*. 24, 27-33.
137. Foster PS, Drago V, Mendez K, Witt JC, Crucian GP, Heilman KM. (2013). Mood disturbances and cognitive functioning in Parkinson's disease: the effects of disease duration and side of onset of motor symptoms. *J Clin Exp Neuropsychol*. 35, 71-82.
138. Franken IH, Hendriks VM. (2001). Screening and diagnosis of anxiety and mood disorders in substance abuse patients. *Am J Addict*. 10, 30-39.
139. French LM. (2010). Military traumatic brain injury: an examination of important differences. *Ann N Y Acad Sci*. 1208, 38-45
140. Friedman D, Honig LS, Scarmeas N. (2012). Seizures and epilepsy in Alzheimer's disease. *CNS Neurosci Ther*. 18, 285-294.
141. Fritz NE, Basso DM. (2013). Dual-task training for balance and mobility in a person with severe traumatic brain injury: a case study. *J Neurol Phys Ther*. 37, 37-43.
142. Fujii D, Ahmed I.(2002). Psychotic disorder following traumatic brain injury: a conceptual framework. *Cogn Neuropsychiatry*. 7, 41-62.
143. Fujii M, Fujita K, Hiramatsu H, Miyamoto T. (1998). Cases of two patients whose food aversions disappeared following severe traumatic brain injury. *Brain Inj*. 12, 709-713.
144. Gall SL, Donnan G, Dewey HM, Macdonell R, Sturm J, Gilligan A, Srikanth V, Thrift AG. (2010). Sex differences in presentation, severity, and management of stroke in a population-based study. *Neurology*. 74, 975-981.
145. Galski T, Bruno RL, Zorowitz R, Walker J. (1993). Predicting length of stay, functional outcome, and aftercare in the rehabilitation of stroke patients. The dominant role of higher-order cognition. *Stroke*. 24, 1794-1800.
146. Ganry O, Joly JP, Queval MP, Dubreuil A. (2000). Prevalence of alcohol problems among elderly patients in a university hospital. *Addiction*. 95, 107-113.
147. Garvey MA, Perlmutter SJ, Allen AJ, Hamburger S, Lougee L, Leonard HL, Witowski ME, Dubbert B, Swedo SE. (1999). A pilot study of penicillin prophylaxis for neuropsychiatric exacerbations triggered by streptococcal infections. *Biol Psychiatry*. 45, 1564-1571.
148. Gehring K, Patwardhan SY, Collins R, Groves MD, Etzel CJ, Meyers CA, Wefel JS. (2012). A randomized trial on the efficacy of methylphenidate and modafinil for improving cognitive functioning and symptoms in patients with a primary brain tumor. *J Neurooncol*. 107, 165-174.
149. Geldmacher DS, Hills EC. (1997). Effect of stimulus number, target-to-distractor ratio, and motor speed on visual spatial search quality following traumatic brain injury. *Brain Inj*. 11, 59-66.
150. Genuis SJ. (2009). Toxicant exposure and mental health--individual, social, and public health considerations. *J Forensic Sci*. 54, 474-477.
151. Georgiou-Karistianis N, Churchyard A, Chiu E, Bradshaw JL. (2002). Reorientation of attention in Huntington disease. *Neuropsychiatry Neuropsychol Behav Neurol*. 15, 225-231.

152. Georgiou-Karistianis N, Farrow M, Wilson-Ching M, Churchyard A, Bradshaw JL, Sheppard DM. (2012). Deficits in selective attention in symptomatic Huntington disease: assessment using an attentional blink paradigm. *Cogn Behav Neurol*. 25, 1-6.
153. Gil R, Arroyo-Anllo EM, Ingrand P, Gil M, Neau JP, Ornon C, Bonnaud V. (2001). Self-consciousness and Alzheimer's disease. *Acta Neurol Scand*. 104, 296-300.
154. Gjerstad MD, Wentzel-Larsen T, Aarsland D, Larsen JP. (2007). Insomnia in Parkinson's disease: frequency and progression over time. *J Neurol Neurosurg Psychiatry*. 78, 476-479.
155. Goerke D, Kumra S. (2013). Substance abuse and psychosis. *Child Adolesc Psychiatr Clin N Am*. 22, 643-654.
156. Goffaux P, Boudrias M, Mathieu D, Charpentier C, Veilleux N, Fortin D. (2009). Development of a concise QOL questionnaire for brain tumor patients. *Can J Neurol Sci*. 36, 340-348.
157. Goffaux P, Fortin D. (2010). Brain tumor headaches: from bedside to bench. *Neurosurgery*. 67, 459-466.
158. Gomez-Hernandez R, Max JE, Kosier T, Paradiso S, Robinson RG. (1997). Social impairment and depression after traumatic brain injury. *Arch Phys Med Rehabil*. 78, 1321-1326.
159. Gómez-Tortosa E, del Barrio A, García Ruiz PJ, Pernaute RS, Benítez J, Barroso A, Jiménez FJ, García Yébenes J. (1998). Severity of cognitive impairment in juvenile and late-onset Huntington disease. *Arch Neurol*. 55, 835-843.
160. Gordon AJ, Wentz CM, Gibbon JL, Mason AD, Freyder PJ, O'Toole TP. (2001). Relationships between patient characteristics and unsuccessful substance abuse detoxification. *J Addict Dis*. 20, 41-53.
161. Gramowski A, Jügelt K, Schröder OH, Weiss DG, Mitzner S. (2011). Acute functional neurotoxicity of lanthanum(III) in primary cortical networks. *Toxicol Sci*. 120, 173-183.
162. Grant JS, Glandon GL, Elliott TR, Giger JN, Weaver M. (2004). Caregiving problems and feelings experienced by family caregivers of stroke survivors the first month after discharge. *Int J Rehabil Res*. 27, 105-111.
163. Gray S, Borgundvaag B, Sirvastava A, Randall I, Kahan M. (2010). Feasibility and reliability of the SHOT: A short scale for measuring pretreatment severity of alcohol withdrawal in the emergency department. *Acad Emerg Med*. 17, 1048-1054.
164. Greve KW, Sherwin E, Stanford MS, Mathias C, Love J, Ramzinski P. (2001). Personality and neurocognitive correlates of impulsive aggression in long-term survivors of severe traumatic brain injury. *Brain Inj*. 15, 255-262.
165. Greydanus DE, Patel DR. (2003). Substance abuse in adolescents: a complex conundrum for the clinician. *Pediatr Clin North Am*. 50, 1179-1223
166. Grmec S, Mally S, Klemen P. (2004). Glasgow Coma Scale score and QTc interval in the prognosis of organophosphate poisoning. *Acad Emerg Med*. 11, 925-930.
167. Grochmal-Bach B, Pachalska M, Markiewicz K, Tomaszewski W, Olszewski H, Pufal A. (2009). Rehabilitation of a patient with aphasia due to severe traumatic brain injury. *Med Sci Monit*. 15, CS67-76.
168. Grossman AB, Woolley-Levine S, Bradley WG, Miller RG. (2007). Detecting neurobehavioral changes in amyotrophic lateral sclerosis. *Amyotroph Lateral Scler*. 8, 56-61.

169. Grossman M, Anderson C, Khan A, Avants B, Elman L, McCluskey L. (2008). Impaired action knowledge in amyotrophic lateral sclerosis. *Neurology*. 71, 1396-1401.
170. Guerreiro DF, Navarro R, Silva M, Carvalho M, Gois C. (2009). Psychosis secondary to traumatic brain injury. *Brain Inj*. 23, 358-361.
171. Guo JX, Hu L, Yand PZ, Tanabe K, Miyatalre M, Chen Y. (2007). Chronic arsenic poisoning in drinking water in Inner Mongolia and its associated health effects. *J Environ Sci Health A Tox Hazard Subst Environ Eng.* 42, 1853-1858.
172. Hammond K, Graybill T, Speiss SE, Lu J, Leikin JB. (2009). A complicated hospitalization following dilute ammonium chloride ingestion. *J Med Toxicol.* 5, 218-222.
173. Handratta V, Hsu E, Vento J, Yang C, Tanev K. (2010). Neuroimaging findings and brain-behavioral correlates in a former boxer with chronic traumatic brain injury. *Neurocase*. 16, 125-134.
174. Hartman DE. (1998). Missed diagnoses and misdiagnoses of environmental toxicant exposure. The psychiatry of toxic exposure and multiple chemical sensitivity. *Psychiatr Clin North Am*. 21, 659-670, vii.
175. Hartwigsen G, Siebner HR. (2013). Novel methods to study aphasia recovery after stroke. *Front Neurol Neurosci*. 32, 101-111.
176. Hazelton LD, Sterns GL, Chisholm T. (2003). Decision-making capacity and alcohol abuse: clinical and ethical considerations in personal care choices. *Gen Hosp Psychiatry*. 25,130-135.
177. Heetun ZS, Quigley EM. (2012). Gastroparesis and Parkinson's disease: a systematic review. *Parkinsonism Relat Disord*. 18, 433-440
178. Heinz A, Denke C, Ernst G. (1999). Drug-induced headache--pathomechanisms of addiction. *Schmerz*. 13, 304-314.
179. Heinze BM, Vinck BM, Hofmeyr LM, Swanepoel DW. (2013). Vestibular involvement in adults with HIV/AIDS. *Auris Nasus Larynx*. Oct 19. doi:pii: S0385-8146(13)00181-8.
180. Helmy A, Kirkpatrick PJ, Seeley HM, Corteen E, Menon DK, Hutchinson PJ. (2012). Fixed, dilated pupils following traumatic brain injury: historical perspectives, causes and ophthalmological sequelae. *Acta Neurochir Suppl*. 114, 295-299.
181. Hemmerle AM, Herman JP, Seroogy KB. (2012). Stress, depression and Parkinson's disease. *Exp Neurol*. 233, 79-86.
182. Henley DB, Sundell KL, Sethuraman G, Siemers ER. (2012). Alzheimer's Disease Neuroimaging Initiative. Safety profile of Alzheimer's disease populations in Alzheimer's Disease Neuroimaging Initiative and other 18-month studies. *Alzheimers Dement*. 8, 407-416.
183. Himanen L, Portin R, Tenovuo O, Taiminen T, Koponen S, Hiekkanen H, Helenius H. (2009). Attention and depressive symptoms in chronic phase after traumatic brain injury. *Brain Inj*. 23, 220-227.
184. Hirshberg A, Lerman Y. (1984). Clinical problems in organophosphate insecticide poisoning: the use of a computerized information system. *Fundam Appl Toxicol*. 4, S209-214.

185. Hoefer M, Allison SC, Schauer GF, Neuhaus JM, Hall J, Dang JN, Weiner MW, Miller BL, Rosen HJ. (2008). Fear conditioning in frontotemporal lobar degeneration and Alzheimer's disease. *Brain*. 131, 1646-1657.
186. Hofer H, Frigerio S, Frischknecht E, Gassmann D, Gutbrod K, Müri RM. (2013). Diagnosis and treatment of an obsessive-compulsive disorder following traumatic brain injury: a single case and review of the literature. *Neurocase*. 19, 390-400.
187. Holroyd S, Currie L, Wooten GF. (2001). Prospective study of hallucinations and delusions in Parkinson's disease. *J Neurol Neurosurg Psychiatry*. 70, 734-738.
188. Holzemer WL, Henry SB, Stewart A, Janson-Bjerklie S. (1993). The HIV quality audit marker (HIV-QAM): an outcome measure for hospitalized AIDS patients. *Qual Life Res*. 2, 99-107.
189. Honig PJ, Charney EB. (1982). Children with brain tumor headaches. Distinguishing features. *Am J Dis Child*. 136, 121-124.
190. Horner MD, Harvey RT, Denier CA. (1999). Self-report and objective measures of cognitive deficit in patients entering substance abuse treatment. *Psychiatry Res*. 86, 155-161.
191. Horvath J, Herrmann FR, Burkhard PR, Bouras C, Kövari E. (2013). Neuropathology of dementia in a large cohort of patients with Parkinson's disease. *Parkinsonism Relat Disord*. 19, 864-868.
192. Hovestadt A, de Jong GJ, Meerwaldt JD. (1987). Spatial disorientation as an early symptom of Parkinson's disease. *Neurology*. 37, 485-487.
193. Hsiung PC, Tsai YF. (2000). Stressors of living with HIV/AIDS: patients' perspectives. *Kaohsiung J Med Sci*. 16, 148-155.
194. Hudak AM, Trivedi K, Harper CR, Booker K, Caesar RR, Agostini M, Van Ness PC, Diaz-Arrastia R. (2004). Evaluation of seizure-like episodes in survivors of moderate and severe traumatic brain injury. *J Head Trauma Rehabil*. 19, 290-295.
195. Hunter MD, Robinson IC, Neilson S. (1993). The functional and psychological status of patients with amyotrophic lateral sclerosis: some implications for rehabilitation. Disabil *Rehabil*. 15, 119-126.
196. Iannotti CA, Hall GS, Procop GW, Tuohy MJ, Staugaitis SM, Weil RJ. (2009). Solitary Nocardia farcinica brain abscess in an immunocompetent adult mimicking metastatic brain tumor: rapid diagnosis by pyrosequencing and successful treatment. *Surg Neurol*. 72, 74-79.
197. Ibarretxe-Bilbao N, Zarei M, Junque C, Marti MJ, Segura B, Vendrell P, Valldeoriola F, Bargallo N, Tolosa E. (2011). Dysfunctions of cerebral networks precede recognition memory deficits in early Parkinson's disease. *Neuroimage*. 57, 589-597.
198. Ille R, Schäfer A, Scharmüller W, Enzinger C, Schöggl H, Kapfhammer HP, Schienle A. (2011). Emotion recognition and experience in Huntington disease: a voxel-based morphometry study. *J Psychiatry Neurosci*. 36, 383-390.
199. Inelman EM, Gasparini G, Enzi G. (2005). HIV/AIDS in older adults: a case report and literature review. *Geriatrics*. 60, 26-30.
200. Inzitari M, Giné-Garriga M, Martinez B, Perez-Fernandez M, Barranco-Rubia E, Lleó A, Salvà-Casanovas A. (2013). Cerebrovascular disease and gait and balance impairment in mild to moderate Alzheimer's disease. *J Nutr Health Aging*. 17, 45-48.

201. Ishizaki J, Meguro K, Nara N, Kasai M, Yamadori A. (2013). Impaired shifting of visuospatial attention in Alzheimer's disease as shown by the covert orienting paradigm: implications for visual construction disability. *Behav Neurol*. 26, 121-129.
202. Iverson KM, Hendricks AM, Kimerling R, Krengel M, Meterko M, Stolzmann KL, Baker E, Pogoda TK, Vasterling JJ, Lew HL. (2011). Psychiatric diagnoses and neurobehavioral symptom severity among OEF/OIF VA patients with deployment-related traumatic brain injury: a gender comparison. *Womens Health Issues*. 21, S210-217.
203. Jason GW, Suchowersky O, Pajurkova EM, Graham L, Klimek ML, Garber AT, Poirier-Heine D. (1997). Cognitive manifestations of Huntington disease in relation to genetic structure and clinical onset. *Arch Neurol*. 54, 1081-1088.
204. Jasova D, Bob P, Fedor-Freybergh P. (2007). Alcohol craving, limbic irritability, and stress. *Med Sci Monit*. 13, CR543-547.
205. Jayasinghe SS. (2012). Effects of acute organophosphate ingestion on cognitive function, assessed with the mini mental state examination. *J Postgrad Med*. 58, 171-175.
206. Jhee SS, Shiovitz T, Hartman RD, Messina J, Anand R, Sramek J, Cutler NR. (2002). Centrally acting antiemetics mitigate nausea and vomiting in patients with Alzheimer's disease who receive rivastigmine. *Clin Neuropharmacol*. 25, 122-123.
207. Johkura K, Yoshida TN, Kudo Y, Nakae Y, Momoo T, Kuroiwa Y. (2012). Cilostazol versus aspirin therapy in patients with chronic dizziness after ischemic stroke. *Clin Neurol Neurosurg*. 114, 876-880.
208. Joray S, Herrmann F, Mulligan R, Schnider A. (2004). Mechanism of disorientation in Alzheimer's disease. *Eur Neurol*. 52, 193-197.
209. Jorge R, Robinson RG. (2003). Mood disorders following traumatic brain injury. *Int Rev Psychiatry*. 15, 317-327.
210. Jorge RE, Robinson RG, Starkstein SE, Arndt SV, Forrester AW, Geisler FH. (1993). Secondary mania following traumatic brain injury. *Am J Psychiatry*. 150, 916-921.
211. Jorge RE, Robinson RG, Starkstein SE, Arndt SV. (1993). Depression and anxiety following traumatic brain injury. *J Neuropsychiatry Clin Neurosci*. 5, 369-374.
212. Joubert J, Joubert PH. (1988). Chorea and psychiatric changes in organophosphate poisoning. A report of 2 further cases. *S Afr Med J*. 74, 32-34.
213. Junqueira P, Bellucci S, Rossini S, Reimão R. (2008). Women living with HIV/AIDS: sleep impairment, anxiety and depression symptoms. *Arq Neuropsiquiatr*. 66, 817-820.
214. Kampov-Polevoy AB, Ziedonis D, Steinberg ML, Pinsky I, Krejci J, Eick C, Boland G, Khalitov E, Crews FT. (2003). Association between sweet preference and paternal history of alcoholism in psychiatric and substance abuse patients. *Alcohol Clin Exp Res*. 27, 1929-1936.
215. Kangas M, Tate RL, Williams JR, Smee RI. (2012). The effects of radiotherapy on psychosocial and cognitive functioning in adults with a primary brain tumor: a prospective evaluation. *Neuro Oncol*. 14, 1485-1502.
216. Kashihara K, Hanaoka A, Imamura T. (2011). Frequency and characteristics of taste impairment in patients with Parkinson's disease: results of a clinical interview. *Intern Med*. 50, 2311-2315.
217. Kehayov II, Kitov BD, Zhelyazkov CB, Raykov SD, Davarski AN. (2012). Neurocognitive impairments in brain tumor patients. *Folia Med (Plovdiv)*. 54, 14-21.

218. Kehe K, Thiermann H, Balszuweit F, Eyer F, Steinritz D, Zilker T. (2009). Acute effects of sulfur mustard injury--Munich experiences. *Toxicology*. 263, 3-8.
219. Keir ST. (2011). Effect of massage therapy on stress levels and quality of life in brain tumor patients--observations from a pilot study. *Support Care Cancer*. 19, 711-715.
220. Kennedy JE, Lumpkin RJ, Grissom JR. (2006). A survey of mild traumatic brain injury treatment in the emergency room and primary care medical clinics. *Mil Med*. 171, 516-521.
221. Kerai JH, Bracewell RM, Hindle JV, Leek EC. (2012). Visuospatial transformation impairments in Parkinson's disease. *J Clin Exp Neuropsychol*. 34, 1053-1064.
222. Kestelyn PG, Cunningham ET Jr. (2001). HIV/AIDS and blindness. *Bull World Health Organ*. 79, 208-213.
223. Khan F, Kennedy G, Spence VA, Newton DJ, Belch JJ. (2004). Peripheral cholinergic function in humans with chronic fatigue syndrome, Gulf War syndrome and with illness following organophosphate exposure. *Clin Sci (Lond)*. 106, 183-189.
224. Kim EJ, Kim DY, Kim WH, Lee KL, Yoon YH, Park JM, Shin JI, Kim SK, Kim DG. (2012). Fear of falling in subacute hemiplegic stroke patients: associating factors and correlations with quality of life. *Ann Rehabil Med*. 36, 797-803.
225. Kim SW, Grant JE, Kim SI, Swanson TA, Bernstein GA, Jaszcz WB, Williams KA, Schlievert PM. (2004). A possible association of recurrent streptococcal infections and acute onset of obsessive-compulsive disorder. *J Neuropsychiatry Clin Neurosci*. 16, 252-260.
226. Kirkwood SC, Su JL, Conneally P, Foroud T. (2001). Progression of symptoms in the early and middle stages of Huntington disease. *Arch Neurol*. 58, 273-278.
227. Kluger A, Gianutsos JG, Golomb J, Ferris SH, Reisberg B. (1997). Motor/psychomotor dysfunction in normal aging, mild cognitive decline, and early Alzheimer's disease: diagnostic and differential diagnostic features. *Int Psychogeriatr*. 9, 307-316
228. Kocak Y, Ozturk S, Ege F, Ekmekci H. (2012). A useful new coma scale in acute stroke patients: FOUR score. *Anaesth Intensive Care*. 40, 131-136.
229. Köhler S. (1994). Quantitative characterization of verbal learning deficits in patients with Alzheimer's disease. *J Clin Exp Neuropsychol*. 16, 749-753.
230. Kolitz BP, Vanderploeg RD, Curtiss G. (2003). Development of the Key Behaviors Change Inventory: a traumatic brain injury behavioral outcome assessment instrument. *Arch Phys Med Rehabil*. 84, 277-284.
231. Koponen S, Taiminen T, Portin R, Himanen L, Isoniemi H, Heinonen H, Hinkka S, Tenovuo O. (2002). Axis I and II psychiatric disorders after traumatic brain injury: a 30-year follow-up study. *Am J Psychiatry*. 159, 1315-1321.
232. Kranzler HR, Liebowitz NR. (1988). Anxiety and depression in substance abuse: clinical implications. *Med Clin North Am*. 72, 867-885.
233. Kranzler HR, Manu P, Hesselbrock VM, Lane TJ, Matthews DA. (1991). Substance use disorders in patients with chronic fatigue. *Hosp Community Psychiatry*. 42, 924-928.
234. Krogias C, Strassburger K, Eyding J, Gold R, Norra C, Juckel G, Saft C, Ninphius D. (2011). Depression in patients with Huntington disease correlates with alterations of the brain stem raphe depicted by transcranial sonography. *J Psychiatry Neurosci*. 36, 187-194.

235. Kumar S, Rao SL, Chandramouli BA, Pillai S. (2013). Reduced contribution of executive functions in impaired working memory performance in mild traumatic brain injury patients. *Clin Neurol Neurosurg*. 115, 1326-1332.
236. Kummer A, Cardoso F, Teixeira AL. (2008). Frequency of social phobia and psychometric properties of the Liebowitz social anxiety scale in Parkinson's disease. *Mov Disord*. 23, 1739-1743.
237. Kung WM, Tsai SH, Chiu WT, Hung KS, Wang SP, Lin JW, Lin MS. (2011). Correlation between Glasgow coma score components and survival in patients with traumatic brain injury. *Injury*. 42, 940-944.
238. Kuo HC, Huang CC, Chu CC, Chu NS. (2006). Axonal polyneuropathy after acute dimethylamine borane intoxication. *Arch Neurol*. 63, 1009-1012.
239. Kurt A, Nijboer F, Matuz T, Kübler A. (2007). Depression and anxiety in individuals with amyotrophic lateral sclerosis: epidemiology and management. *CNS Drugs*. 21, 279-291.
240. Kusoffsky A, Apel I, Hirschfeld H. (2001). Reaching-lifting-placing task during standing after stroke: Coordination among ground forces, ankle muscle activity, and hand movement. *Arch Phys Med Rehabil*. 82, 650-660.
241. Kwak R, Saso S, Onuma T, Suzuki J. (1979). Brain tumor with ipsilateral cerebral hemiatrophy in children. *J Neurosurg Sci*. 23, 141-152.
242. Labropoulos N, Nandivada P, Bekelis K. (2011). Stroke of the posterior cerebral circulation. *Int Angiol*. 30, 105-114.
243. Lacour M, Zunder T, Dettenkofer M, Schönbeck S, Lüdtke R, Scheidt C. (2002). An interdisciplinary therapeutic approach for dealing with patients attributing chronic fatigue and functional memory disorders to environmental poisoning--a pilot study. *Int J Hyg Environ Health*. 204, 339-346.
244. Lageman SK, Cerhan JH, Locke DE, Anderson SK, Wu W, Brown PD. (2010). Comparing neuropsychological tasks to optimize brief cognitive batteries for brain tumor clinical trials. *J Neurooncol*. 96, 271-276.
245. Landes AM, Sperry SD, Strauss ME, Geldmacher DS. (2001). Apathy in Alzheimer's disease. *J Am Geriatr Soc*. 49, 1700-1707.
246. Lang CJ, Reischies FM, Majer M, Daum RF. (1999). Visually guided exploration in Huntington disease. *Cortex*. 35, 583-590.
247. Latvala A, Castaneda AE, Perälä J, Saarni SI, Aalto-Setälä T, Lönnqvist J, Kaprio J, Suvisaari J, Tuulio-Henriksson A. (2009). Cognitive functioning in substance abuse and dependence: a population-based study of young adults. *Addiction*. 104, 1558-1568.
248. Lauterbach EC. (2005). The neuropsychiatry of Parkinson's disease. *Minerva Med*. 96, 155-173.
249. LeBlanc FN, Benson BE, Gilg AD. (1986). A severe organophosphate poisoning requiring the use of an atropine drip. *J Toxicol Clin Toxicol*. 24, 69-76.
250. Lee AH, Weintraub D. (2012). Psychosis in Parkinson's disease without dementia: common and comorbid with other non-motor symptoms. *Mov Disord*. 27, 858-863.
251. Lesser JM, Hughes S. (2006). Psychosis-related disturbances. Psychosis, agitation, and disinhibition in Alzheimer's disease: definitions and treatment options. Geriatrics. 61, 14-20.

252. Levin HS, Brown SA, Song JX, McCauley SR, Boake C, Contant CF, Goodman H, Kotrla KJ. (2001). Depression and posttraumatic stress disorder at three months after mild to moderate traumatic brain injury. *J Clin Exp Neuropsychol*. 23, 754-769.
253. Levy-Khademi F, Tenenbaum AN, Wexler ID, Amitai Y. (2007). Unintentional organophosphate intoxication in children. *Pediatr Emerg Care*. 23, 716-718.
254. Lew HL, Garvert DW, Pogoda TK, Hsu PT, Devine JM, White DK, Myers PJ, Goodrich GL. (2009). Auditory and visual impairments in patients with blast-related traumatic brain injury: Effect of dual sensory impairment on Functional Independence Measure. *J Rehabil Res Dev*. 46, 819-826.
255. Lew HL, Jerger JF, Guillory SB, Henry JA. (2007). Auditory dysfunction in traumatic brain injury. *J Rehabil Res Dev*. 44, 921-928.
256. Lewin AB, Storch EA, Mutch PJ, Murphy TK. (2011). Neurocognitive functioning in youth with pediatric autoimmune neuropsychiatric disorders associated with streptococcus. *J Neuropsychiatry Clin Neurosci*. 23, 391-398.
257. Lillo P, Garcin B, Hornberger M, Bak TH, Hodges JR. (2010). Neurobehavioral features in frontotemporal dementia with amyotrophic lateral sclerosis. *Arch Neurol*. 67, 826-830.
258. Lin G, Lawrence R. (2006). Pediatric case report of topiramate toxicity. *Clin Toxicol (Phila)*. 44, 67-69.
259. Lincoln NB, Brinkmann N, Cunningham S, Dejaeger E, De Weerdt W, Jenni W, Mahdzir A, Putman K, Schupp W, Schuback B, De Wit L. (2013). Anxiety and depression after stroke: a 5 year follow-up. *Disabil Rehabil*. 35, 140-145.
260. Liu A, Werner K, Roy S, Trojanowski JQ, Morgan-Kane U, Miller BL, Rankin KP. (2009). A case study of an emerging visual artist with frontotemporal lobar degeneration and amyotrophic lateral sclerosis. *Neurocase*. 15, 235-247.
261. Lo Coco D, La Bella V. (2012). Fatigue, sleep, and nocturnal complaints in patients with amyotrophic lateral sclerosis. *Eur J Neurol*. 19, 760-763.
262. Lorentz IT. (1989). A survey of headache in Parkinson's disease. *Cephalalgia*. 9, 83-86.
263. Lucas S, Hoffman JM, Bell KR, Walker W, Dikmen S. (2012). Characterization of headache after traumatic brain injury. *Cephalalgia*. 32, 600-606.
264. Lulé D, Diekmann V, Anders S, Kassubek J, Kübler A, Ludolph AC, Birbaumer N. (2007). Brain responses to emotional stimuli in patients with amyotrophic lateral sclerosis (ALS). *J Neurol*. 254, 519-527.
265. Lyketsos CG, Corazzini K, Steele C. (1995). Mania in Alzheimer's disease. *J Neuropsychiatry Clin Neurosci*. 7, 350-352.
266. Macerollo A, Martino D. (2013). Pediatric Autoimmune Neuropsychiatric Disorders Associated with Streptococcal Infections (PANDAS): An Evolving Concept. *Tremor Other Hyperkinet Mov (N Y)*. 25, 3. pii: tre-03-167-4158-7.
267. MacNeal JJ, Cone DC, Sinha V, Tomassoni AJ. (2012). Use of haloperidol in PCP-intoxicated individuals. *Clin Toxicol (Phila).* 50, 851-853.
268. Madhusoodanan S, Danan D, Brenner R, Bogunovic O. (2004). Brain tumor and psychiatric manifestations: a case report and brief review. *Ann Clin Psychiatry*. 16, 111-113.

269. Maia AF, Pinto AS, Barbosa ER, Menezes PR, Miguel EC. (2003). Obsessive-compulsive symptoms, obsessive-compulsive disorder, and related disorders in Parkinson's disease. *J Neuropsychiatry Clin Neurosci*. 15, 371-374.
270. Mainio A, Hakko H, Niemelä A, Koivukangas J, Räsänen P. (2011). Depression in relation to anxiety, obsessionality and phobia among neurosurgical patients with a primary brain tumor: a 1-year follow-up study. *Clin Neurol Neurosurg*. 113, 649-653.
271. Mainio A, Hakko H, Niemelä A, Koivukangas J, Räsänen P. (2013). Insomnia among brain tumor patients: a population-based prospective study of tumor patients in northern Finland. *J Psychosoc Oncol*. 31, 507-516.
272. Mainio A, Hakko H, Niemelä A, Koivukangas J, Räsänen P. (2009). Somatization symptoms are related to right-hemispheric primary brain tumor: a population-based prospective study of tumor patients in northern Finland. *Psychosomatics*. 50, 331-335.
273. Maio RF, Waller PF, Blow FC, Hill EM, Singer KM. (1997). Alcohol abuse/dependence in motor vehicle crash victims presenting to the emergency department. *Acad Emerg Med.* 4, 256-262.
274. Maltbie AA, Wingfield MS, Volow MR, Weiner RD, Sullivan JL, Cavenar JO Jr. (1980). Electroconvulsive therapy in the presence of brain tumor. Case reports and an evaluation of risk. *J Nerv Ment Dis*. 168, 400-405.
275. Manshadi M, Lippmann S, O'Daniel RG, Blackman A. (1983). Alcohol abuse and attention deficit disorder. *J Clin Psychiatry*. 44, 379-380.
276. Mapstone M, Duffy CJ. (2010). Approaching objects cause confusion in patients with Alzheimer's disease regarding their direction of self-movement. *Brain*. 133, 2690-2701.
277. March JS, Vitiello B. (2001). Advances in paediatric neuropsychopharmacology: an overview. *Int J Neuropsychopharmacol*. 4, 141-147.
278. March JS. (2004). Pediatric Autoimmune Neuropsychiatric Disorders Associated With Streptococcal Infection (PANDAS): implications for clinical practice. *Arch Pediatr Adolesc Med*. 158, 927-929.
279. Marchetti C, Carey D, Della Sala S. (2005). Crossed right hemisphere syndrome following left thalamic stroke. *J Neurol*. 252, 403-411.
280. Marchetti M, Piccione F, Silvoni S, Gamberini L, Priftis K. (2013). Covert visuospatial attention orienting in a brain-computer interface for amyotrophic lateral sclerosis patients. *Neurorehabil Neural Repair*. 27, 430-438.
281. Marshall JR. (1997). Alcohol and substance abuse in panic disorder. *J Clin Psychiatry.* 58, 46-49.
282. Marshall JR. (1994). The diagnosis and treatment of social phobia and alcohol abuse. *Bull Menninger Clin*. 58, A58-66.
283. Martinelli P, Ambrosetto G, Minguzzi E, Battaglia S, Rizzo G, Scaglione C. (2002). Late-onset PANDAS syndrome with abdominal muscle involvement. *Eur Neurol*. 48, 49-51.
284. Martínez-Horta S, Pagonabarraga J, Fernández de Bobadilla R, García-Sanchez C, Kulisevsky J. (2013). Apathy in Parkinson's disease: more than just executive dysfunction. J *Int Neuropsychol Soc*. 19, 571-582.
285. Mathias JL, Coats JL. (1999). Emotional and cognitive sequelae to mild traumatic brain injury. *J Clin Exp Neuropsychol.* 21, 200-215.

286. Max JE, Sharma A, Qurashi MI. (2002). Traumatic brain injury in a child psychiatry inpatient population: a controlled study. *J Am Acad Child Adolesc Psychiatry*. 36, 1595-1601.
287. McAllister TW, Ferrell RB. (2002). Evaluation and treatment of psychosis after traumatic brain injury. *NeuroRehabilitation*. 17, 357-368.
288. McCurry SM, Gibbons LE, Logsdon RG, Vitiello MV, Teri L. (2005). Nighttime insomnia treatment and education for Alzheimer's disease: a randomized, controlled trial. *J Am Geriatr Soc.* 53, 793-802.
289. McFarland RB, Reigel H. (1978). Chronic mercury poisoning from a single brief exposure. *J Occup Med*. 20, 532-534.
290. McFarlane AC, Hobbin ER, Kneebone CS. (1987). The determinants of illness behaviour in stroke patients. *Psychiatr Med*. 5, 133-141.
291. McFarlane AC. (1998). Epidemiological evidence about the relationship between PTSD and alcohol abuse: the nature of the association. *Addict Behav*. 23, 813-825.
292. McHugh L, Wood RL. (2008). Using a temporal discounting paradigm to measure decision-making and impulsivity following traumatic brain injury: a pilot study. *Brain Inj*. 22, 715-721.
293. McHugh T, Laforce R Jr, Gallagher P, Quinn S, Diggle P, Buchanan L. (2006). Natural history of the long-term cognitive, affective, and physical sequelae of mild traumatic brain injury. *Brain Cogn*. 60, 209-211.
294. McKinnon K, Rosner J. (2000). Severe mental illness and HIV-AIDS. *New Dir Ment Health Serv*. 87, 69-76.
295. Mclellan AT, Druley KA, Carson JE. (1978). Evaluation of substance abuse problems in a psychiatric hospital. *J Clin Psychiatry*. 39, 425-430.
296. McMurtray AM, Sultzer DL, Monserratt L, Yeo T, Mendez MF. (2008). Content-specific delusions from right caudate lacunar stroke: association with prefrontal hypometabolism. *J Neuropsychiatry Clin Neurosci.* 20, 62-67.
297. Meek PS, Clark HW, Solana VL. (1989). Neurocognitive impairment: the unrecognized component of dual diagnosis in substance abuse treatment. *J Psychoactive Drugs*. 21, 153-160.
298. Meier SL, Charleston AJ, Tippett LJ. (2010). Cognitive and behavioural deficits associated with the orbitomedial prefrontal cortex in amyotrophic lateral sclerosis. *Brain*. 133, 3444-3457.
299. Mendez MF, Owens EM, Jimenez EE, Peppers D, Licht EA. (2013). Changes in personality after mild traumatic brain injury from primary blast vs. blunt forces. *Brain Inj*. 27, 10-18.
300. Merchant RE, Merchant LH, Cook SH, McVicar DW, Young HF. (1988). Intralesional infusion of lymphokine-activated killer (LAK) cells and recombinant interleukin-2 (rIL-2) for the treatment of patients with malignant brain tumor. *Neurosurgery*. 23, 725-732.
301. Merrill DG, Mihm FG. (1982). Prolonged toxicity of organophosphate poisoning. *Crit Care Med.* 10, 550-551.
302. Micieli G, Tassorelli C, Martignoni E, Pacchetti C, Bruggi P, Magri M, Nappi G. (1991). Disordered pupil reactivity in Parkinson's disease. *Clin Auton Res*. 1, 55-58.

303. Midtling JE, Barnett PG, Coye MJ, Velasco AR, Romero P, Clements CL, O'Malley MA, Tobin MW, Rose TG, Monosson IH. (1985). Clinical management of field worker organophosphate poisoning. *West J Med*. 142, 514-518.
304. Miglioretti M, Mazzini L, Oggioni GD, Testa L, Monaco F. (2008). Illness perceptions, mood and health-related quality of life in patients with amyotrophic lateral sclerosis. *J Psychosom Res*. 65, 603-609.
305. Miller KK, Combs SA, Van Puymbroeck M, Altenburger PA, Kean J, Dierks TA, Schmid AA. (2013). Fatigue and pain: relationships with physical performance and patient beliefs after stroke. *Top Stroke Rehabil*. 20, 347-355.
306. Millis SR, Rosenthal M, Novack TA, Sherer M, Nick TG, Kreutzer JS, High WM Jr, Ricker JH. (2001). Long-term neuropsychological outcome after traumatic brain injury. *J Head Trauma Rehabil*. 16, 343-355.
307. Miyoshi K, Ueki A, Nagano O. (1996). Management of psychiatric symptoms of Parkinson's disease. *Eur Neurol*. 36, 49-58.
308. Moazzam M, Al-Saigul AM, Naguib M, Al Alfi MA. (2009). Pattern of acute poisoning in Al-Qassim region: a surveillance report from Saudi Arabia, 1999-2003. *East Mediterr Health J*. 15, 1005-1010.
309. Mochel F, N'Guyen TM, Deelchand D, Rinaldi D, Valabregue R, Wary C, Carlier PG, Durr A, Henry PG. (2012). Abnormal response to cortical activation in early stages of Huntington disease. *Mov Disord*. 27, 907-910.
310. Mollet GA, Harrison DW, Walters RP, Foster PS. (2007). Asymmetry in the emotional content of lateralised multimodal hallucinations following right thalamic stroke. *Cogn Neuropsychiatry*. 12, 422-436.
311. Monga TN, Monga M, Raina MS, Hardjasudarma M. (1986). Hypersexuality in stroke. *Arch Phys Med Rehabil*. 67, 415-417.
312. Moro E, Schwalb JM, Piboolnurak P, Poon YY, Hamani C, Hung SW, Arenovich T, Lang AE, Chen R, Lozano AM. (2011).Unilateral subdural motor cortex stimulation improves essential tremor but not Parkinson's disease. *Brain*. 134, 2096-2105.
313. Moss HB. (1989). Psychopathy, aggression, and family history in male veteran substance abuse patients: a factor analytic study. *Addict Behav*. 14, 565-570.
314. Moss HE, McCluskey L, Elman L, Hoskins K, Talman L, Grossman M, Balcer LJ, Galetta SL, Liu GT. (2012). Cross-sectional evaluation of clinical neuro-ophthalmic abnormalities in an amyotrophic lateral sclerosis population. *J Neurol Sci*. 314, 97-101.
315. Mouridsen SE, Hauschild KM. (2011). Substance use disorders: findings from a longitudinal study of individuals with and without a history of developmental language disorders. *Folia Phoniatr Logop*. 63, 195-200.
316. Mukherjee SC, Saha KC, Pati S, Dutta RN, Rahman MM, Sengupta MK, Ahamed S, Lodh D, Das B, Hossain MA, Nayak B, Mukherjee A, Chakraborti D, Dulta SK, Palit SK, Kaies I, Barua AK, Asad KA. (2005). Murshidabad--one of the nine groundwater arsenic-affected districts of West Bengal, India. Part II: dermatological, neurological, and obstetric findings. *Clin Toxicol (Phila)*. 43, 835-848.

317. Murphy DA, Greenwell L, Mouttapa M, Brecht ML, Schuster MA. (2006). Physical health of mothers with HIV/AIDS and the mental health of their children. *J Dev Behav Pediatr*. 27, 386-395.
318. Murphy ML, Pichichero ME. (2002). Prospective identification and treatment of children with pediatric autoimmune neuropsychiatric disorder associated with group A streptococcal infection (PANDAS). *Arch Pediatr Adolesc Med*. 156, 356-361.
319. Murphy TK, Snider LA, Mutch PJ, Harden E, Zaytoun A, Edge PJ, Storch EA, Yang MC, Mann G, Goodman WK, Swedo SE. (2007). Relationship of movements and behaviors to Group A Streptococcus infections in elementary school children. *Biol Psychiatry*. 61, 279-284.
320. Murphy TK, Storch EA, Lewin AB, Edge PJ, Goodman WK. (2012). Clinical factors associated with pediatric autoimmune neuropsychiatric disorders associated with streptococcal infections. *J Pediatr*. 160, 314-319.
321. Muttray A, Schneider M, Letzel S. (2012). Intoxication with a tropenol ester. *Occup Med (Lond)*. 62, 305-307.
322. Myint PK, Staufenberg EF, Sabanathan K. (2006). Post-stroke seizure and post-stroke epilepsy. *Postgrad Med J*. 82, 568-572.
323. Nagy H, Kéri S, Myers CE, Benedek G, Shohamy D, Gluck MA. (2007). Cognitive sequence learning in Parkinson's disease and amnestic mild cognitive impairment: Dissociation between sequential and non-sequential learning of associations. *Neuropsychologia*. 45, 1386-1392.
324. Nakase-Richardson R, Sherer M, Barnett SD, Yablon SA, Evans CC, Kretzmer T, Schwartz DJ, Modarres M. (2013). Prospective evaluation of the nature, course, and impact of acute sleep abnormality after traumatic brain injury. *Arch Phys Med Rehabil*. 94, 875-882.
325. Nakase-Thompson R, Sherer M, Yablon SA, Nick TG, Trzepacz PT. (2004). Acute confusion following traumatic brain injury. *Brain Inj*. 18, 131-142.
326. Narasimhalu K, Wiryasaputra L, Sitoh YY, Kandiah N. (2013). Post-stroke subjective cognitive impairment is associated with acute lacunar infarcts in the basal ganglia. *Eur J Neurol*. 20, 547-551.
327. Nazem S, Siderowf AD, Duda JE, Brown GK, Ten Have T, Stern MB, Weintraub D. (2008). Suicidal and death ideation in Parkinson's disease. *Mov Disord*. 23, 1573-1579.
328. Neufang S, Akhrif A, Riedl V, Förstl H, Kurz A, Zimmer C, Sorg C, Wohlschläger AM. (2011). Disconnection of frontal and parietal areas contributes to impaired attention in very early Alzheimer's disease. *J Alzheimers Dis*. 25, 309-321.
329. Newton HB. (1995). Common neurologic complications of HIV-1 infection and AIDS. *Am Fam Physician*. 51, 387-398.
330. Nijboer F, Birbaumer N, Kübler A. (2010). The influence of psychological state and motivation on brain-computer interface performance in patients with amyotrophic lateral sclerosis - a longitudinal study. *Front Neurosci*. 4. doi:pii: 55. 10.3389/fnins.2010.00055.
331. Noble JM, Canoll P, Honig LS. (2005). Brain tumor-associated dementia. *Sci Aging Knowledge Environ*. 2005, dn2.

332. Norman LR, Basso M, Kumar A, Malow R. (2009). Neuropsychological consequences of HIV and substance abuse: a literature review and implications for treatment and future research. *Curr Drug Abuse Rev*. 2, 143-156.
333. Nowak DA, Hermsdörfer J. (2002). Impaired coordination between grip force and load force in amyotrophic lateral sclerosis: a case-control study. *Amyotroph Lateral Scler Other Motor Neuron Disord*. 3, 199-207.
334. Ochs G, Penn RD, York M, Giess R, Beck M, Tonn J, Haigh J, Malta E, Traub M, Sendtner M, Toyka KV. (2000). A phase I/II trial of recombinant methionyl human brain derived neurotrophic factor administered by intrathecal infusion to patients with amyotrophic lateral sclerosis. *Amyotroph Lateral Scler Other Motor Neuron Disord*. 1, 201-206.
335. Odiase FE, Ogunrin OA, Ogunniyi AA. (2007). Memory performance in HIV/AIDS--a prospective case control study. *Can J Neurol Sci.* 34, 154-159.
336. O'Donnell J. (2003). Overview of existing research and information linking isotretinoin (accutane), depression, psychosis, and suicide. *Am J Ther*. 10, 148-159.
337. Ogunrin AO, Odiase FE, Ogunniyi A. (2007). Reaction time in patients with HIV/AIDS and correlation with CD4 count: a case-control study. *Trans R Soc Trop Med Hyg*. 101, 517-522.
338. Ojo JO, Abdullah L, Evans J, Reed JM, Montague H, Mullan MJ, Crawford FC. (2013). Exposure to an organophosphate pesticide, individually or in combination with other Gulf War agents, impairs synaptic integrity and neuronal differentiation, and is accompanied by subtle microvascular injury in a mouse model of Gulf War agent exposure. *Neuropathology*. Sep 30. doi: 10.1111/neup.12061.
339. Okun MS, Thommi N. (2004). Americo Negrette (1924 to 2003): diagnosing Huntington disease in Venezuela. *Neurology*. 63, 340-343.
340. Olley BO, Zeier MD, Seedat S, Stein DJ. (2005). Post-traumatic stress disorder among recently diagnosed patients with HIV/AIDS in South Africa. *AIDS Care*. 17, 550-557.
341. Olney NT, Goodkind MS, Lomen-Hoerth C, Whalen PK, Williamson CA, Holley DE, Verstaen A, Brown LM, Miller BL, Kornak J, Levenson RW, Rosen HJ. (2011). Behaviour, physiology and experience of pathological laughing and crying in amyotrophic lateral sclerosis. *Brain*. 134, 3458-3469.
342. O'Malley MA, McCurdy SA. (1990). Subacute poisoning with phosalone, an organophosphate insecticide. *West J Med*. 153, 619-624.
343. Onen SH, Onen F, Mangeon JP, Abidi H, Courpron P, Schmidt J. (2005). Alcohol abuse and dependence in elderly emergency department patients. *Arch Gerontol Geriatr*. 41, 191-200.
344. Onofrj M, Thomas A, D'Andreamatteo G, Iacono D, Luciano AL, Di Rollo A, Di Mascio R, Ballone E, Di Iorio A. (2002). Incidence of RBD and hallucination in patients affected by Parkinson's disease: 8-year follow-up. *Neurol Sci*. 23, S91-94.
345. Orvidas LJ, Slattery MJ. (2001). Pediatric autoimmune neuropsychiatric disorders and streptococcal infections: role of otolaryngologist. *Laryngoscope*. 111, 1515-1519.
346. Ostwald SK, Bernal MP, Cron SG, Godwin KM. (2009). Stress experienced by stroke survivors and spousal caregivers during the first year after discharge from inpatient rehabilitation. *Top Stroke Rehabil*. 16, 93-104.

347. Otani N, Ishimatsu S, Mochizuki T. (2008). Acute group poisoning by titanium dioxide: inhalation exposure may cause metal fume fever. *Am J Emerg Med*. 26, 608-611.
348. Ouyang MW, McDonagh DL, Phillips-Bute B, James ML, Friedman AH, Gan TJ. (2013). Comparison of postoperative nausea between benign and malignant brain tumor patients undergoing awake craniotomy: a retrospective analysis. *Curr Med Res Opin*. 29, 1039-1044.
349. Overholser JC, Freiheit SR, DiFilippo JM. (1997). Emotional distress and substance abuse as risk factors for suicide attempts. *Can J Psychiatry*. 42, 402-408.
350. Owens PL, Mutter R, Stocks C. (2010). *Mental Health and Substance Abuse-Related Emergency Department Visits among Adults, 2007: Statistical Brief #92*. Healthcare Cost and Utilization Project (HCUP) Statistical Briefs [Internet]. Rockville (MD): Agency for Health Care Policy and Research (US). 2010 Jul.
351. Ownby RL, Harwood DG, Barker WW, Duara R. (2000). Predictors of anxiety in patients with Alzheimer's disease. *Depress Anxiety*. 11, 38-42.
352. Ozyurt G, Kaya FN, Kahveci F, Alper E. (2008). Comparison of SPECT findings and neuropsychological sequelae in carbon monoxide and organophosphate poisoning. *Clin Toxicol (Phila)*. 46, 218-221.
353. Pache M, Smeets CH, Gasio PF, Savaskan E, Flammer J, Wirz-Justice A, Kaiser HJ. (2003). Colour vision deficiencies in Alzheimer's disease. *Age Ageing*. 32, 422-426.
354. Packer RJ, Gurney JG, Punyko JA, Donaldson SS, Inskip PD, Stovall M, Yasui Y, Mertens AC, Sklar CA, Nicholson HS, Zeltzer LK, Neglia JP, Robison LL. (2003). Long-term neurologic and neurosensory sequelae in adult survivors of a childhood brain tumor: childhood cancer survivor study. *J Clin Oncol*. 21, 3255-3261.
355. Palmieri A, Soraràu G, Albertini E, Semenza C, Vottero-Ris F, D'Ascenzo C, Querin G, Zennaro A, Pegoraro E, Angelini C. (2010). Psychopathological features and suicidal ideation in amyotrophic lateral sclerosis patients. *Neurol Sci*. 31, 735-740.
356. Park HK, Lim YM, Kim JS, Lee MC, Kim SM, Kim BJ, Kim KK. (2011). Nigrostriatal dysfunction in patients with amyotrophic lateral sclerosis and parkinsonism. *J Neurol Sci*. 301, 12-13.
357. Partinen M. (1997). Sleep disorder related to Parkinson's disease. *J Neurol*. 244, S3-6.
358. Pasic Z, Smajlovic D, Dostovic Z, Kojic B, Selmanovic S. (2011). Incidence and types of sleep disorders in patients with stroke. *Med Arh*. 65, 225-227.
359. Pastor MA, Day BL, Marsden CD. (1993). Vestibular induced postural responses in Parkinson's disease. *Brain*. 116, 1177-1190.
360. Patzold T, Brüne M. (2002). Obsessive compulsive disorder in huntington disease: a case of isolated obsessions successfully treated with sertraline. *Neuropsychiatry Neuropsychol Behav Neurol*. 15, 216-219.
361. Perney P, Lehert P, Mason BJ. (2012). Sleep disturbance in alcoholism: proposal of a simple measurement, and results from a 24-week randomized controlled study of alcohol-dependent patients assessing acamprosate efficacy. *Alcohol Alcohol*. 47, 133-139.
362. Péron J, Biseul I, Leray E, Vicente S, Le Jeune F, Drapier S, Drapier D, Sauleau P, Haegelen C, Vérin M. (2010). Subthalamic nucleus stimulation affects fear and sadness recognition in Parkinson's disease. *Neuropsychology*. 24, 1-8.

363. Perrin EM, Murphy ML, Casey JR, Pichichero ME, Runyan DK, Miller WC, Snider LA, Swedo SE. (2004). Does group A beta-hemolytic streptococcal infection increase risk for behavioral and neuropsychiatric symptoms in children? *Arch Pediatr Adolesc Med*. 158, 848-856.
364. Perugi G, Frare F, Madaro D, Maremmani I, Akiskal HS. (2002). Alcohol abuse in social phobic patients: is there a bipolar connection? *J Affect Disord*. 68, 33-39.
365. Peskind ER, Brody D, Cernak I, McKee A, Ruff RL. (2013). Military- and sports-related mild traumatic brain injury: an overview. *J Clin Psychiatry*. 74, e17.
366. Phukan J, Pender NP, Hardiman O. (2007). Cognitive impairment in amyotrophic lateral sclerosis. *Lancet Neurol*. 6, 994-1003.
367. Pieri V, Diederich NJ, Raman R, Goetz CG. (2000). Decreased color discrimination and contrast sensitivity in Parkinson's disease. *J Neurol Sci*. 172, 7-11.
368. Pizon AF, Schwartz AR, Shum LM, Rittenberger JC, Lower DR, Giannoutsos S, Virji MA, Krasowski MD. (2009). Toxicology laboratory analysis and human exposure to p-chloroaniline. *Clin Toxicol (Phila).* 47, 132-136.
369. Poddighe S, Bhat KM, Setzu MD, Solla P, Angioy AM, Marotta R, Ruffilli R, Marrosu F, Liscia A. (2013). Impaired sense of smell in a Drosophila Parkinson's model. *PLoS One*. 8, e73156.
370. Poloni M, Mazzini L, Ceroni M, Tosca P, Bezzi G. (1986). Motor and psychomotor functions in amyotrophic lateral sclerosis evaluated by tests of motor ability. *Ital J Neurol Sci.* 7, 63-69.
371. Pompili M, Innamorati M, Lester D, Akiskal HS, Rihmer Z, del Casale A, Amore M, Girardi P, Tatarelli R. (2009). Substance abuse, temperament and suicide risk: evidence from a case-control study. *J Addict Dis*. 28, 13-20.
372. Pontone GM, Palanci J, Bienvenu OJ, Liang KY, Nestadt G, Rabins PV, Williams JR, Marsh L. (2011). Familial aggregation of panic disturbances in Parkinson's disease. *J Neuropsychiatry Clin Neurosci.* 23, 417-424.
373. Powell JM, Ferraro JV, Dikmen SS, Temkin NR, Bell KR. (2008). Accuracy of mild traumatic brain injury diagnosis. *Arch Phys Med Rehabil*. 89, 1550-1555.
374. Preobrazhenskaya IS, Mkhitaryan EA, Yakhno NN. (2006). Comparative analysis of cognitive impairments in lewy body dementia and Alzheimer's disease. *Neurosci Behav Physiol*. 36, 1-6.
375. Price BH, Gurvit H, Weintraub S, Geula C, Leimkuhler E, Mesulam M. (1993). Neuropsychological patterns and language deficits in 20 consecutive cases of autopsy-confirmed Alzheimer's disease. *Arch Neurol*. 50, 931-937.
376. Pullicino P, Aquilina J. (1989). Opsoclonus in organophosphate poisoning. *Arch Neurol*. 46, 704-705.
377. Quaid KA, Morris M. (1993). Reluctance to undergo predictive testing: the case of Huntington disease. *Am J Med Genet*. 45, 41-45.
378. Quintana-Ortiz RA, Gomez MA, Báez Feliciano DV, Hunter-Mellado RF. (2008). Suicide attempts among Puerto Rican men and women with HIV/AIDS: a study of prevalence and risk factors. *Ethn Dis*. 18, S2-219-24.

379. Rabins PV, Starkstein SE, Robinson RG. (1991). Risk factors for developing atypical (schizophreniform) psychosis following stroke. *J Neuropsychiatry Clin Neurosci*. 3, 6-9.
380. Rabkin JG, Ferrando SJ, van Gorp W, Rieppi R, McElhiney M, Sewell M. (2000). Relationships among apathy, depression, and cognitive impairment in HIV/AIDS. *J Neuropsychiatry Clin Neurosci*. 12, 451-457.
381. Racciatti D, Vecchiet J, Ceccomancini A, Ricci F, Pizzigallo E. (2001). Chronic fatigue syndrome following a toxic exposure. *Sci Total Environ*. 270, 27-31.
382. Rasmussen A, Macias R, Yescas P, Ochoa A, Davila G, Alonso E. (2000). Huntington disease in children: genotype-phenotype correlation. *Neuropediatrics*. 31, 190-194.
383. Rathi M, Sakhuja V, Jha V. (2006). Visual blurring and metabolic acidosis after ingestion of bootlegged alcohol. *Hemodial Int*. 10, 8-14.
384. Rauber-Lüthy C, Kupferschmidt H. (2010). Household chemicals: management of intoxication and antidotes. *XS*. 100, 339-363.
385. Red RT, Richards SM, Torres C, Adair CD. (2011). Environmental toxicant exposure during pregnancy. *Obstet Gynecol Surv*. 66, 159-169.
386. Repka MX, Claro MC, Loupe DN, Reich SG. (1996). Ocular motility in Parkinson's disease. *J Pediatr Ophthalmol Strabismus*. 33, 144-147.
387. Rey C, Reinecke HJ, Besser R. (1984). Methyltin intoxication in six men; toxicologic and clinical aspects. *Vet Hum Toxicol*. 26, 121-122.
388. Rey JW, Heister P, Wirges U, Nadalin S, Breuer R, Niehues T. (2009). Organ donor with unclear primary brain tumor, a contraindication for transplantation? Case report of a one year old child. *Klin Padiatr*. 221, 390-392.
389. Ribaï P, Nguyen K, Hahn-Barma V, Gourfinkel-An I, Vidailhet M, Legout A, Dodé C, Brice A, Dürr A. (2007). Psychiatric and cognitive difficulties as indicators of juvenile huntington disease onset in 29 patients. *Arch Neurol*. 64, 813-819.
390. Rivera JA, Rivera M. (1990). Organophosphate poisoning. *Bol Asoc Med P R*. 82, 419-422.
391. Robson WJ. (1984). Alcohol abuse in adolescence. *Alcohol Alcohol*. 19, 177-179.
392. Rochat L, Billieux J, Juillerat Van der Linden AC, Annoni JM, Zekry D, Gold G, Van der Linden M. (2013). A multidimensional approach to impulsivity changes in mild Alzheimer's disease and control participants: cognitive correlates. *Cortex*. 49, 90-100.
393. Rogers SN, Vale JA. (1993). Oral manifestations of poisoning. *Br Dent J*. 174, 141-143.
394. Rohde K, Peskind ER, Raskind MA. (1995). Suicide in two patients with Alzheimer's disease. *J Am Geriatr Soc*. 43, 187-189.
395. Romesser J, Booth J, Benge J, Pastorek N, Helmer D. (2012). Mild traumatic brain injury and pain in Operation Iraqi Freedom/Operation Enduring Freedom veterans. *J Rehabil Res Dev*. 49, 1127-1136.
396. Rosenfeld JV, McFarlane AC, Bragge P, Armonda RA, Grimes JB, Ling GS. (2013). Blast-related traumatic brain injury. *Lancet Neurol*. 12, 882-893.
397. Rossit S, Malhotra P, Muir K, Reeves I, Duncan G, Harvey M. (2011). The role of right temporal lobe structures in off-line action: evidence from lesion-behavior mapping in stroke patients. *Cereb Cortex*. 21, 2751-2761.

398. Rothlind JC, Greenfield TM, Bruce AV, Meyerhoff DJ, Flenniken DL, Lindgren JA, Weiner MW. (2005). Heavy alcohol consumption in individuals with HIV infection: effects on neuropsychological performance. *J Int Neuropsychol Soc*. 11, 70-83.
399. Rothman SM, Mattson MP. (2012). Sleep disturbances in Alzheimer's and Parkinson's diseases. *Neuromolecular Med*. 14, 194-204.
400. Rowe F; VIS Group UK. (2013). Symptoms of stroke-related visual impairment. *Strabismus*. 21, 150-154.
401. Ruiz P, Guynn RW, Matorin AA. (2000). Psychiatric considerations in the diagnosis, treatment, and prevention of HIV/AIDS. *J Psychiatr Pract*. 6, 129-139.
402. Ruocco HH, Lopes-Cendes I, Laurito TL, Li LM, Cendes F. (2006). Clinical presentation of juvenile Huntington disease. *Arq Neuropsiquiatr*. 64, 5-9.
403. Rupp CI, Fleischhacker WW, Hausmann A, Mair D, Hinterhuber H, Kurz M. (2004). Olfactory functioning in patients with alcohol dependence: impairments in odor judgements. *Alcohol Alcohol*. 39, 514-519.
404. Ryb GE, Dischinger PC, Kufera JA, Read KM. (2006). Risk perception and impulsivity: association with risky behaviors and substance abuse disorders. *Accid Anal Prev*. 38, 567-573.
405. Sadeghian H, O'Suilleabhain PE, Battiste J, Elliott JL, Trivedi JR. (2011). Huntington chorea presenting with motor neuron disease. *Arch Neurol.* 68, 650-652.
406. Sadhasivam S, Litman RS. (2006). Pediatric autoimmune neuropsychiatric disorders associated with streptococcal infections - anesthetic implications and literature review. *Paediatr Anaesth*. 16, 573-577.
407. Safaz I, Alaca R, Yasar E, Tok F, Yilmaz B. (2008). Medical complications, physical function and communication skills in patients with traumatic brain injury: a single centre 5-year experience. *Brain Inj*. 22, 733-739.
408. Sahoo S. (2010). HIV- and AIDS-related Ocular Manifestations in Tanzanian Patients. *Malays J Med Sci*. 17, 12-16.
409. Sakai K, Ono K, Harada H, Shima K, Notoya M, Yamada M. (2012). Parkinson's disease showing progressive conduction aphasia. *Neurol Sci*. 33, 399-402.
410. Sanchez-Carpintero R, Albesa SA, Crespo N, Villoslada P, Narbona J. (2009). A preliminary study of the frequency of anti-basal ganglia antibodies and streptococcal infection in attention deficit/hyperactivity disorder. *J Neurol*. 256, 1103-1108.
411. Santos CO, Caeiro L, Ferro JM, Albuquerque R, Luísa Figueira M. (2006). Anger, hostility and aggression in the first days of acute stroke. *Eur J Neurol*. 13, 351-358.
412. Santos CO, Caeiro L, Ferro JM, Figueira ML. (2012). A study of suicidal thoughts in acute stroke patients. *J Stroke Cerebrovasc Dis*. 21, 749-754.
413. Santos CO, Caeiro L, Ferro JM, Figueira ML. (2011). Mania and stroke: a systematic review. *Cerebrovasc Dis*. 32, 11-21.
414. Sarro L, Agosta F, Canu E, Riva N, Prelle A, Copetti M, Riccitelli G, Comi G, Filippi M. (2011). Cognitive functions and white matter tract damage in amyotrophic lateral sclerosis: a diffusion tensor tractography study. *AJNR Am J Neuroradiol*. 32, 1866-1872.

415. Satler C, Garrido LM, Sarmiento EP, Leme S, Conde C, Tomaz C. (2007). Emotional arousal enhances declarative memory in patients with Alzheimer's disease. *Acta Neurol Scand*. 116, 355-360.
416. Savage EP, Keefe TJ, Mounce LM, Heaton RK, Lewis JA, Burcar PJ. (1988). Chronic neurological sequelae of acute organophosphate pesticide poisoning. *Arch Environ Health*. 43, 38-45.
417. Saxena K, Kingston R. (1982). Acute poisoning: management protocol. *Postgrad Med*. 71, 67-77.
418. Sayed N, Culver C, Dams-O'Connor K, Hammond F, Diaz-Arrastia R. (2013). Clinical phenotype of dementia after traumatic brain injury. *J Neurotrauma*. 30, 1117-1122
419. Scherer MR, Burrows H, Pinto R, Littlefield P, French LM, Tarbett AK, Schubert MC. (2011). Evidence of central and peripheral vestibular pathology in blast-related traumatic brain injury. *Otol Neurotol*. 32, 571-580.
420. Scheutzow MH, Wiercisiewski DR. (1999). Panic disorder in a patient with traumatic brain injury: a case report and discussion. *Brain Inj*. 13, 705-714.
421. Schievink WI. (2013). Stroke and death due to spontaneous intracranial hypotension. *Neurocrit Care*. 18, 248-251.
422. Schmidt AT, Martin RB, Ozturk A, Kates WR, Wharam MD, Mahone EM, Horska A. (2010). Neuroimaging and neuropsychological follow-up study in a pediatric brain tumor patient treated with surgery and radiation. *Neurocase*. 16, 74-90.
423. Schneider RK, Robinson MJ, Levenson JL. (2002). Psychiatric presentations of non-HIV infectious diseases. Neurocysticercosis, Lyme disease, and pediatric autoimmune neuropsychiatric disorder associated with streptococcal infection. *Psychiatr Clin North Am*. 25, 1-16.
424. Schubiner H, Tzelepis A, Milberger S, Lockhart N, Kruger M, Kelley BJ, Schoener EP. (2000). Prevalence of attention-deficit/hyperactivity disorder and conduct disorder among substance abusers. *J Clin Psychiatry*. 61, 244-251.
425. Schwartz I, Tuchner M, Tsenter J, Shochina M, Shoshan Y, Katz-Leurer M, Meiner Z. (2008). Cognitive and functional outcomes of terror victims who suffered from traumatic brain injury. *Brain Inj*. 22, 255-263.
426. Scott LK, Green R, McCarthy PJ, Conrad SA. (2009). Agitation and/or aggression after traumatic brain injury in the pediatric population treated with ziprasidone. Clinical article. *J Neurosurg Pediatr*. 3, 484-487.
427. Seidl U, Lueken U, Thomann PA, Kruse A, Schröder J. (2012). Facial expression in Alzheimer's disease: impact of cognitive deficits and neuropsychiatric symptoms. *Am J Alzheimers Dis Other Demen*. 27, 100-106.
428. Serpa Neto A, Slooter AJ. (2012). Delirium detection in stroke patients. *Crit Care Med*. 40, 2266-2267.
429. Seth R, Granville-Grossman K, Goldmeier D, Lynch S. (1991). Psychiatric illnesses in patients with HIV infection and AIDS referred to the liaison psychiatrist. *Br J Psychiatry*. 159, 347-350.
430. Shadnia S, Moiensadat M, Abdollahi M. (2004). A case of acute strychnine poisoning. *Vet Hum Toxicol*. 46, 76-79.

431. Shahar E, Andraws J. (2001). Extra-pyramidal parkinsonism complicating organophosphate insecticide poisoning. *Eur J Paediatr Neurol*. 5, 261-264.
432. Shen WW. (1984). Extrapyramidal symptoms associated with alcohol withdrawal. *Biol Psychiatry*. 19, 1037-1043.
433. Sherer M, Yablon SA, Nakase-Richardson R. (2009). Patterns of recovery of posttraumatic confusional state in neurorehabilitation admissions after traumatic brain injury. *Arch Phys Med Rehabil*. 90, 1749-1754.
434. Shibata N, Kubota F, Machiyama Y, Takahashi A, Miyamoto K. (1998). Mapping epileptic foci by the dipole tracing method in a brain tumor patient with olfactory seizures: comparison with intraoperative electrocorticograms. *Clin Electroencephalogr*. 29, 91-95.
435. Shigematsu K, Shimamura O, Nakano H, Watanabe Y, Sekimoto T, Shimizu K, Nishizawa A, Makino M. (2013). Vomiting should be a prompt predictor of stroke outcome. *Emerg Med J*. 30, 728-731.
436. Shulman ST. (2009). Pediatric autoimmune neuropsychiatric disorders associated with streptococci (PANDAS): update. *Curr Opin Pediatr*. 21, 127-130.
437. Sibolt G, Curtze S, Melkas S, Putaala J, Pohjasvaara T, Kaste M, Karhunen PJ, Oksala NK, Erkinjuntti T. (2013). Poststroke dementia is associated with recurrent ischaemic stroke. *J Neurol Neurosurg Psychiatry*. 84, 722-726.
438. Siddique H, Hynan LS, Weiner MF. (2009). Effect of a serotonin reuptake inhibitor on irritability, apathy, and psychotic symptoms in patients with Alzheimer's disease. *J Clin Psychiatry*. 70, 915-918.
439. Siegel K, Schrimshaw EW, Pretter S. (2005). Stress-related growth among women living with HIV/AIDS: examination of an explanatory model. *J Behav Med*. 8, 403-414.
440. Siemers E, Foroud T, Bill DJ, Sorbel J, Norton JA Jr, Hodes ME, Niebler G, Conneally PM, Christian JC. (1996). Motor changes in presymptomatic Huntington disease gene carriers. *Arch Neurol*. 53, 487-492.
441. Simpson G, Tate R. (2002). Suicidality after traumatic brain injury: demographic, injury and clinical correlates. *Psychol Med*. 32, 687-697.
442. Simpson WM Jr. (2004). A family outbreak of acute organophosphate poisoning: a diagnostic challenge. *J Agromedicine*. 9, 391-394.
443. Sinforiani E, Terzaghi M, Pasotti C, Zucchella C, Zambrelli E, Manni R. (2007). Hallucinations and sleep-wake cycle in Alzheimer's disease: a questionnaire-based study in 218 patients. *Neurol Sci*. 28, 96-99.
444. Sinha N, Manohar S, Husain M. (2013). Impulsivity and apathy in Parkinson's disease. *J Neuropsychol*. 7, 255-283.
445. Sitek EJ, Sołtan W, Robowski P, Schinwelski M, Wieczorek D, Sławek J. (2012). Poor insight into memory impairment in patients with Huntington disease. *Neurol Neurochir Pol*. 46, 318-325.
446. Skelton WP 3rd, Skelton NK. (1991). Alzheimer's disease. Recognizing and treating a frustrating condition. *Postgrad Med*. 90, 33-34, 37-41.
447. Slovarp L, Azuma T, Lapointe L. (2012). The effect of traumatic brain injury on sustained attention and working memory. *Brain In*j. 26, 48-57.

448. Smith MC, Smith MK, Ellgring H. (1996). Spontaneous and posed facial expression in Parkinson's disease. *J Int Neuropsychol Soc*. 2, 383-391.
449. Sokolski KN, Cummings JL, Abrams BI, DeMet EM, Katz LS, Costa JF. (1994). Effects of substance abuse on hallucination rates and treatment responses in chronic psychiatric patients. *J Clin Psychiatry*. 55, 380-387.
450. Somerville B. (1994). MRI angiography of a patient before and during alcohol-induced cluster headache. *Headache*. 34, 463-466.
451. Spica V, Pekmezović T, Svetel M, Kostić VS. (2013). Prevalence of non-motor symptoms in young-onset versus late-onset Parkinson's disease. *J Neurol*. 260, 131-137.
452. Starkstein SE, Mizrahi R, Power BD. (2008). Depression in Alzheimer's disease: phenomenology, clinical correlates and treatment. *Int Rev Psychiatry*. 20, 382-388.
453. Steinbach S, Hundt W, Vaitl A, Heinrich P, Förster S, Bürger K, Zahnert T. (2010). Taste in mild cognitive impairment and Alzheimer's disease. *J Neurol*. 257, 238-246.
454. Stepkina DA, Zakharov VV, Yakhno NN. (2010). Cognitive impairments in progression of Parkinson's disease. *Neurosci Behav Physiol*. 40, 61-67.
455. Stevens S, Haynes PL, Ruiz B, Bootzin RR. (2007). Effects of a behavioral sleep medicine intervention on trauma symptoms in adolescents recently treated for substance abuse. *Subst Abus*. 28, 21-31.
456. Stevens S, Rist F, Gerlach AL. (2009). Influence of alcohol on the processing of emotional facial expressions in individuals with social phobia. *Br J Clin Psychol*. 48, 125-140.
457. Storch EA, Murphy TK, Geffken GR, Mann G, Adkins J, Merlo LJ, Duke D, Munson M, Swaine Z, Goodman WK. (2006). Cognitive-behavioral therapy for PANDAS-related obsessive-compulsive disorder: findings from a preliminary waitlist controlled open trial. *J Am Acad Child Adolesc Psychiatry*. 45, 1171-1178.
458. Strakowski SM, McElroy SL, Keck PE Jr, West SA. (1996). The effects of antecedent substance abuse on the development of first-episode psychotic mania. *J Psychiatr Res*. 30, 59-68.
459. Stulemeijer M, Vos PE, Bleijenberg G, van der Werf SP. (2007). Cognitive complaints after mild traumatic brain injury: things are not always what they seem. *J Psychosom Res*. 63, 637-645.
460. Suljic E, Mehicevic A, Gavranovic A. (2013). Stroke emergency medical care: initial assessment, risk factors, triage and hospitalization outcome. *Mater Sociomed*. 25, 83-87.
461. Sułkowski WJ, Kowalska S, Sobczak Z, Jóźwiak Z. (1992). The statokinesiometry in evaluation of the balance system in persons with chronic carbon disulphide intoxication. *Pol J Occup Med Environ Health*. 5, 265-276.
462. Suzuki K, Miyamoto M, Miyamoto T, Tatsumoto M, Watanabe Y, Suzuki S, Iwanami M, Sada T, Kadowaki T, Numao A, Trenkwalder C, Hirata K. (2012). Nocturnal disturbances and restlessness in Parkinson's disease: using the Japanese version of the Parkinson's disease sleep scale-2. *J Neurol Sci*. 318,76-81.
463. Svikis DS, Zarin DA, Tanielian T, Pincus HA. (2000). Alcohol abuse and dependence in a national sample of psychiatric patients. *J Stud Alcohol*. 61, 427-430.

464. Swedo SE, Leonard HL, Garvey M, Mittleman B, Allen AJ, Perlmutter S, Lougee L, Dow S, Zamkoff J, Dubbert BK. (1998). Pediatric autoimmune neuropsychiatric disorders associated with streptococcal infections: clinical description of the first 50 cases. *Am J Psychiatry*. 155, 264-271.
465. Swick D, Honzel N, Larsen J, Ashley V, Justus T. (2012). Impaired response inhibition in veterans with post-traumatic stress disorder and mild traumatic brain injury. *J Int Neuropsychol Soc*. 18, 917-926.
466. Takeda T, Uchihara T, Mochizuki Y, Mizutani T, Iwata M. (2007). Memory deficits in amyotrophic lateral sclerosis patients with dementia and degeneration of the perforant pathway A clinicopathological study. *J Neurol Sci*. 260, 225-230.
467. Taylor GH, Broomfield NM. (2013). Cognitive Assessment and Rehabilitation Pathway for Stroke (CARPS). *Top Stroke Rehabi*l. 20, 270-282.
468. Titus JC, Schiller JA, Guthmann D. (2008). Characteristics of youths with hearing loss admitted to substance abuse treatment. *J Deaf Stud Deaf Educ*. 13, 336-350.
469. Tómasson K, Vaglum P. (1998). Social consequences of substance abuse: the impact of comorbid psychiatric disorders. A prospective study of a nation-wide sample of treatment-seeking patients. *Scand J Soc Med.* 26, 63-70.
470. Tosto G, Talarico G, Lenzi GL, Bruno G. (2008). Effect of citalopram in treating hypersexuality in an Alzheimer's disease case. *Neurol Sci*. 29, 269-270.
471. Troost D, Louwerse ES, de Jong JM, van Leersum GS, van Raalte JA. (1989). Aberrant myelinated neurites in the anterior horns of a patient with amyotrophic lateral sclerosis. *Clin Neuropathol.* 8, 152-155.
472. Tsai MH, Tsai NW, Chen SF, Tsai HH, Lu CH, Huang CR, Chang WN. (2006). Organophosphate intoxication-related coital-like involuntary movements: report of A case. *Acta Neurol Taiwan*. 15,34-37.
473. Tsai MJ, Wu SN, Cheng HA, Wang SH, Chiang HT. (2003). An outbreak of food-borne illness due to methomyl contamination. *J Toxicol Clin Toxicol*. 41, 969-973.
474. Tsai YF, Hsiung PC, Holzemer WL. (2002). Symptom management in Taiwanese patients with HIV/AIDS. *J Pain Symptom Manage*. 23, 301-309.
475. Tull MT, Weiss NH, Adams CE, Gratz KL. (2012). The contribution of emotion regulation difficulties to risky sexual behavior within a sample of patients in residential substance abuse treatment. *Addict Behav*. 37, 1084-1092.
476. Tune LE. (1998). Depression and Alzheimer's disease. *Depress Anxiety*. 8, 91-95.
477. Urban P, Pelclová D, Lukás E, Kupka K, Preiss J, Fenclová Z, Smerhovský Z. (2007). Neurological and neurophysiological examinations on workers with chronic poisoning by 2,3,7,8-TCDD: follow-up 35 years after exposure. *Eur J Neurol*. 14, 213-218.
478. Vaishnavi S, Rao V, Fann JR. (2009). Neuropsychiatric problems after traumatic brain injury: unraveling the silent epidemic. *Psychosomatics*. 50, 198-205.
479. van Asselen M, Júlio F, Januário C, Campos EB, Almeida I, Cavaco S, Castelo-Branco M. (2012). Scanning Patterns of Faces do not Explain Impaired Emotion Recognition in Huntington Disease: Evidence for a High Level Mechanism. *Front Psychol*. 3, 31.
480. van Breemen MS, Vecht CJ. (2005). Optimal seizure management in brain tumor patients. *Curr Neurol Neurosci Rep*. 5, 207-213.

481. van den Berg C, Beek PJ, Wagenaar RC, van Wieringen PC. (2000). Coordination disorders in patients with Parkinson's disease: a study of paced rhythmic forearm movements. *Exp Brain Res*. 134, 174-186.
482. van der Westhuizen Y, Swanepoel de W, Heinze B, Hofmeyr LM. (2013). Auditory and otological manifestations in adults with HIV/AIDS. *Int J Audiol*. 52, 37-43.
483. Van Deusen J. (1989). Alcohol abuse and perceptual-motor dysfunction: the occupational therapist's role. *Am J Occup Ther*. 43, 384-390.
484. van Toorn R, Weyers HH, Schoeman JF. (2004). Distinguishing PANDAS from Sydenham's chorea: case report and review of the literature. *Eur J Paediatr Neurol*. 8, 211-216.
485. Vanderploeg RD, Schwab K, Walker WC, Fraser JA, Sigford BJ, Date ES, Scott SG, Curtiss G, Salazar AM, Warden DL; Defense and Veterans Brain Injury Center Study Group. (2008). Rehabilitation of traumatic brain injury in active duty military personnel and veterans: Defense and Veterans Brain Injury Center randomized controlled trial of two rehabilitation approaches. *Arch Phys Med Rehabil*. 89, 2227-2238.
486. Vardi J, Flechter S, Rabi JM, Streifler M. (1979). R.H.I.S.A. -- cysternography study in sporadic choreo-athetotic syndrome accompanied with dementia (sporadic Huntington disease). *Riv Patol Nerv Ment*. 100, 245-254.
487. Vaz TS, Bertolucci PH, Oliveira FF. (2012). A patient with primary progressive aphasia developing dementia due to Alzheimer's disease. *Arq Neuropsiquiatr*. 70, 551-552.
488. Veranic LZ, Pregelj P. (2008). Alcohol abuse and suicidal behaviour. *Psychiatr Danub*. 20, 236-238.
489. Verdelho A, Ferro JM, Melo T, Canhão P, Falcão F. (2008). Headache in acute stroke. A prospective study in the first 8 days. *Cephalalgia*. 28, 346-354.
490. Verma RK, Panda NK, Basu D, Raghunathan M. (2006). Audiovestibular dysfunction in alcohol dependence. Are we worried? *Am J Otolaryngol*. 27, 225-228.
491. Videnovic A, Leurgans S, Fan W, Jaglin J, Shannon KM. (2009). Daytime somnolence and nocturnal sleep disturbances in Huntington disease. *Parkinsonism Relat Disord*. 15, 471-474.
492. Vieregge P, Wauschkuhn B, Heberlein I, Hagenah J, Verleger R. (1999). Selective attention is impaired in amyotrophic lateral sclerosis--a study of event-related EEG potentials. *Brain Res Cogn Brain Res*. 8, 27-35.
493. Villano JL, Mlinarevich N, Watson KS, Engelhard HH, Anderson-Shaw L. (2009). Aggression in a patient with primary brain tumor: ethical implications for best management. *J Neurooncol*. 94, 293-296.
494. Vitale C, Marcelli V, Allocca R, Santangelo G, Riccardi P, Erro R, Amboni M, Pellecchia MT, Cozzolino A, Longo K, Picillo M, Moccia M, Agosti V, Sorrentino G, Cavaliere M, Marciano E, Barone P. (2012). Hearing impairment in Parkinson's disease: expanding the nonmotor phenotype. *Mov Disord*. 27, 1530-1535.
495. Volkow ND, Fowler JS. (1992). Neuropsychiatric disorders: investigation of schizophrenia and substance abuse. *Semin Nucl Med*. 22, 254-267.
496. Voss J, Portillo CJ, Holzemer WL, Dodd MJ. (2007). Symptom cluster of fatigue and depression in HIV/AIDS. *J Prev Interv Community*. 33, 19-34.

497. Vuletić V, Sapina L, Lozert M, Lezaić Z, Morović S. (2012). Anxiety and depressive symptoms in acute ischemic stroke. *Acta Clin Croat*. 51, 243-246.
498. Walker WC, Pickett TC. (2007). Motor impairment after severe traumatic brain injury: A longitudinal multicenter study. *J Rehabil Res Dev*. 44, 975-982.
499. Wang J, Eslinger PJ, Doty RL, Zimmerman EK, Grunfeld R, Sun X, Meadowcroft MD, Connor JR, Price JL, Smith MB, Yang QX. (2010). Olfactory deficit detected by fMRI in early Alzheimer's disease. *Brain Res*. 1357, 184-194.
500. Weaver SM, Chau A, Portelli JN, Grafman J. (2012). Genetic polymorphisms influence recovery from traumatic brain injury. *Neuroscientist*. 18, 631-644.
501. Webb A, Norton M. (2004). Clinical assessment of symptom-focused health-related quality of life in HIV/AIDS. *J Assoc Nurses AIDS Care*. 15, 67-78.
502. Weisberg LA, Ross W. (1989). AIDS dementia complex. Characteristics of a unique aspect of HIV infection. *Postgrad Med.* 86, 213-220.
503. Wellisch DK, Kaleita TA, Freeman D, Cloughesy T, Goldman J. (2002). Predicting major depression in brain tumor patients. *Psychooncology*. 11, 230-238.
504. Wender D. (1987). "Craziness" and "visions": experiences after a stroke. *Br Med J (Clin Res Ed).* 295,1595-1597.
505. Wendland KL, Danzer G. (1987). Life style and clinical findings in patients with hallucinations with or after alcohol abuse. *Schweiz Arch Neurol Psychiatr*. 138, 69-83.
506. Wetzel HH, Gehl CR, Dellefave-Castillo L, Schiffman JF, Shannon KM, Paulsen JS; Huntington Study Group. (2011). Suicidal ideation in Huntington disease: the role of comorbidity. *Psychiatry Res*. 188,372-376.
507. Weyer Jamora C, Schroeder SC, Ruff RM. (2013). Pain and mild traumatic brain injury: the implications of pain severity on emotional and cognitive functioning. *Brain Inj*. 27, 1134-1140.
508. White RF, Feldman RG, Eviator II, Jabre JF, Niles CA. (1997). Hazardous waste and neurobehavioral effects: a developmental perspective. *Environ Res*. 73, 113-124.
509. Wiggins S, Green T, Adam S, Hayden MR. (1996). A long term (ca 5 years) prospective assessment of psychological consequences of predictive testing for Huntington disease (HD) *Am J Hum Genet.* 59, A7.
510. Willey JZ, Disla N, Moon YP, Paik MC, Sacco RL, Boden-Albala B, Elkind MS, Wright CB. (2010). Early depressed mood after stroke predicts long-term disability: the Northern Manhattan Stroke Study (NOMASS). *Stroke*. 41, 1896-1900.
511. Wodarski JS. (2010). Prevention of adolescent reoccurring violence and alcohol abuse: a multiple site evaluation. *J Evid Based Soc Work*. 7, 280-301.
512. Wolffbrandt MM, Poulsen I, Engberg AW, Hornnes N. (2013). Occurrence and severity of agitated behavior after severe traumatic brain injury. *Rehabil Nurs.* 38, 133-141.
513. Wood JM, Kundu B, Utter A, Gallagher TA, Voss J, Nair VA, Kuo JS, Field AS, Moritz CH, Meyerand ME, Prabhakaran V. (2011). Impact of brain tumor location on morbidity and mortality: a retrospective functional MR imaging study. *AJNR Am J Neuroradiol*. 32(8):1420-1425.

514. Wood SM, Shah SS, Steenhoff AP, Rutstein RM. (2009). The impact of AIDS diagnoses on long-term neurocognitive and psychiatric outcomes of surviving adolescents with perinatally acquired HIV. *AIDS*. 23, 1859-1865.
515. Woolley J, Allen R, Wessely S. (2004). Alcohol use in chronic fatigue syndrome. *J Psychosom Res*. 56, 203-206.
516. Xydakis MS, Ling GS, Mulligan LP, Olsen CH, Dorlac WC. (2012). Epidemiologic aspects of traumatic brain injury in acute combat casualties at a major military medical center: a cohort study. *Ann Neurol*. 72, 673-681.
517. Yadav R, Pinto C. (2000). Mania in Parkinson's disease with treatment emergent dyskineisa : a case report. *Indian J Psychiatry*. 42, 439-441.
518. Yaddanapudi K, Hornig M, Serge R, De Miranda J, Baghban A, Villar G, Lipkin WI. (2010). Passive transfer of streptococcus-induced antibodies reproduces behavioral disturbances in a mouse model of pediatric autoimmune neuropsychiatric disorders associated with streptococcal infection. *Mol Psychiatry*. 15, 712-726.
519. Yamanishi T, Tachibana H, Oguru M, Matsui K, Toda K, Okuda B, Oka N. (2013). Anxiety and depression in patients with Parkinson's disease. *Intern Med*. 52, 539-545.
520. Yan JH, Rountree S, Massman P, Doody RS, Li H. (2008). Alzheimer's disease and mild cognitive impairment deteriorate fine movement control. *J Psychiatr Res*. 42, 1203-1212.
521. Yang CC, Deng JF. (2007). Intermediate syndrome following organophosphate insecticide poisoning. *J Chin Med Assoc*. 70, 467-472.
522. Yang CC, Hua MS, Lin WC, Tsai YH, Huang SJ. (2012). Irritability following traumatic brain injury: divergent manifestations of annoyance and verbal aggression. *Brain Inj*. 26,1185-1191.
523. Yurumez Y, Durukan P, Yavuz Y, Ikizceli I, Avsarogullari L, Ozkan S, Akdur O, Ozdemir C. (2007). Acute organophosphate poisoning in university hospital emergency room patients. *Intern Med*. 46, 965-969.
524. Zeitzer JM, Friedman L, O'Hara R. (2009). Insomnia in the context of traumatic brain injury. *J Rehabil Res Dev*. 46, 827-836.
525. Zhou MZ, Gan J, Wei YR, Ren XY, Chen W, Liu ZG. (2013). The association between non-motor symptoms in Parkinson's disease and age at onset. *Clin Neurol Neurosurg*. 115, 2103-2107.
526. Zimmerli L, Krewer C, Gassert R, Müller F, Riener R, Lünenburger L. (2012). Validation of a mechanism to balance exercise difficulty in robot-assisted upper-extremity rehabilitation after stroke. *J Neuroeng Rehabil*. 9, 6.
527. Zugman A, Pan PM, Gadelha A, Mansur RB, Asevedo E, Cunha GR, Silva PF, Brietzke E, Bressan RA. (2013). Brain tumor in a patient with attenuated psychosis syndrome. *Schizophr Res*. 144, 151-152.
528. Zwiener RJ, Ginsburg CM. (1988). Organophosphate and carbamate poisoning in infants and children. *Pediatrics*. 81, 121-126.
